# The effect of retirement on physical and mental health in China: a nonparametric fuzzy regression discontinuity study

**DOI:** 10.1186/s12889-024-18649-w

**Published:** 2024-04-27

**Authors:** Ting Wang, Huizhen Liu, Xiaoqin Zhou, Changxi Wang

**Affiliations:** 1 Center of Biostatistics, Design, Measurement and Evaluation (CBDME), Department of Clinical Research Management, West China Hospital, Sichuan University, Chengdu, Sichuan China; 2 West China Biomedical Big Data Center, West China Hospital, Sichuan University, Chengdu, Sichuan China; 3 Med-X Center for Informatics, Sichuan University, Chengdu, China; 4 Sichuan University - Pittsburgh Institute, Sichuan University, Chengdu, 610207 China

**Keywords:** Retirement, Physical health, Mental health, Chinese elderly, Regression discontinuity design, CHARLS

## Abstract

**Background:**

With the rapid aging of the domestic population, China has a strong incentive to increase the statutory retirement age. How retirement affects the health of the elderly is crucial to this policymaking. The health consequences of retirement have been debated greatly. This study aims to investigate the effects of retirement on physical and mental health among Chinese elderly people.

**Methods:**

The data we use in this study comes from four waves (2011, 2013, 2015, and 2018) of the Harmonized China Health and Retirement Longitudinal Study (Harmonized CHARLS), a prospective cohort. We use the nonparametric fuzzy regression discontinuity design to estimate the effects of retirement on physical and mental health. We test the robustness of our results with respect to different bandwidths, kernel functions, and polynomial orders. We also explore the heterogeneity across gender and education.

**Results:**

Results show that retirement has an insignificant effect on a series of physical and mental health outcomes, with and without adjusting several sociodemographic variables. Heterogeneity exists regarding gender and education. Although stratified analyses indicate that the transition from working to retirement leaves minimal effects on males and females, the effects go in the opposite direction. This finding holds for low-educated and high-educated groups for health outcomes including depression and cognitive function. Most of the results are stable with respect to different bandwidths, kernel functions, and polynomial orders.

**Conclusions:**

Our results suggest that it is possible to delay the statutory retirement age in China as retirement has insignificant effects on physical and mental health. However, further research is needed to assess the long-term effect of retirement on health.

**Supplementary Information:**

The online version contains supplementary material available at https://doi.org/10.1186/s12889-024-18649-w.

## Background

Due to the joint effect of declining fertility rates and prolonged life expectancy, population aging has become a global phenomenon, and the trend is expected to intensify in the foreseeable future. China is among the most rapidly aging countries in the world [1]. In China, the proportion of the population aged 65 and over is estimated to triple from 11.47% in 2019 to 31.79% in 2050, and the old-age dependency ratio to increase from 0.17 to 0.52 during the above-mentioned period [2].

Population aging leads to an increasing number of retired people, which imposes huge costs on public and private budgets, and leads to a shortage of working force. To solve this problem, some countries are forced to increase the pension-eligible age or statutory retirement age. For example, the United States started to gradually increase the eligibility age from 65 to 67 in 2003; in 2007, the United Kingdom increased the retirement age gradually from 65 to 68; in 2009, Australia raised the “Age Pension” age from 65 to 67 [3]. In China, the statutory retirement ages are 60 for men, 50 for blue-collar women, and 55 for white-collar women, which are among the lowest in the world. In the 14^th^ Five-year Plan and the Outline of the Vision Goals to 2023, the Chinese government plans to gradually raise the statutory retirement age [4].

The success of delaying the retirement age policy will depend partially on its effect on health and health behaviors. There are two prevailing opinions regarding the association between retirement and health. The first opinion is that people who retire early may live healthier, happier, and longer, and have improved cognitive function than those who continue working, due to relief from the adverse effects of work demands and stress, increased sleep duration, as well as more frequent physical activities [5–7]. The other opinion is that people who retire early suffer and have an increased risk of depression and cognitive impairment because they have a reduction in income, social activities, and mental activities [6–10]. International evidence on the effect of retirement on health is controversial. Some papers report that retirement has a positive effect on physical and mental health, self-assessed health status, and life satisfaction [11–17]. Some find a negative effect [16, 18–22]. The others find that there is no association between retirement and health [6, 11, 20, 23, 24]. There is also no consensus view on how health behaviors change during retirement. Some studies report that people have an increase in physical activity, and a decrease in alcohol consumption and smoking after retirement [8, 11, 12, 25–28]. Some suggest a decline in physical activity and an increase in smoking and drinking [20, 29, 30]. The others find the change in physical activity, alcohol consumption, and smoking is insignificant [11, 21, 25].

Findings on the effect of retirement on health and health behaviors in China are mixed as well. Some findings are against the delaying retirement policy. Retirement increases depression risk, as well as weight and BMI, due to a reduction of social activities, especially in people who are male and living in rural areas [18, 30]. Retirement also leads to a decline in physical health [31] and subjective health assessment [32], where the reason may be that retirement leads to a decline in physical activities among elderly Chinese residents [29]. Other research gives the opposite results. Retirement improves self-assessed health status, increases happiness [13], decreases the risk of depression [12, 33], and improves cognitive status [33]. As a result of physical deterioration, seniors may begin to pay attention to healthier lifestyle habits, such as reduced smoking and alcohol consumption and increased physical activity [27, 28, 31]. Again, some studies show that the effect of retirement on physical and mental health, and health behaviors was close to insignificant [11, 23, 24, 32].

Mixed results may be caused by the differences in health measures and biomarkers examined, institutional settings of the countries analyzed, social norms that make individuals report differently, or methodology employed to study the association [34–36]. Other than the above factors, sociodemographic characteristics also play an important role in introducing the differences in associations between retirement and health. The effect of retirement embedding gender context is ambiguous. Women in the labor market may suffer more from tremendous stress because they need to handle work and their families simultaneously. As such, retirement may relieve job stress [37] and protect women against depression and cognitive decline [9, 38]. On the other hand, retirement may impose more negative impact on women, because women may have more housework and less social interaction [9]. As women often drink and smoke less compared with men, the association between retirement and health behaviors is usually not significant in women [28]. Heterogeneity also exists across different education groups, where retirement has more considerable adverse effects on less-educated individuals compared to high-educated individuals [38].

There are several research limitations in the existing literature on the influence of retirement on health. First, as described above, there are conflicting results. Second, some studies investigated the effect of retirement under the voluntary system. However, China implements a mandatory retirement system, which may lead to differences in the effects of retirement on health. Third, different study adopts different definitions of retirement. There are three common definitions used in previous studies: (a) “reporting retired and not working”; (b) “not working for pay”; and (c) “reporting retired” [36]. We adopt the last definition in our study because we aim to explore how the retirement policy impacts health, thus whether an individual works after retirement voluntarily does not matter. Fourth, many of the studies only study a single or several health indicators, which may not comprehensively reflect the effect of retirement on health.

Therefore, in this paper, we aim to comprehensively investigate the effect of retirement on a wide range of health indicators, both physical and mental, for a sample of Chinese elderly people drawn from the Harmonized China Health and Retirement Longitudinal Study (Harmonized CHARLS). Physical health indicators include activities of daily living (ADL) and instrumental activities of daily living (IADL), which assess the functional status of seniors. According to the CDC (Centers for Disease Control and Prevention), mental health includes emotional, psychological, and social well-being [39]. We investigated mental health indicators including memory (assessed by total recall score), cognitive function (assessed by the CHARLS cognitive battery adapted from Harmonized Cognitive Assessment Protocol designed by the Health and Retirement Study (HRS-HCAP)), depression (assessed by the 10-item Center for Epidemiologic Studies Depression (CESD) scale), and life satisfaction. We also investigated subjective well-being which was assessed by self-reported general health status.

We employ a nonparametric fuzzy regression discontinuity (RD) design in this study to address the endogeneity of retirement. The RD design is a quasi-experimental design to evaluate the effects of a treatment, in our case, retirement. We follow a data-driven approach to find the optimal bandwidth and perform robustness checks for different bandwidths, kernel functions, and polynomial orders.

### Institutional background

Multiple pension policies are currently implemented in China, including pension for public servants and public institution employees, basic pension for enterprise employees, urban and rural resident pension, new rural resident pension, urban resident pension, and so on. Although both urban and rural residents enjoy old-age pension benefits, rural pension coverage is limited. Moreover, the strict retirement concept only exists in pension for public servants and public institution employees, and basic pension for enterprise employees. Residents enrolled in other pension plans mainly decide when to quit the labor market based on personal health and economic characteristics [16].

By national policy, the statutory retirement age (SRA) for male employees in both public institutions, state-owned enterprises, and private enterprises is 60, while the SRA is 55 for white-collar female employees, and 50 for blue-collar female employees. In principle, employees must administratively process retirement when they reach the SRA. However, some employees may apply for early retirement while some may work later for wages [40]. Nevertheless, the process of retirement, which always means the final exit from work, occurs much more often at the SRA.

In this study, we restrict our samples to employees who are enrolled in the pension for public servants and public institution employees, and basic pension for enterprise employees.

## Methods

### Data source

The data analyzed in this study are derived from the Harmonized China Health and Retirement Longitudinal Study (Harmonized CHARLS). CHARLS is the first nationally representative survey of the older population that enables the study of the health of the older population in China patterned after the US Health and Retirement Study (HRS). The CHARLS national baseline survey was conducted in 2011–2012, with wave 2 in 2013, wave 3 in 2015, and wave 4 in 2018. The CHARLS sample is representative of people aged 45 and over. A stratified multi-stage PPS random sampling strategy was adopted. The CHARLS questionnaire includes the following modules: demographics, family structure/transfer, health status and functioning, biomarkers, health care and insurance, work, retirement and pension, income and consumption, assets (individual and household), and community-level information [41].

Ethical approval for all the CHARLS waves was granted by the Institutional Review Board at Peking University. The IRB approval number for the main household survey, including anthropometrics, is IRB00001052-11015; the IRB approval number for biomarker collection, was IRB00001052-11014.

### Study population

This study comprises respondents from all four waves. Respondents who met the following inclusion and exclusion criteria were included in the study. Inclusion criteria: a) respondents who enrolled in pension for public servants and public institution employees, or basic pension for enterprise employees; b) males aged between 50 and 70, and females aged between 45 and 65. Exclusion criteria: a) respondents who have never worked or been employed; b) respondents who retired due to health reasons; c) respondents with missing information on health measures or controlled variables (mentioned in the following section). To make the sample size large enough to meet the requirement of RD design, we construct four-wave pooled cross-sectional data from 2011 to 2018. As multiple outcomes are assessed, the sample size is different for different outcomes. The flowchart for sample selection for each outcome variable is presented in Additional file 1: Table S1.

### Health outcomes

We analyze several health outcomes in this study to comprehensively evaluate the effect of retirement on health.

#### Self-reported health

The scale ranges from 1 for Very good to 5 for Very poor. The variable is recoded dichotomously into good (1 = very good or good or fair) and poor (0 = poor or very poor) [24, 27].

#### Activities of daily living (ADL)

Basic ADLs are the skills required to perform daily physical tasks, including bathing, dressing, eating, getting in/out of bed, using the toilet, and controlling urination. ADL score ranges from 0 to 6, where the score means the number of activities with which a respondent reported having difficulty. The higher the score, the worse the basic functional status.

#### Instrumental activities of daily living (IADL)

IADLs include more complex activities than basic ADLs, consisting of using the phone, managing money, taking medications, shopping for groceries, and preparing hot meals. IADL score ranges from 0 to 5, where the score means the number of activities with which a respondent reported having difficulty. The higher the score, the worse the independent living skills.

#### Total recall score

Total recall score is the sum of immediate recall and delayed recall. During the interview, respondents are read a list of 10 words (e.g., car, lake, book, etc.) and asked to recall as many of them as possible; first immediately after they heard the list, and then after a delayed time. Each correct word scores one point, and the index is the sum of all correct answers: it ranges from 0 to 10 for both immediate recall and delayed recall. Thus, the total recall score ranges from 0 to 20.

#### the CHARLS cognitive battery adapted from HRS-HCAP

The CHARLS cognitive battery adapted from HRS-HCAP was started to be used in wave 4 for screening cognitive function. In waves 1, 2, and 3, only items on orientation (year, date, day of the week, season), serially subtracting 7 from 100, and the drawing of overlapping pentagons were part of the regular CHARLS cognition battery. For consistency reasons, we only include the above-mentioned items in the analysis of cognitive function, which ranges from 0 to 11. The higher the score, the better the cognitive function.

#### CESD

The 10-item Center for Epidemiologic Studies Depression (CESD) scale is used to screen depressive symptoms. CESD score ranges from 0 to 30, with higher values indicating worse mental health status.

#### Life satisfaction

The scale ranges from 1 for Not at all satisfied to 5 for Completely satisfied. The variable is recoded dichotomously into not satisfied (0 = not at all satisfied or not very satisfied) and satisfied (1 = somewhat satisfied or very satisfied or completely satisfied) [42].

### Health behaviors

We also investigate the effect of retirement on health behaviors, including physical activity, drinking, and smoking, to explore the mechanism of the effect of retirement on health. Respondents are asked whether they did any physical activity for at least 10 min every week, with 0 indicating no and 1 indicating yes. Drinking and smoking are measured in the same way. Respondents are asked whether they drank or smoked during the last year.

### Control variables

Several sociodemographic variables are controlled in the analysis, including gender, education, marital status, and Hukou status. Education is categorized as low educated (less than lower secondary) and high educated (upper secondary, vocational training, or tertiary). Marital status is categorized as married, partnered, separated, divorced, widowed, or never married. Hukou status is categorized as rural or urban.

### Study design

We use a nonparametric fuzzy regression discontinuity (RD) design to explore the effect of retirement on physical and mental health. The analysis of fuzzy RD design is based on a continuity-based approach in this study. In parametric RD design, the regression function of health on retirement is given in advance by the researcher based on experience or the characteristics of the research problem, which may introduce potential bias if the parametric function form is incorrect. The nonparametric method can relax parametric assumptions and more flexibly approximate the unknown regression function by using data within the bandwidth *h*. However, because all the estimations are based on bandwidth *h*, the sensitivity of results to bandwidth choices must be explored [43, 44].

We use a binary variable $D_i\in\left\{0,1\right\}$ to denote whether a respondent retired or not, where $D_i$ is determined partially or completely based on the value of the running variable $X_i$, age. We use another binary variable $T_i=1\left(X_i\geq c\right)$ to denote the assignment rule, which assigns all respondents whose age is below the cutoff *c* to the employee group, and all respondents whose age is above the cutoff to the retirement group. In the fuzzy RD design, there are some units for which $T_i\neq D_i$. That is, imperfect compliance exists, which is in line with reality. In this analysis, we are more interested in exploring the effect of $D_i$ on health.

Because $D_i$ is determined partially or completely based on the value of the running variable $X_i$, age, the probability of being retired conditional on age, that is, $\Pr\left(D_i=1\vert X_i=c\right)$ is discontinuous at the cutoff, ${X_i} = c$. To estimate the causal effect of retirement on health, we take the ratio of the jump in the conditional mean of the health outcome ${Y_i}$ at the cutoff age to the jump in the conditional treatment probability of being retired, $\Pr ({D_i} = 1|{X_i} = c)$, at the cutoff age, as shown in Formula (1):

1 $${\hat \tau_{_{FRD}}} = \tfrac{{{{\lim }_{x \downarrow c}}\hat E[{Y_i}|{X_i} = x] - {{\lim }_{x \uparrow c}}\hat E[{Y_i}|{X_i} = x]}}{{{{\lim }_{x \downarrow c}}\hat E[{D_i}|{X_i} = x] - {{\lim }_{x \uparrow c}}\hat E[{D_i}|{X_i} = x]}}$$

where ${\hat \tau_{_{FRD}}}$ is the local average treatment effect, indicating the effect of retirement on health around the cutoff, which means that it is not informative about treatment effects at other levels of age.

The validity of the fuzzy RD design requires two standard assumptions [45]. The first assumption requires a discontinuity in the probability of receiving retirement at the cutoff point of age:

$${\lim\nolimits_{x \downarrow c}}\hat E[{D_i}|{X_i} = x] \ne {\lim_{x \uparrow c}}\hat E[{D_i}|{X_i} = x]$$

**Fig. 1:**
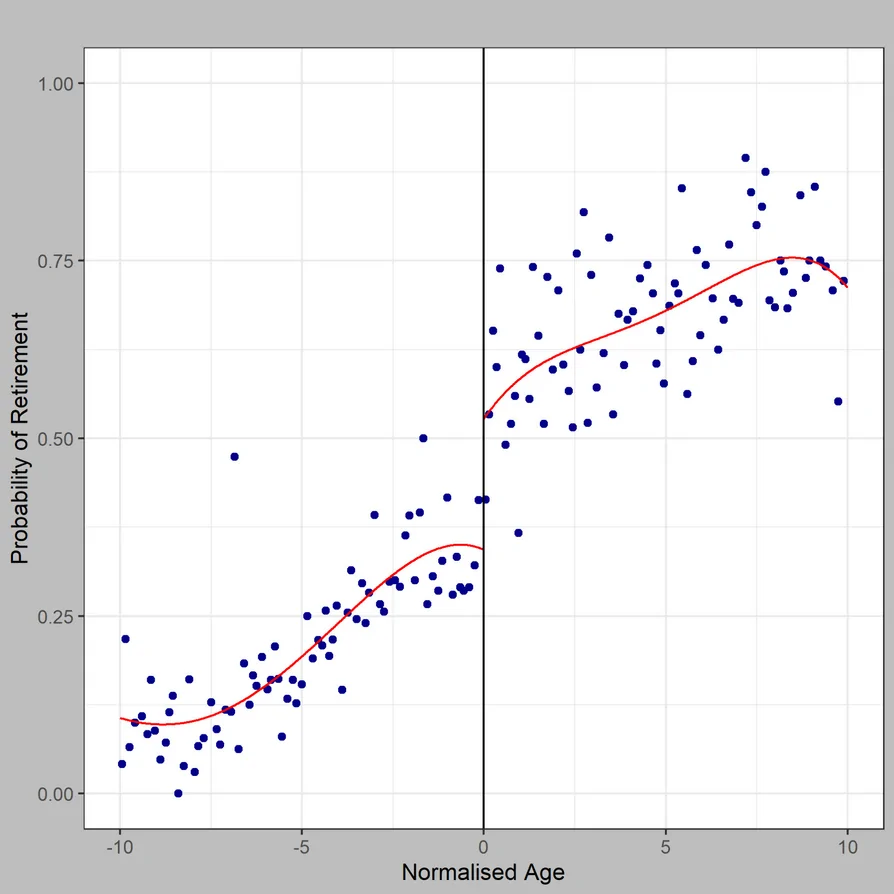
Retirement rate by normalized age. *Note*: We used the mimicking variance quantile-spaced method to estimate the sample means

The assumption is verified graphically in Fig. 1, where the probability of retirement increases sharply at the cutoff. Because males and females have different SRA, the running variable age is normalized by minus the corresponding SRA for each gender from the actual age. We use 55 as SRA for females, because there is a noticeable jump in the probability of retirement at 55 instead of 50 (Additional file 1: Fig. S1 and Table S2). The second assumption requires, at a minimum, that conditional on ${D_i} = d$, there is no discontinuity in the regression functions at the cutoff. That is, that $E[{Y_i}({T_i},0)|{X_i} = x]$ and $E[{Y_i}({T_i},1)|{X_i} = x]$ are continuous in x at c, ensuring that the effect of the treatment assignment at the cutoff is entirely driven by the treatment received, where ${Y_i}({T_i},0)$ and ${Y_i}({T_i},1)$ are potential health outcome variables when individual *i* is treated and untreated, respectively. Naturally, the continuity assumptions that guarantee the validity of the RD design are about unobservable features and as such are inherently untestable. Nonetheless, the RD design offers an array of empirical methods that can provide useful evidence about the plausibility of its assumptions [46]. We perform these validation tests in the latter half of this article.

We follow the practical guide for RD design by Calonico et al. to obtain estimates and perform robustness checks [46, 47]. We use local linear regression at the SRA to obtain nonparametric estimates in Formula (1). In the robustness check section, we explore the sensitivity of higher-order polynomials. We use triangular kernel weights that decrease smoothly as the distance to SRA increases. It leads to a point estimator with optimal properties when used in conjunction with a bandwidth that optimizes the mean squared error (MSE).

We use data-driven bandwidth selectors to address the poor finite sample performance of classical bandwidth selectors. We calculate two types of bandwidths: one is the MSE-optimal bandwidth, and the other is the CER-optimal bandwidth. The MSE-optimal bandwidth results in a point estimator that is not only consistent but also has minimal asymptotic MSE while the CER-optimal bandwidth minimizes an approximation of the coverage error of the confidence interval. We report two types of nonparametric RD estimates for each bandwidth: the conventional RD estimates with a conventional variance estimator (conventional inference) and robust RD estimates with a robust variance estimator (robust inference).

### Discontinuity in retirement

**Fig. 2:**
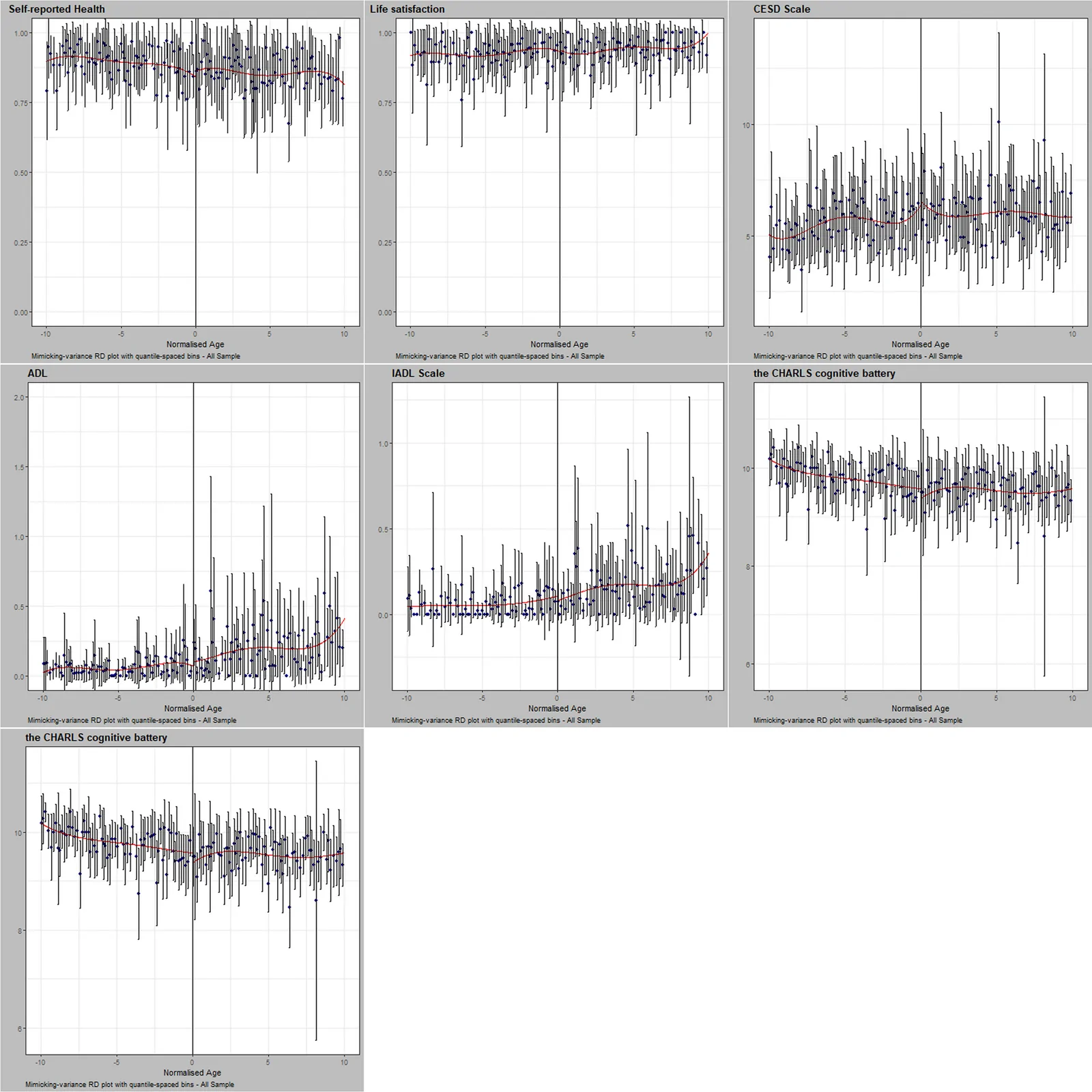
Health outcome discontinuities

We use the RD plot to inspect whether there is a discontinuous change in the probability of retirement at the SRA. Figure 1 shows that there is a jump in the probability of retirement at the SRA, which satisfies the assumption of RD design. We then use RD plots to visually examine the discontinuity of health outcomes at the SRA (Fig. 2). The plot shows the discontinuity of health outcomes at the cutoff age. It also reveals that there is no significant change in all the health outcomes after retirement. The typical RD plot presents two summaries: (i) a global polynomial fit, represented by a solid line, and (ii) local sample means, represented by dots. The global polynomial fit is a smooth approximation to the unknown regression functions based on a fourth-order polynomial regression of the health outcome on age using the original raw data. The local sample means are created by choosing disjoint intervals or bins of age, calculating the mean of the outcome for the observations falling within each bin, and then plotting the average outcome in each bin against the midpoint of the bin. No intersection of the estimated 95% confidence intervals means discontinuity, and vice versa.

Two types of bins can be used in the construction of RD plots: evenly-spaced and quantile-spaced bins. We use quantile-spaced bins because it provides a quick visual representation of the density of observations over the support of the running variable. We use evenly-spaced bins for robustness check (Additional file 1: Figs. S2–S8). We choose the optimal number of bins based on the mimicking variance (MV) approach rather than the integrated mean squared error (IMSE) approach in the main analysis, where the MV approach leads to a larger number of bins than the IMSE approach, resulting in an RD plot giving a better sense of the variability of the data. We use IMSE-optimal bins as a robust check (Additional file 1: Figs. S2–S8).

A complete case analysis was performed because missing rates were low for gender (0%), education (0%), marital status (0.0015%), and Hukou status (5.85%). The proportion of missing values in outcomes varies, from 1.25% (self-reported health) to 40% (physical activity). The complete case analysis was still used because it is not recommended to fill in missing values when the proportion of missing data is too large (40%) and when the dependent variables have missing values [48]. All the statistical analyses are conducted in R (Foundation for Statistical Computing, Vienna, Austria, Version 4.3.1). Package “rdrobust” and “rddensity” are used for fuzzy RD regression analysis.

## Results

### Effects of retirement on health

Tables 1 and 2 shows the RD non-parametric estimates for the effects of retirement on different physical and mental health outcomes. We report results using two types of optimal bandwidths: one based on the MSE-optimal bandwidth selector, and the other based on the CER-optimal bandwidth selector. In the first three columns, we report results when the local linear polynomial fit includes only the running variable as a regressor. Then we generalize the method to accommodate control variables and report the results in the last three columns. As shown in Tables 1 and 2, none of the health outcomes shows a significant change under the given optimal bandwidth, no matter whether the control variables are adjusted. The conventional and robust estimates give similar results. It indicates that retirement has a non-significant effect on the health of Chinese elderly people. The results for all the outcomes are robust for different bandwidths, except for the CHARLS cognitive battery adapted from HRS-HCAP and ADL, where these two health measures show significant changes when the bandwidth is small (Additional file 1: Tables S3–S9 and Figs. S9–S15).

**Table 1:** Nonparametric fuzzy regression discontinuity (RD) estimates

	Without covariates			With covariates		
	Self-reported health	Life satisfaction	CESD scale	Self-reported health	Life satisfaction	CESD scale
MSE Optimal						
Bandwidth	3.3	3.8	3.6	3.5	3.0	3.2
Conventional Point Estimate	0.0811	-0.0567	1.4545	0.1018	-0.0475	1.4448
Conventional 95% CI	[-0.2831, 0.4453]	[-0.3030, 0.1897]	[-3.7613, 6.6703]	[-0.3249, 0.5284]	[-0.3736, 0.2785]	[-5.0521, 7.9418]
Robust Point Estimate	0.0756	-0.0215	1.2355	0.0913	0.0144	1.0375
Robust 95% CI	[-0.3579, 0.5090]	[-0.3100, 0.2671]	[-4.8459, 7.3168]	[-0.4193, 0.6018]	[-0.3761, 0.4049]	[-6.6529, 8.7279]
Effective obs L|R	994|992	1063|1055	1035|1042	1019|1015	867|882	891|907
CER Optimal						
Bandwidth	2.1	2.5	2.4	2.2	2	2.1
Conventional Point Estimate	0.1856	-0.0208	1.9086	0.2071	-0.0073	1.785
Conventional 95% CI	[-0.3283, 0.6995]	[-0.3218, 0.2801]	[-4.8901, 8.7072]	[-0.3798, 0.7939]	[-0.3974, 0.3828]	[-6.4640, 10.0339]
Robust Point Estimate	0.1827	-0.0063	1.7721	0.2025	0.0171	1.5985
Robust 95% CI	[-0.3664, 0.7319]	[-0.3271, 0.3145]	[-5.4279, 8.9721]	[-0.4266, 0.8316]	[-0.4022, 0.4365]	[-7.1938, 10.3908]
Effective obs L|R	646|616	724|695	700|665	698|650	567|562	602|586
Obs L|R	3001|2802	2868|2747	2892|2751	3001|2802	2868|2747	2892|2751

(i) All estimates are computed using a triangular Kernel and nearest neighbor heteroskedasticity-robust variance estimator as suggested by CCT framework. (ii) Effective number of observations depends on the size of the data-driven selected bandwidth

All the *p*-values > 0.05

**Table 2:** Nonparametric fuzzy regression discontinuity (RD) estimates

	Without covariates				With covariates			
	the CHARLS cognitive battery	ADL	IADL	Total Recall Score	the CHARLS cognitive battery	ADL	IADL	Total recall score
MSE Optimal								
Bandwidth	3.6	4.0	2.8	3.9	3.5	3.2	4.1	3.4
Point Estimate	-0.4198	0.0404	-0.1869	2.2990	-0.5379	0.0601	-0.0852	1.4656
Conventional 95% CI	[-2.0137, 1.174]	[-0.3660, 0.4469]	[-0.6746, 0.3009]	[-1.1485, 5.7464]	[-2.3773, 1.3014]	[-0.4417, 0.5619]	[-0.5434, 0.3731]	[-2.6275, 5.5586]
Robust Point Estimate	-0.4843	0.0425	-0.2771	3.0466	-0.6752	0.0600	-0.1155	1.9259
Robust 95% CI	[-2.3391, 1.3704]	[-0.4302, 0.5152]	[-0.8337, 0.2795]	[-1.0749, 7.1681]	[-2.8381, 1.4878]	[-0.5203, 0.6403]	[-0.6489, 0.4179]	[-2.9926, 6.8443]
Effective obs L|R	953|952	1274|1248	778|806	1098|1104	953|952	1035|1003	1146|1217	934|938
CER Optimal								
Bandwidth	2.3	2.6	1.8	2.6	2.3	2.1	2.6	2.2
Point Estimate	-1.1555	0.0534	-0.3394	2.7662	-1.4773	0.2240	-0.2310	1.8908
Conventional 95% CI	[-3.2495, 0.9386]	[-0.3880, 0.4948]	[-0.9972, 0.3183]	[-1.4922, 7.0245]	[-3.8748, 0.9201]	[-0.3397, 0.7877]	[-0.7984, 0.3365]	[-3.0897, 6.8713]
Robust Point Estimate	-1.165	0.0549	-0.3789	3.0688	-1.5217	0.2248	-0.2439	2.0632
Robust 95% CI	[-3.3811, 1.0511]	[-0.4210, 0.5309]	[-1.0807, 0.3230]	[-1.5006, 7.6381]	[-4.0704, 1.0270]	[-0.3844, 0.8340]	[-0.8533, 0.3656]	[-3.2850, 7.4114]
Effective obs L|R	659|619	845|796	541|573	727|691	659|619	664|623	752|773	609|582
Obs L|R	2733|2541	3122|2923	2720|2872	2803|2657	2733|2541	3122|2923	2720|2872	2803|2657

(i) All estimates are computed using a triangular Kernel and nearest neighbor heteroskedasticity-robust variance estimator as suggested by CCT framework. (ii) Effective number of observations depends on the size of the data-driven selected bandwidth

All the *p*-values > 0.05

### Heterogeneity across gender

We explore whether the effects of retirement on health vary across gender. Although retirement poses insignificant impacts on health in both the male and female groups, the effects appeared to go in opposite direction in males and females (Tables 3 and 4, Additional file 1: Figs. S16–S22). Retired men tend to report a better overall health condition, both physically and psychologically. Retired males experience an increase in self-reported health and life satisfaction score, and a decrease in CESD, ADL, and IADL score. On the contrary, retired females experience a decrease in self-reported health and life satisfaction score, and an increase in CESD, ADL, and IADL score. It indicates that retirement has a positive effect on males’ physical and psychological health, while a negative effect on females. However, retirement has a positive effect on females’ cognitive function and a negative effect on males’ cognitive function. Retired females experience an increase of 2.33 points in the CHARLS cognitive battery adapted from HRS-HCAP and 8.83 points in total recall score, while retired males experience a reduction of 1.91 points in the CHARLS cognitive battery adapted from HRS-HCAP and 1.98 points in total recall score. There is stability in the effect of retirement on self-reported health, life satisfaction, the CHARLS cognitive battery adapted from HRS-HCAP, and total recall score at different bandwidths for both males and females (Additional file 1: Tables S10–S16 and Figs. S23–S29). However, results in the CESD scale, ADL, and IADL are somehow sensitive to bandwidth selection. In brief, we conclude that there is gender heterogeneity in the impact of retirement on health outcomes.

**Table 3:** Nonparametric fuzzy regression discontinuity (RD) estimates—gender heterogeneity

	Self-reported health		Life satisfaction		CESD scale	
	Male	Female	Male	Female	Male	Female
MSE Optimal						
Bandwidth	3.2	3.6	2.9	3.8	2.6	3.9
Conventional Point Estimate	0.3551	-0.3737	0.2127	-0.4074	-0.0588	0.5449
Conventional 95% CI	[-0.2034, 0.9137]	[-1.406, 0.6585]	[-0.1408, 0.5663]	[-1.2016, 0.3867]	[-7.3082, 7.1906]	[-13.4954, 14.5853]
Robust Point Estimate	0.4246	-0.5186	0.2917	-0.3544	-0.8639	1.0986
Robust 95% CI	[-0.2357, 1.0849]	[-1.7377, 0.7004]	[-0.1159, 0.6993]	[-1.3216, 0.6128]	[-9.2719, 7.5442]	[-15.8644, 18.0615]
Effective obs L|R	545|528	449|458	487|470	476|475	456|426	484|491
CER Optimal						
Bandwidth	2.1	2.4	1.9	2.6	1.8	2.7
Conventional Point Estimate	0.5860	-0.6615	0.2209	-0.5006	-1.5189	4.0497
Conventional 95% CI	[-0.1914, 1.3634]	[-2.2352, 0.9121]	[-0.1498, 0.5916]	[-1.5536, 0.5524]	[-9.9193, 6.8815]	[-14.5959, 22.6952]
Robust Point Estimate	0.6110	-0.7276	0.2503	-0.4732	-1.842	4.1957
Robust 95% CI	[-0.2223, 1.4444]	[-2.4217, 0.9665]	[-0.1492, 0.6498]	[-1.6146, 0.6681]	[-10.7285, 7.0446]	[-16.0297, 24.4211]
Effective obs L|R	394|345	303|310	339|314	302|308	304|269	315|322
Obs L|R	1709|1610	1292|1192	1626|1584	1242|1163	1636|1583	1256|1168

(i) All estimates are computed using a triangular Kernel and nearest neighbor heteroskedasticity-robust variance estimator as suggested by CCT framework. (ii) Effective number of observations depends on the size of the data-driven selected bandwidth

All the *p*-values > 0.05

**Table 4:** Nonparametric fuzzy regression discontinuity (RD) estimates—gender heterogeneity

	the CHARLS cognitive battery		ADL		IADL			Total Recall Score	
	Male	Female	Male	Female	Male		Female	Male	Female
MSE Optimal									
Bandwidth	3.1	3.7	2.9	3.1	2.2	3.1		3	3
Conventional Point Estimate	-1.9145	2.3318	-0.0176	0.2491	-0.7408	0.3909		-1.9779	8.8329
Conventional 95% CI	[-4.4509, 0.6220]	[-2.8742, 7.5378]	[-0.6954, 0.6601]	[-0.5570, 1.0551]	[-1.7973, 0.3158]	[-0.5211, 1.3028]		[-7.3309, 3.3751]	[-5.1022, 22.768]
Robust Point Estimate	-2.0148	2.1443	-0.0902	0.3111	-0.9604	0.5148		-1.6777	9.8741
Robust 95% CI	[-4.9348, 0.9052]	[-4.0769, 8.3654]	[-0.851, 0.6706]	[-0.6532, 1.2754]	[-2.1403, 0.2195]	[-0.5432, 1.5728]		[-7.9675, 4.612]	[-6.9727, 26.7210]
Effective obs L|R	494|486	424|422	535|500	414|412	356|346	382|391		485|467	341|359
CER Optimal									
Bandwidth	2.1	2.5	1.9	2.1	1.4	2.1		2	2
Conventional Point Estimate	-2.5612	1.245	0.0408	0.8143	-0.6328	0.5464		-0.5967	9.0667
Conventional 95% CI	[-5.7264, 0.6039]	[-3.8770, 6.3670]	[-0.6157, 0.6973]	[-0.9944, 2.6229]	[-1.5267, 0.2612]	[-0.7560, 1.8488]		[-6.0474, 4.8540]	[-8.5039, 26.6373]
Robust Point Estimate	-2.586	1.211	0.0117	0.8529	-0.7135	0.6038		-0.4623	9.5349
Robust 95% CI	[-5.9322, 0.7602]	[-4.3294, 6.7514]	[-0.6937, 0.7171]	[-1.0747, 2.7805]	[-1.6615, 0.2346]	[-0.7824, 1.9900]		[-6.2896, 5.3650]	[-9.5101, 28.5799]
Effective obs L|R	346|304	280|290	373|333	276|285	253|239	251|268		333|300	232|250
Obs L|R	1557|1484	1176|1057	1820|1672	1302|1251	1554|1651	1166|1221		1587|1533	1216|1124

(i) All estimates are computed using a triangular Kernel and nearest neighbor heteroskedasticity-robust variance estimator as suggested by CCT framework. (ii) Effective number of observations depends on the size of the data-driven selected bandwidth

All the *p*-values > 0.05

### Heterogeneity across education

We also explore the heterogeneity regarding education levels of respondents (Tables 5 and 6, and Additional file 1: Figs. S30–S36). Overall, neither the low-educated group nor the high-educated group has a significant change in all the health outcomes after retirement. The RD nonparametric estimates are very close in the two groups for outcomes including self-reported health, life satisfaction, ADL, IADL, and total recall score, except for the the CHARLS cognitive battery adapted from HRS-HCAP and CESD score. Retirees in high-educated group experience a drop of 0.72 in the CESD scale and an increase of 1.39 in the CHARLS cognitive battery adapted from HRS-HCAP. However, the CESD score and the CHARLS cognitive battery adapted from HRS-HCAP of retirees in low-educated group move in the opposite direction. Retirees in low-educated group have an increase of 4.61 in the CESD scale and a decline of 3.28 in the CHARLS cognitive battery adapted from HRS-HCAP. It reveals that high-educated people have fewer depressive symptoms and better cognitive function after retirement. For low-educated people, they have more depressive symptoms and worse cognitive function after retirement. Again, we check the robustness of our results with respect to different bandwidths. The results in self-reported health and total recall score are stable (Additional file 1: Tables S17–S23 and Figs. S37–S43). However, results in life satisfaction, CESD scale, the CHARLS cognitive battery adapted from HRS-HCAP, ADL, and IADL are sensitive to bandwidth selection.

**Table 5:** Nonparametric fuzzy regression discontinuity (RD) estimates—education heterogeneity

	Self-reported health		Life satisfaction		CESD scale	
	Low educated	High educated	Low educated	High educated	Low educated	High educated
MSE Optimal						
Bandwidth	2.6	3.8	3	2.7	3.1	3.3
Conventional Point Estimate	0.0866	0.1072	0.0211	-0.1519	4.6093	-0.7161
Conventional 95% CI	[-0.5828, 0.756]	[-0.3637, 0.5780]	[-0.3572, 0.3993]	[-0.5738, 0.2701]	[-4.6402, 13.8588]	[-7.7306, 6.2984]
Robust Point Estimate	0.0527	0.1343	0.1302	-0.1776	5.7265	-1.9297
Robust 95% CI	[-0.7494, 0.8548]	[-0.4333, 0.7019]	[-0.3128, 0.5733]	[-0.6840, 0.3289]	[-5.4867, 16.9396]	[-10.1049, 6.2455]
Effective obs L|R	394|437	573|419	422|525	377|296	434|543	480|365
CER Optimal						
Bandwidth	1.7	2.6	2	1.8	2	2.3
Conventional Point Estimate	-0.0584	0.1973	0.2163	-0.2383	6.5837	-1.5142
Conventional 95% CI	[-1.0249, 0.908]	[-0.3613, 0.7559]	[-0.3146, 0.7473]	[-0.8462, 0.3696]	[-7.6886, 20.8559]	[-9.8794, 6.851]
Robust Point Estimate	-0.0853	0.2070	0.2675	-0.2484	7.0552	-2.0292
Robust 95% CI	[-1.1280, 0.9574]	[-0.3988, 0.8127]	[-0.3055, 0.8405]	[-0.9018, 0.4050]	[-8.3616, 22.4719]	[-10.9561, 6.8977]
Effective obs L|R	281|306	388|307	297|331	257|225	316|347	333|255
Obs L|R	1407|1938	1594|864	1339|1905	1529|842	1355|1909	1537|842

(i) All estimates are computed using a triangular Kernel and nearest neighbor heteroskedasticity-robust variance estimator as suggested by CCT framework. (ii) Effective number of observations depends on the size of the data-driven selected bandwidth

All the *p*-values > 0.05

**Table 6:** Nonparametric fuzzy regression discontinuity (RD) estimates—education heterogeneity

	the CHARLS cognitive battery		ADL		IADL		Total Recall Score	
	Low educated	High educated	Low educated	High educated	Low educated	High educated	Low educated	High educated
MSE Optimal								
Bandwidth	2.9	2.6	3.7	4.1	2.6	3.7	2.7	2.6
Point Estimate	-3.2857	1.3855	0.0857	0.0213	-0.0460	-0.2805	2.1210	2.5760
Conventional 95% CI	[-7.0057, 0.4344]	[-0.9219, 3.6929]	[-0.3660, 0.5373]	[-0.6758, 0.7184]	[-0.7264, 0.6344]	[-0.9828, 0.4218]	[-4.0528, 8.2947]	[-1.9729, 7.1248]
Robust Point Estimate	-3.8694	1.2775	0.0917	0.0141	-0.0460	-0.3556	2.4466	2.6951
Robust 95% CI	[-8.3325, 0.5937]	[-1.4901, 4.045]	[-0.4219, 0.6053]	[-0.8168, 0.8449]	[-0.9154, 0.6873]	[-1.1730, 0.4618]	[-4.9211, 9.8142]	[-2.7263, 8.1164]
Effective obs L|R	388|455	366|291	561|687	653|470	349|432	561|423	388|447	376|290
CER Optimal								
Bandwidth	1.9	1.8	2.5	2.8	1.7	2.5	1.8	1.8
Point Estimate	-4.8316	0.7453	0.2397	-0.0471	-0.2286	-0.3634	1.9875	2.4152
Conventional 95% CI	[-11.2327, 1.5695]	[-1.9656, 3.4563]	[-0.3093, 0.7888]	[-0.7791, 0.6848]	[-1.1782, 0.7211]	[-1.1364, 0.4095]	[-5.8243, 9.7993]	[-3.5042, 8.3347]
Robust Point Estimate	-5.1041	0.7125	0.2409	-0.0492	-0.2286	-0.3948	2.1492	2.4708
Robust 95% CI	[-12.0004, 1.7922]	[-2.2071, 3.6320]	[-0.3573, 0.8392]	[-0.8453, 0.7469]	[-1.3160, 0.7705]	[-1.2275, 0.4379]	[-6.2717, 10.5702]	[-3.9067, 8.8483]
Effective obs L|R	272|297	232|198	403|439	434|337	250|306	379|309	276|312	237|200
Obs L|R	1241|1720	1492|821	1454|2012	1668|911	1251|1968	1469|904	1294|1823	1509|834

(i) All estimates are computed using a triangular Kernel and nearest neighbor heteroskedasticity-robust variance estimator as suggested by CCT framework. (ii) Effective number of observations depends on the size of the data-driven selected bandwidth

All the *p*-values > 0.05

### Effects of retirement on health behaviors

We investigate the effects of retirement on health behaviors (including smoking, drinking, and physical activities) to explore the mechanism of the effects of retirement on health. Table 7 shows that retirement has an insignificant impact on the behaviors of respondents. That is, the lifestyles and behavior patterns of respondents do not change significantly after retirement, which may be the reason why the effects on health are insignificant. We also assess heterogeneity across gender and education. Both males and females smoke more after retirement (Additional file 1: Table S24). However, females drink less and have more physical activities while males drink more and have fewer physical activities. Both high- and low-educated groups have fewer physical activities after retirement (Additional file 1: Table S25). However, compared with the low-educated group, the high-educated group tended to smoke and drink less.

**Table 7:** Nonparametric fuzzy regression discontinuity (RD) estimates

	Without covariates			With covariates		
	Smoking	Drinking	Physical Activity	Smoking	Drinking	Physical Activity
MSE Optimal						
Bandwidth	3.5	3.6	3.1	3.6	3.5	3.5
Point Estimate	-0.0434	-0.0901	-0.0596	0.2871	0.1798	-0.0702
Conventional 95% CI	[-0.4578, 0.3710]	[-0.5639, 0.3836]	[-0.2505, 0.1313]	[-0.1567, 0.7309]	[-0.3496, 0.7092]	[-0.2849, 0.1444]
Robust Point Estimate	-0.0597	-0.0953	-0.0563	0.3298	0.2192	-0.0902
Robust 95% CI	[-0.5481, 0.4287]	[-0.6566, 0.4660]	[-0.2773, 0.1647]	[-0.1914, 0.8509]	[-0.4096, 0.8479]	[-0.3367, 0.1564]
Effective obs L|R	1032|1001	1140|1109	583|596	1032|1001	1087|1057	667|689
CER Optimal						
Bandwidth	2.3	2.3	2	2.3	2.2	2.3
Point Estimate	-0.0883	-0.1436	-0.0370	0.1776	0.0844	-0.0536
Conventional 95% CI	[-0.6237, 0.4471]	[-0.7612, 0.4740]	[-0.2547, 0.1807]	[-0.3730, 0.7283]	[-0.5904, 0.7593]	[-0.2884, 0.1812]
Robust Point Estimate	-0.0931	-0.1435	-0.0370	0.1965	0.1012	-0.0634
Robust 95% CI	[-0.6636, 0.4774]	[-0.8022, 0.5152]	[-0.2691, 0.1950]	[-0.3886, 0.7817]	[-0.6199, 0.8223]	[-0.3127, 0.1860]
Effective obs L|R	721|653	769|708	406|402	721|653	743|678	476|446
Obs L|R	2998|2700	3191|2924	1806|1866	2998|2700	3191|2924	1806|1866

(i) All estimates are computed using a triangular Kernel and nearest neighbor heteroskedasticity-robust variance estimator as suggested by CCT framework. (ii) Effective number of observations depend on the size of the data-driven selected bandwidth

All the *p*-values > 0.05

### Robustness checks

We check the robustness of the results regarding different bandwidths, kernel functions, and polynomial functions.

The bandwidth *h* controls the width of the neighborhood around the cutoff that is used to fit the local polynomial that approximates the unknown regression function. Choosing a smaller bandwidth will reduce the misspecification error but will simultaneously increase the variance. In contrast, a larger bandwidth will result in higher misspecification error but lower variance. Therefore, the fuzzy RD estimates at different bandwidths could help investigate whether the nonparametric RD estimates of retirement are sensitive to bandwidth selection. Our analysis indicates that the RD estimates of the retirement effect from the MSE-optimal bandwidth selector are consistent with the results from the CER-optimal bandwidth (Tables 1 and 2). Also, most of the RD estimates are stable with respect to different bandwidths and consistent with the baseline estimates (Additional file 1: Tables S3–S9 and Figs. S9–S15).

Choosing kernel function is another challenge in RD design. The kernel function assigns non-negative weights to each observation, based on the distance between the observation and the cutoff. There are three commonly used kernel functions: triangular kernel, uniform kernel, and Epanechnikov kernel. In practice, estimation and inference results are typically not very sensitive to the particular choice of kernel used [46]. Nevertheless, we use the above-mentioned three kernel functions to check the robustness of the RD estimates. The results in Table 8 indicate that estimates remain stable for all outcomes across the different kernel functions.

**Table 8:** Nonparametric fuzzy regression discontinuity (RD) estimates for different kernel functions and polynomial orders

	Linear		Quadratic		
	Uniform	Epanechnikov	Triangular	Uniform	Epanechnikov
**Self-reported Health**					
MSE estimate	0.0713	0.1223	0.2073	0.1343	0.1901
MSE 95% CI (Robust)	[-0.4271, 0.6522]	[-0.2614, 0.6626]	[-0.2856, 0.7152]	[-0.3595, 0.6707]	[-0.3174, 0.6993]
CER estimate	0.1908	0.1789	0.3071	0.3366	0.3141
CER 95% CI (Robust)	[-0.2410, 0.6460]	[-0.2556, 0.6721]	[-0.1479, 0.7624]	[-0.1336, 0.8088]	[-0.1583, 0.7839]
**Life Satisfaction**					
MSE estimate	-0.0388	-0.0523	-0.0096	-0.0073	0.0097
MSE 95% CI (Robust)	[-0.2922, 0.3659]	[-0.3056, 0.2627]	[-0.4308, 0.4240]	[-0.4178, 0.4471]	[-0.2526, 0.4002]
CER estimate	0.0012	-0.0208	0.0334	0.1603	0.0200
CER 95% CI (Robust)	[-0.3538, 0.4252]	[-0.3238, 0.3073]	[-0.6207, 0.6912]	[-0.4646, 0.8023]	[-0.3862, 0.4592]
**CESD Scale**					
MSE estimate	1.7267	1.5256	1.1630	0.4545	0.8932
MSE 95% CI (Robust)	[-4.7193, 7.9906]	[-4.6008, 7.2318]	[-6.6795, 8.2401]	[-7.0290, 6.4456]	[-6.7963, 7.2877]
CER estimate	2.8464	1.8994	1.9409	2.4735	1.5403
CER 95% CI (Robust)	[-4.0598, 9.5874]	[-5.1994, 8.7478]	[-9.0435, 12.6964]	[-9.1326, 13.5295]	[-8.7563, 11.4628]
**the CHARLS cognitive battery**					
MSE estimate	-0.3424	-0.3061	-1.7019	-0.4279	-0.3921
MSE 95% CI (Robust)	[-2.1276, 1.9789]	[-2.1753, 1.4746]	[-5.2345, 0.6942]	[-2.7139, 1.9867]	[-2.4645, 1.7147]
CER estimate	-1.5067	-1.1695	-3.2320	-2.7495	-2.0925
CER 95% CI (Robust)	[-3.9406, 1.2202]	[-3.3806, 1.0451]	[-8.5216, 1.6584]	[-6.6328, 1.1822]	[-5.1854, 1.0456]
**ADL**					
MSE estimate	-0.0503	-0.0162	0.1017	-0.0118	0.0094
MSE 95% CI (Robust)	[-0.6303, 0.4657]	[-0.5466, 0.4804]	[-0.4426, 0.7244]	[-0.7176, 0.6824]	[-0.5437, 0.6063]
CER estimate	0.0883	0.0850	0.7578	0.6323	0.5535
CER 95% CI (Robust)	[-0.5406, 0.6817]	[-0.4462, 0.6005]	[-0.3303, 1.8812]	[-0.5150, 1.7795]	[-0.3358, 1.4601]
**IADL**					
MSE estimate	-0.1860	-0.2023	-0.3310	-0.2916	-0.3179
MSE 95% CI (Robust)	[-0.8677, 0.3169]	[-0.8619, 0.2796]	[-1.178, 0.3632]	[-1.1526, 0.3478]	[-1.1766, 0.3118]
CER estimate	-0.4416	-0.3909	-0.2941	-0.4515	-0.3638
CER 95% CI (Robust)	[-1.1999, 0.2409]	[-1.1604, 0.3011]	[-1.2559, 0.6350]	[-1.6777, 0.7101]	[-1.2994, 0.518]
**Total Recall Score**					
MSE estimate	2.3618	2.3359	3.003	3.6381	3.2775
MSE 95% CI (Robust)	[-1.6244, 7.0584]	[-0.9632, 7.2064]	[-3.0597, 8.8719]	[-2.1318, 10.2123]	[-2.6941, 9.1793]
CER estimate	5.0619	2.9857	1.0830	3.3154	1.5234
CER 95% CI (Robust)	[-0.9115, 11.2045]	[-1.1902, 7.7865]	[-6.4441, 8.5652]	[-3.3895, 10.2133]	[-5.5864, 8.6324]

All the* p*-values > 0.05

A more consequential decision is the choice of the local polynomial order. In general, the local linear RD estimator is the standard choice in the RD design. For a given bandwidth, increasing the order of the polynomial generally improves the accuracy of the estimates but also increases the variance. We check the second-order polynomial for the running variable. We find that the RD estimates are close to the first-order estimates, but the 95% confidence intervals are wider.

### Falsification and validation of the regression discontinuity design

RD assumptions are needed to be met to guarantee the validity of RD design. In this section, we perform several validation tests based on (a) the null treatment effect on predetermined covariates, (b) the continuity of the running variable density around the cutoff, (c) the treatment effect at artificial cutoff values, and (d) the exclusion of observations near the cutoff.

We check the assumption that the predetermined variables are continuous at the cutoff, in other words, to check that retirement has no effect on them. The results in Additional file 1: Table S26 and Fig. S44 imply that the continuity assumption is not rejected for all the predetermined variables. That is, we find no evidence that the predetermined variables are discontinuous at the cutoff age.

We then check another assumption that the respondents do not have the ability to precisely manipulate their age. In other words, the number of respondents just above the cutoff should be approximately similar to the number of respondents just below it. That is, age is continuously distributed near the cutoff. We use the manipulation test to check the assumption. Additional file 1: Table S27 indicates no manipulation of the running variable at cutoff. Additional file 1: Fig. 45 shows the density of employees and retirees are very near to each other at the cutoff, and the confidence intervals overlap.

The placebo cutoff test examines whether retirement effects are significant at placebo cutoffs. This test replaces the true cutoff with another value at which the treatment status does not really change and performs estimation and inference using this artificial cutoff point. We set the placebo cutoff at -1 and 1 away on the left and right of the true cutoff. We also reset the female cutoff age to 50 to test the robustness. The p-values are all greater than zero, which means that the outcomes do not jump discontinuously at the placebo cutoffs (Additional file 1: Table S28).

The last falsification approach seeks to investigate how sensitive the results are to the observations close to the cutoff. If manipulation of age exists, it is natural to assume that observations closest to the cutoff are most likely to be engaged. The idea behind this approach is to exclude such observations and then repeat the estimation and inference analysis using the remaining sample, which is referred to as the “donut hole” approach. We set the donut hole radius at 0.1, 0.2, and 0.3. Additional file 1: Table S29 shows that the results of the donut hole test are similar to those of the original analysis. Consequently, the fuzzy RD estimates are robust for excluding some observations around the cutoff.

## Discussion

Our study aims to investigate the effect of retirement on the physical and mental health of elderly people in China. We explore several health outcomes that comprehensively reflect the physical and mental health of elderly people. The results of this study show that retirement has an insignificant effect on the health of the elderly. Moreover, the effect remains insignificant after adjusting control variables. We also investigate whether the effects of retirement on health differ with respect to gender and education, and heterogeneity does exist. We perform robust checks for different bandwidths, kernel functions, and polynomial orders. Most of the results are stable. We try to explore the mechanism for the change in the health status caused by retirement by investigating the lifestyle changes before and after retirement. We investigate three health behaviors, including drinking, smoking, and physical activities. The retirees’ lifestyle and behavior patterns have no significant change compared to the employees. This may be the reason why retirement has minimal effects on health.

We compare our findings to the results of previous studies on Chinese people. The results are consistent with some studies that retirement has an insignificant effect on health [15, 24, 32]. However, our study also shows contradictory results compared with other earlier findings. Xu et al. [18] and Lu et al. [49] found that retirement can increase the depression risk of Chinese employees, while Fang et al. [12] and Peng et al. [33] found that retirement decreases the depression risk and improves self-reported health. Zhang et al.’s findings [13] showed that retirement significantly increased the happiness of men in urban China. Lai et al. [31] found that retirement declined physical health, and posed both negative and positive influences on mental health. Li et al.’s study [17] showed that late retirement was associated with better cognitive status. The contradictory findings may result from several sources, such as different study populations, methodology differences, sample construction, and so on.

First, the study population often differs in different studies. In Zhang et al.’s study, data came from the Chinese General Social Survey (CGSS), and only males aged between 40 and 80 are included [13]. Lai et al. investigated retirees in Shenzhen and Hong Kong in their study, where the sample is not representative of Chinese elderly people [31].

Second, although several studies used the same dataset (CHARLS) as we did, the sample construction process differs. Xu et al.’s study [18] included respondents aged over 45, who were employed at baseline, no matter whether the respondent was covered by the pension plan. The definition of retirement also differs, where a respondent was labeled as retired if he or she was not currently engaged in agricultural or non-agricultural work but has worked for at least 3 months during their lifetime and has not searched for a job during the past month at the time of interview. Li et al.’s study only included respondents in the 2015 and 2018 wave [17]. Peng et al.’s study [33] only used cross-sectional data in the 2018 wave.

Third, the methodological difference may play a critical role in the contradictory results. Previous study has shown that the choice of analysis method is one of the key factors in explaining why the estimated results of the effect of retirement on health differ [36]. Xu et al.’s study used a random effects model. Fang et al.’s study [12] also applied the fuzzy RD design. However, the choice of bandwidth in their study was determined artificially, not driven by data. Lai et al. [31] employed a qualitative design with narrative interviews in their study to investigate how retirement influenced the health of elderly people. Peng et al.’s study [33] applied regression analysis based on propensity score matching with a generalized boosted model.

When compared to global evidence, there are still no unified views on the impact of retirement on various health outcomes. Some studies support that retirement has a positive impact on health [50–52], while some studies conclude that retirement has a negative impact [10, 53]. Others reach the same conclusion as our study that retirement has no effect [54, 55]. The differences in results may be caused by the above-mentioned reasons. It may also be explained by institutional differences, different races, different measures of health outcomes, and so on [36, 38].

Consistent with previous studies, gender, and education introduce heterogeneity to the effect of retirement on health.

Although health indicators for men and women do not change significantly after retirement, the changes are in the opposite direction. Our results show that retirement tends to have a positive effect on ADL and IADL scores, and depression in men, while a negative effect in women. Therefore, it is not surprising that men reported increased life satisfaction and self-reported health, whereas women reported decreased life satisfaction and self-reported health. However, the effect of retirement on cognitive function tends to be negative for males and positive for females. Overall, our results indicate that retirement tends to have a positive effect on males’ physical and psychological health, while a negative effect on females. Therefore, the overall null effects of retirement on health appear to hide opposite gender-specific effects. Our results support some previous research [12, 23, 25, 33] but are contradictory to some others [9, 18, 25].

We try to explore the mechanism that causes heterogeneity by investigating lifestyle changes before and after retirement. We find that heterogeneity in health behaviors exists across gender. Both males and females tend to smoke more, but the change in females is much smaller. Females tend to drink less and have more physical activities while males tend to drink more and have fewer physical activities. The fact that males drink and smoke more may partly explain why males have a decline in cognitive function, as smoking and alcohol consumption increase the risk of cognitive impairment [56, 57]. The improvement in females’ cognitive function may partly result from increased physical activities and reduced alcohol consumption. This is consistent with previous findings that men are at higher risk of cognitive impairment than women [56]. Sex differences in other risk factors like stroke and cardiovascular diseases need to be explored to further explain the mechanism. The reason is not straightforward why men have improved physical health and fewer symptoms of depression while what women experience goes just the opposite. There are several possible explanations. After retirement, males are relieved from work activities and usually do not take housework. Moreover, males have more time for recreational social participation, which would enhance their mental health [58] and may also be the reason that males drink and smoke more after retirement. Thus, they may feel less stressed and depressed, and more satisfied with life. In contrast, females are generally family-centered and still need to do housework and childcare, which may be one reason for the decline in physical and psychological health in females. Physical function, assessed by ADL and IADL, is associate with age, education level, cognitive function, depression, chronic diseases, lifestyles, and so on [59]. We are not able to conclude which factor contributes dominantly to the change of physical function. Further research is needed to assess the magnitude of the effect of each factor.

Our study also reveals that compared to high-educated retirees, low-educated retirees are more likely to have depressive symptoms and cognitive function decline. The result is in line with previous studies conducted on Chinese elderly [12, 18]. Education is documented to increase the efficiency of producing health, and thus result in better health outcomes [38]. Higher-educated retirees have more capacity and choices in maintaining their life patterns, which leads to healthier behaviors and stronger cognitive skills [60]. The result is also consistent with previous findings that depression has been significantly associated with low education levels [61, 62]. The main reason may be that highly educated retirees are more likely to have higher-income positions before retirement, which could translate to certain socio-economic advantages that may improve an individual’s psychological well-being [62]. Moreover, family environment, personal intelligence, or abilities were probably the key factors in deciding whether one could attain a lower or higher education. These mental resources may in turn give higher resilience to strain or stresses, hence protecting against depression and cognitive impairment [61].

Regarding health behaviors, both high- and low-educated groups have fewer physical activities after retirement. However, compared with the low-educated group, the high-educated group tend to smoke and drink less. It may reveal the reason why high-educated people have fewer depressive symptoms and better cognitive function after retirement. The conclusion is in line with previous research, smoking and heavy alcohol consumption are the risk factors for symptoms of depression and cognitive impairment [56, 57, 63–65]. Moreover, depression may further lead to increased consumption of alcohol [63]. Overconsumption of alcohol was not tested in our study due to the large number of missing values. Moreover, there may exist other factors associated with depression and cognition, such as social interaction, family history, and so on. Further research is needed to address the mechanism of how these changes occurred.

Our study has several advantages. First, in our study, we analyze as many health indicators (including physical and mental health indicators) as possible to comprehensively assess the effect of retirement on the health of Chinese elderly people. Our study contributes to the broader literature on the effect of retirement on health. Second, compared with other studies that adopted a fuzzy regression discontinuity design, we use age in months as a running variable rather than age in years, which makes the continuity-based approach more suitable in this situation because the number of mass points is larger. In addition, we perform a series of robustness checks to prove that our results are stable.

The results of our study have important policy implications. Population aging has become a formidable challenge worldwide. It imposes huge costs on public and private budgets and leads to a shortage of working force. Moreover, the mandatory retirement age in China is among the youngest in the world. Therefore, there is a strong economic incentive for China to increase retirement age. The findings of our study suggest that increasing retirement age may reduce the economic burden and have minimal effect on the health of the Chinese elderly people.

Our research has some limitations. First, CHARLS is an observational study. Thus, it is not that reliable to make a causal inference. Second, although several confounding variables are controlled in the analysis, there may be other confounding variables that cannot be controlled due to the data limitation. Potential confounding variables may include family income, dietary patterns, genetic factors, access to health care, and so on. Third, the external validity of the RD design is limited because it estimates the effects based on observations close to the cutoff of the running variable. Thus, our work only captures a short-term effect for a sub-population that qualify the pension program. Fourth, complete case analysis was used in our study, selection bias may exist, and statistical power was reduced due to the reduced sample size. Last, the cutoff point of age in women is driven by data, because we are unable to obtain the information whether a woman retires at 50 or 55. Therefore, the confidence interval of health indicators in women is wide. In consequence, our results should be interpreted with caution.

## Conclusion

Our study explores the short-term effect of retirement on the physical and mental health of Chinese elderly people using a sample drawn from the Harmonized CHARLS dataset. Results suggest that retirement has no significant impact on the health status of elderly people. Therefore, increasing the retirement age can release the economic burden and pose minimal effects on people. Further research is needed to verify the results and to study the long-term health consequences of retirement.

## Supplementary Information

Supplementary Material 1.

## Data Availability

Data is publicly available. See: http://charls.pku.edu.cn/.

## Abbreviations

RD: Regression discontinuity
SRA: Statutory retirement age
CHARLS: China Health and Retirement Longitudinal Study
ADL: Activities of daily living
IADL: Instrumental activities of daily living
HRS: Health and Retirement Study
HCAP: Harmonized Cognitive Assessment Protocol
CESD: The Center for Epidemiologic Studies Depression Scale
MSE: Mean square error
CER: Coverage error
MV: Mimicking variance
IMSE: Integrated mean squared error
