# Correction: The effect of retirement on physical and mental health in China: a nonparametric fuzzy regression discontinuity study

**DOI:** 10.1186/s12889-026-28991-w

**Published:** 2026-09-11

**Authors:** Ting Wang, Huizhen Liu, Xiaoqin Zhou, Changxi Wang

**Affiliations:** 1 Department of Clinical Research Management, Center of Biostatistics, Design, Measurement and Evaluation (CBDME), West China Hospital, Sichuan University, Chengdu, Sichuan China; 2 West China Biomedical Big Data Center, West China Hospital, Sichuan University, Chengdu, Sichuan China; 3 Med‑X Center for Informatics, Sichuan University, Chengdu, China; 4 Sichuan University ‑ Pittsburgh Institute, Sichuan University, Chengdu, 610207 China

**Correction: BMC Public Health 24, 1184 (2024)**

**https://doi.org/10.1186/s12889-024-18649-w**

Following the publication of the original article, the author reported that the instrument used in the article should be referred to as the “CHARLS cognitive battery adapted from Harmonized Cognitive Assessment Protocol designed by the Health and Retirement Study (HRS-HCAP) instead” of “Mini Mental State Exam (MMSE),”. The terminology has been corrected to respect the registered trademark and intellectual property rights.

The original article [1] has been corrected.
