# Supplementary material for: The effect of retirement on physical and mental health in China: a nonparametric fuzzy regression discontinuity study
Source: BMC Public Health. 2024 Apr 27;24:1184. doi: 10.1186/s12889-024-18649-w (PMC11520119; doi:10.1186/s12889-024-18649-w)
Supplement: Supplementary file 1 — Supplementary Material 1. [file 12889_2024_18649_MOESM1_ESM.pdf]

Contents

Table S1. Flowchart of sample selection 4

Table S2. Cutoff age of female employees 5

Table S3. Fuzzy RD estimates at different bandwidths – Self-reported health – All samples at age 65 6

Table S4. Fuzzy RD estimates at different bandwidths – Life satisfaction – All samples at age 65 7

Table S5. Fuzzy RD estimates at different bandwidths – CESD Scale – All samples at age 65 8

Table S6. Fuzzy RD estimates at different bandwidths – MMSE – All samples at age 65 9

Table S7. Fuzzy RD estimates at different bandwidths – ADL – All samples at age 65 10

Table S8. Fuzzy RD estimates at different bandwidths – IADL Scale – All samples at age 65 11

Table S9. Fuzzy RD estimates at different bandwidths – Total Recall Score – All samples at age 65 12

Table S10. Fuzzy RD estimates at different bandwidths – Self-reported health – By gender at age 65 13

Table S11. Fuzzy RD estimates at different bandwidths – Life satisfaction – By gender at age 65 14

Table S12. Fuzzy RD estimates at different bandwidths – CESD Scale – By gender at age 65 15

Table S13. Fuzzy RD estimates at different bandwidths – MMSE – By gender at age 65 16

Table S14. Fuzzy RD estimates at different bandwidths – ADL – By gender at age 65 17

Table S15. Fuzzy RD estimates at different bandwidths – IADL Scale – By gender at age 65 18

Table S16. Fuzzy RD estimates at different bandwidths – Total recall score – By gender at age 65 19

Table S17. Fuzzy RD estimates at different bandwidths – Self-reported health – By education at age 65 20

Table S18. Fuzzy RD estimates at different bandwidths – Life satisfaction – By education at age 65 21

Table S19. Fuzzy RD estimates at different bandwidths – CESD Scale – By education at age 65 22

Table S20. Fuzzy RD estimates at different bandwidths – MMSE – By education at age 65 23

Table S21. Fuzzy RD estimates at different bandwidths – ADL – By education at age 65 24

Table S22. Fuzzy RD estimates at different bandwidths – IADL Scale – By education at age 65 25

Table S23. Fuzzy RD estimates at different bandwidths – Total recall score – By education at age 65 26

Table S24. Nonparametric fuzzy regression discontinuity (RD) estimates—Gender heterogeneity at age 65 27

Table S25. Nonparametric fuzzy regression discontinuity (RD) estimates—Education heterogeneity at age 65 28

Table S26. Formal falsification test for the validity of the Continuity-Based Analysis for predetermined Covariates 29

Table S27. Manipulation test of running variable 30

Table S28. Placebo cutoff 31
Table S29. Fuzzy RD sensitivity test to observations near cutoff: The Donut-Hole Approach 32
Fig. S1 Cutoff age for female employees 33
Fig. S2 Self-reported Health RD Plot 34
Fig. S3 Life Satisfaction RD Plot 35
Fig. S4 CESD Scale RD Plot 36
Fig. S5 MMSE RD Plot 37
Fig. S6 ADL RD Plot 38
Fig. S7 IADL RD Plot 39
Fig. S8 Total Recall Score RD Plot 40
Fig. S9 Nonparametric Regression discontinuity robustness of bandwidth choice for self-reported health 41
Fig. S10 Nonparametric Regression discontinuity robustness of bandwidth choice for life satisfaction 42
Fig. S11 Nonparametric Regression discontinuity robustness of bandwidth choice for CESD Scale 43
Fig. S12 Nonparametric Regression discontinuity robustness of bandwidth choice for MMSE 44
Fig. S13 Nonparametric Regression discontinuity robustness of bandwidth choice for ADL 45
Fig. S14 Nonparametric Regression discontinuity robustness of bandwidth choice for IADL 46
Fig S15 Nonparametric Regression discontinuity robustness of bandwidth choice for total recall score 47
Fig. S16 Self-reported Health RD Plot by Gender 48
Fig. S17 Life Satisfaction RD Plot by Gender 49
Fig. S18 CESD Scale RD Plot by Gender 50
Fig. S19 MMSE RD Plot by Gender 51
Fig. S20 ADL RD Plot by Gender 52
Fig. S21 IADL RD Plot by Gender 53
Fig. S22 Total Recall Score RD Plot by Gender 54
Fig. S23 Nonparametric Regression discontinuity robustness of bandwidth choice for self-reported health by gender 55
Fig. S24 Nonparametric Regression discontinuity robustness of bandwidth choice for life satisfaction by gender 56
Fig. S25 Nonparametric Regression discontinuity robustness of bandwidth choice for CESD scale by gender 57
Fig. S26 Nonparametric Regression discontinuity robustness of bandwidth choice for MMSE by gender 58

Fig. S27 Nonparametric Regression discontinuity robustness of bandwidth choice for ADL by gender 59
Fig. S28 Nonparametric Regression discontinuity robustness of bandwidth choice for IADL by gender 60
Fig. S29 Nonparametric Regression discontinuity robustness of bandwidth choice for Total recall score by gender 61
Fig. S30 Self-reported Health RD Plot by Education 62
Fig. S31 Life Satisfaction RD Plot by Education 63
Fig. S32 CESD Scale RD Plot by Education 64
Fig. S33 MMSE RD Plot by Education 65
Fig. S34 ADL RD Plot by Education 66
Fig. S35 IADL RD Plot by Education 67
Fig. S36 Total Recall Score RD Plot by Education 68
Fig. S37 Nonparametric Regression discontinuity robustness of bandwidth choice for self-reported health by education 69
Fig. S38 Nonparametric Regression discontinuity robustness of bandwidth choice for life satisfaction by education 70
Fig. S39 Nonparametric Regression discontinuity robustness of bandwidth choice for CESD scale by education 71
Fig. S40 Nonparametric Regression discontinuity robustness of bandwidth choice for MMSE by education 72
Fig. S41 Nonparametric Regression discontinuity robustness of bandwidth choice for ADL by education 73
Fig. S42 Nonparametric Regression discontinuity robustness of bandwidth choice for IADL by education 74
Fig. S43 Nonparametric Regression discontinuity robustness of bandwidth choice for Total recall score by education 75
Fig. S44 The predetermined covariates by normalized age 76
Fig. S45 Running variable density test 77

Table S1. Flowchart of sample selection

	Person-waves	Individuals
Total sample size in four waves	102,344	25,586
Keep Participants working in government/ have basic pension plans with firms	10,855	5,825
Keep males aged 50 - 70, and females aged 45 – 65	6,965	4,039
Drop participants never worked or employed	6,888	4,010
Drop participants retired due to health reasons	6,678	3,913
Drop participants who did not report "retired or not"	6,503	3,837
Drop missing values in marital status	6,502	3,837
Drop missing values in the Hukou status	6,122	3,564
Drop missing values in the outcome variable		
Self-reported health	5,803	3,405
Life satisfaction	5,615	3,309
CESD scale	5,643	3,318
ADL	6,045	3,532
IADL	5,592	3,380
Total recall score	5,460	3,222
MMSE	5,274	3,316
Physical activity	3,673	2,715
Drinking	6,115	3,562
Smoking	5,698	3,422

Table S2. Cutoff age of female employees

	Cutoff at 55	Cutoff at 50
Optimal bandwidth	2.69	1.01
Conventional estimate	0.1075	0.2612
P-value	0.1598	0.0200**
Conventional 95% CI	[-0.0424, 0.2573]	[0.0411, 0.4813]
Robust estimate	0.1089	0.2759
P-value	0.1887	0.0235**
Robust 95% CI	[-0.0535, 0.2713]	[0.0372, 0.5145]
Obs L|R	1371|1252	670|1953
Effective obs	351|344	155|159

Note: (i) All estimates are computed using a triangular Kernel and local linear regression. (ii) Bandwidth h, for the RD estimator, and bandwidth b for the bias-corrected estimator is data-driven coverage-error optimal for inference. (iii) Effective number of observations depends on the size of the data-driven selected bandwidth.

** indicates a significance level at 5%.

Table S3. Fuzzy RD estimates at different bandwidths – Self-reported health – All samples at age 65

Bandwidths	Conventional Estimate	95% CI (Conventional)	95% CI (Robust)	Obs L|R
3.3	0.0808	[-0.2842, 0.4459]	[-0.3574, 0.7292]	3001|2802
1	-0.2455	[-1.3996, 0.9085]	[-2.2229, 1.3516]	3001|2802
1.5	0.0578	[-0.5857, 0.7012]	[-1.2637, 0.7118]	3001|2802
2	0.1739	[-0.3599, 0.7077]	[-0.8590, 0.7319]	3001|2802
2.5	0.1676	[-0.2778, 0.6130]	[-0.5611, 0.7654]	3001|2802
3	0.0936	[-0.2965, 0.4837]	[-0.3717, 0.7922]	3001|2802
3.5	0.0863	[-0.2684, 0.4410]	[-0.3714, 0.6834]	3001|2802
4	0.0916	[-0.2400, 0.4233]	[-0.3719, 0.6085]	3001|2802
4.5	0.1076	[-0.2155, 0.4307]	[-0.3878, 0.5639]	3001|2802
5	0.1080	[-0.2064, 0.4225]	[-0.3665, 0.5558]	3001|2802

Note: (i) All estimates are computed using a triangular Kernel and local linear regression. (ii) The bandwidth in the first line is the MSE optimal bandwidth.

Table S4. Fuzzy RD estimates at different bandwidths – Life satisfaction – All samples at age 65

Bandwidths	Estimate	95% CI (Conventional)	95% CI (Robust)	Obs L|R
3.8	-0.0567	[-0.3030, 0.1897]	[-0.3504, 0.3503]	2868|2747
1	-0.1129	[-0.8646, 0.6388]	[-1.5143, 1.0699]	2868|2747
1.5	0.0368	[-0.3941, 0.4677]	[-0.7826, 0.5270]	2868|2747
2	-0.0063	[-0.3507, 0.3381]	[-0.4838, 0.5161]	2868|2747
2.5	-0.0227	[-0.3209, 0.2755]	[-0.4026, 0.4511]	2868|2747
3	-0.0422	[-0.3170, 0.2327]	[-0.3788, 0.4020]	2868|2747
3.5	-0.0462	[-0.3002, 0.2079]	[-0.3709, 0.3513]	2868|2747
4	-0.0709	[-0.3119, 0.1701]	[-0.3289, 0.3551]	2868|2747
4.5	-0.1012	[-0.3390, 0.1366]	[-0.3116, 0.3634]	2868|2747
5	-0.1231	[-0.3571, 0.1109]	[-0.3162, 0.3490]	2868|2747

Note: (i) All estimates are computed using a triangular Kernel and local linear regression. (ii) The bandwidth in the first line is the MSE optimal bandwidth.

Table S5. Fuzzy RD estimates at different bandwidths – CESD Scale – All samples at age 65

Bandwidths	Estimate	95% CI (Conventional)	95% CI (Robust)	Obs L|R
3.6	1.4883	[-3.7619, 6.7386]	[-5.3991, 10.2902]	2892|2751
1	7.2653	[-14.5082, 29.0389]	[-26.6100, 50.2505]	2892|2751
1.5	1.7965	[-7.9961, 11.589]	[-7.7480, 23.7890]	2892|2751
2	1.7833	[-5.9205, 9.4871]	[-8.9118, 14.5444]	2892|2751
2.5	1.8201	[-4.6489, 8.2890]	[-7.4046, 12.0228]	2892|2751
3	1.8801	[-3.9705, 7.7307]	[-6.8255, 10.7155]	2892|2751
3.5	1.5895	[-3.7496, 6.9286]	[-5.6404, 10.3139]	2892|2751
4	1.4474	[-3.5288, 6.4235]	[-5.2520, 9.5722]	2892|2751
4.5	1.9047	[-2.9530, 6.7624]	[-5.9824, 8.4645]	2892|2751
5	2.2452	[-2.4975, 6.9879]	[-6.0964, 7.9783]	2892|2751

Note: (i) All estimates are computed using a triangular Kernel and local linear regression. (ii) The bandwidth in the first line is the MSE optimal bandwidth.

Table S6. Fuzzy RD estimates at different bandwidths – MMSE – All samples at age 65

Bandwidths	Estimate	95% CI (Conventional)	95% CI (Robust)	Obs L|R
3.6	-0.4173	[-2.0094, 1.1748]	[-3.9535, 0.6785]	2733|2541
1	-4.1120*	[-10.6881, 2.4640]	[-21.5954, -0.6002]	2733|2541
1.5	-2.0347*	[-5.0309, 0.9615]	[-9.2730, -0.6252]	2733|2541
2	-1.5438	[-3.9104, 0.8229]	[-6.4519, 0.1893]	2733|2541
2.5	-1.0257	[-3.0065, 0.9551]	[-5.3085, 0.2965]	2733|2541
3	-0.6779	[-2.4547, 1.0989]	[-4.6434, 0.4580]	2733|2541
3.5	-0.4491	[-2.0639, 1.1657]	[-4.0503, 0.6380]	2733|2541
4	-0.4660	[-2.0001, 1.0682]	[-3.3839, 1.0889]	2733|2541
4.5	-0.5823	[-2.0929, 0.9283]	[-2.9314, 1.4754]	2733|2541
5	-0.6129	[-2.0813, 0.8556]	[-2.7897, 1.4987]	2733|2541

Note: (i) All estimates are computed using a triangular Kernel and local linear regression. (ii) The bandwidth in the first line is the MSE optimal bandwidth.

(iii) * indicates a significance level at 10%.

Table S7. Fuzzy RD estimates at different bandwidths – ADL – All samples at age 65

Bandwidths	Estimate	95% CI (Conventional)	95% CI (Robust)	Obs L|R
4	0.0394	[-0.3672, 0.4461]	[-0.3964, 0.5935]	3122|2923
1	1.4357*	[-0.7219, 3.5934]	[0.7599, 7.6802]	3122|2923
1.5	0.3946***	[-0.2491, 1.0382]	[0.7361, 2.7326]	3122|2923
2	0.1951*	[-0.3162, 0.7064]	[0.2159, 1.6410]	3122|2923
2.5	0.0667	[-0.3806, 0.5140]	[-0.0357, 1.1917]	3122|2923
3	0.0281	[-0.4067, 0.4629]	[-0.2127, 0.9235]	3122|2923
3.5	0.0231	[-0.3960, 0.4423]	[-0.3191, 0.7270]	3122|2923
4	0.0394	[-0.3672, 0.4461]	[-0.3964, 0.5935]	3122|2923
4.5	0.0628	[-0.3410, 0.4666]	[-0.4476, 0.5340]	3122|2923
5	0.0513	[-0.3494, 0.4520]	[-0.4220, 0.5603]	3122|2923

Note: (i) All estimates are computed using a triangular Kernel and local linear regression. (ii) The bandwidth in the first line is the MSE optimal bandwidth. (iii) *, **, *** indicate significance level at 10%, 5%, 1% respectively.

Table S8. Fuzzy RD estimates at different bandwidths – IADL Scale – All samples at age 65

Bandwidths	Estimate	95% CI (Conventional)	95% CI (Robust)	Obs L|R
2.8	-0.1860	[-0.6735, 0.3015]	[-0.9916, 0.2677]	2720|2872
1	0.0395	[-0.8706, 0.9550]	[-1.4380, 1.7860]	2720|2872
1.5	-0.3436	[-1.0218, 0.3347]	[-0.8885, 1.0386]	2720|2872
2	-0.3054	[-0.9081, 0.2973]	[-1.0164, 0.5247]	2720|2872
2.5	-0.2419	[-0.7621, 0.2782]	[-1.0056, 0.3405]	2720|2872
3	-0.1630	[-0.6356, 0.3096]	[-0.9504, 0.2587]	2720|2872
3.5	-0.1228	[-0.5615, 0.3160]	[-0.8594, 0.2413]	2720|2872
4	-0.0837	[-0.5008, 0.3335]	[-0.8072, 0.2322]	2720|2872
4.5	-0.0416	[-0.4519, 0.3688]	[-0.7873, 0.2398]	2720|2872
5	-0.0413	[-0.4457, 0.3630]	[-0.7034, 0.3215]	2720|2872

Note: (i) All estimates are computed using a triangular Kernel and local linear regression. (ii) The bandwidth in the first line is the MSE optimal bandwidth.

Table S9. Fuzzy RD estimates at different bandwidths – Total Recall Score – All samples at age 65

Bandwidths	Estimate	95% CI (Conventional)	95% CI (Robust)	Obs L|R
3.9	2.3377	[-1.1357, 5.8111]	[-2.0721, 8.1252]	2803|2657
1	-2.705	[-12.9924, 7.5825]	[-31.4250, 2.1186]	2803|2657
1.5	1.2297	[-4.4555, 6.9148]	[-12.9818, 3.8696]	2803|2657
2	3.0086	[-1.9638, 7.9810]	[-8.0789, 6.2567]	2803|2657
2.5	2.7617	[-1.5548, 7.0781]	[-4.4101, 8.0971]	2803|2657
3	2.7142	[-1.2953, 6.7237]	[-3.3379, 8.3971]	2803|2657
3.5	2.5899	[-1.0952, 6.2751]	[-2.6714, 8.1526]	2803|2657
4	2.2463	[-1.1669, 5.6595]	[-1.8819, 8.1363]	2803|2657
4.5	1.9584	[-1.3152, 5.2320]	[-1.6264, 7.9840]	2803|2657
5	1.6654	[-1.4773, 4.8082]	[-1.3710, 7.8473]	2803|2657

Note: (i) All estimates are computed using a triangular Kernel and local linear regression. (ii) The bandwidth in the first line is the MSE optimal bandwidth.

Table S10. Fuzzy RD estimates at different bandwidths – Self-reported health – By gender at age 65

Male					Female				
Bandwidths	Estimate	95% CI (Conventional)	95% CI (Robust)	Obs L|R	Bandwidths	Estimate	95% CI (Conventional)	95% CI (Robust)	Obs L|R
3.2	0.3479	[-0.2031, 0.8988]	[-0.1049, 1.5100]	1709|1610	3.6	-0.3755	[-1.4092, 0.6583]	[-2.5867, 0.4360]	1292|1192
1	0.3966	[-0.4262, 1.2194]	[-0.4153, 2.3239]	1709|1610	1	21.2581	[-660.4485, 702.9648]	[-1381.8309, 881.7232]	1292|1192
1.5	0.4693	[-0.2518, 1.1904]	[-0.7451, 1.4637]	1709|1610	1.5	-2.638	[-14.9302, 9.6541]	[-31.4602, 6.5034]	1292|1192
2	0.5717	[-0.1935, 1.3368]	[-0.7708, 1.4644]	1709|1610	2	-1.0597	[-3.8539, 1.7344]	[-7.8592, 0.4744]	1292|1192
2.5	0.5154	[-0.1675, 1.1983]	[-0.4175, 1.5737]	1709|1610	2.5	-0.6367	[-2.1538, 0.8803]	[-4.1944, 0.3138]	1292|1192
3	0.3923	[-0.2031, 0.9877]	[-0.1733, 1.5712]	1709|1610	3	-0.5361	[-1.7411, 0.6689]	[-3.0544, 0.5056]	1292|1192
3.5	0.3142	[-0.1913, 0.8197]	[-0.0963, 1.3831]	1709|1610	3.5	-0.4060	[-1.4666, 0.6546]	[-2.6450, 0.4595]	1292|1192
4	0.2653	[-0.1874, 0.7180]	[-0.0948, 1.2274]	1709|1610	4	-0.2652	[-1.2000, 0.6696]	[-2.3326, 0.3780]	1292|1192
4.5	0.2527	[-0.1770, 0.6825]	[-0.1563, 1.1003]	1709|1610	4.5	-0.1750	[-1.0703, 0.7203]	[-2.1369, 0.4393]	1292|1192
5	0.2400	[-0.1718, 0.6518]	[-0.1852, 1.0216]	1709|1610	5	-0.1443	[-1.0015, 0.7129]	[-1.8772, 0.5529]	1292|1192

Note: (i) All estimates are computed using a triangular Kernel and local linear regression. (ii) The bandwidth in the first line is the MSE optimal bandwidth.

Table S11. Fuzzy RD estimates at different bandwidths – Life satisfaction – By gender at age 65

Male					Female				
Bandwidths	Estimate	95% CI (Conventional)	95% CI (Robust)	Obs L|R	Bandwidths	Estimate	95% CI (Conventional)	95% CI (Robust)	Obs L|R
2.9	0.2108	[-0.1418, 0.5633]	[-0.2712, 0.6997]	1626|1584	3.8	-0.4077	[-1.2067, 0.3912]	[-1.8136, 0.6824]	1242|1163
1	0.1250	[-0.3358, 0.5857]	[-0.5524, 1.5331]	1626|1584	1	-1.5693	[-11.1657, 8.0271]	[-21.8942, 8.1543]	1242|1163
1.5	0.2158	[-0.1629, 0.5945]	[-0.6304, 0.7355]	1626|1584	1.5	-0.5417	[-2.6100, 1.5266]	[-4.6881, 1.2601]	1242|1163
2	0.2168	[-0.1505, 0.5842]	[-0.4618, 0.7075]	1626|1584	2	-0.5129	[-1.8940, 0.8683]	[-2.8103, 1.2062]	1242|1163
2.5	0.2333	[-0.1299, 0.5965]	[-0.3596, 0.7018]	1626|1584	2.5	-0.4942	[-1.5696, 0.5812]	[-2.1741, 1.0101]	1242|1163
3	0.2034	[-0.1458, 0.5526]	[-0.2516, 0.6984]	1626|1584	3	-0.4621	[-1.3732, 0.4491]	[-1.9497, 0.8189]	1242|1163
3.5	0.1569	[-0.1541, 0.4679]	[-0.1262, 0.6729]	1626|1584	3.5	-0.4180	[-1.2508, 0.4148]	[-1.8852, 0.7010]	1242|1163
4	0.1032	[-0.1835, 0.3900]	[-0.0518, 0.6611]	1626|1584	4	-0.4111	[-1.1887, 0.3666]	[-1.7398, 0.6862]	1242|1163
4.5	0.2527	[-0.1770, 0.6825]	[-0.1563, 1.1003]	1709|1610	4.5	-0.4609	[-1.2620, 0.3401]	[-1.6387, 0.8433]	1242|1163
5	0.2400	[-0.1718, 0.6518]	[-0.1852, 1.0216]	1709|1610	5	-0.5012	[-1.3024, 0.3001]	[-1.5824, 0.8665]	1242|1163

Note: (i) All estimates are computed using a triangular Kernel and local linear regression. (ii) The bandwidth in the first line is the MSE optimal bandwidth.

Table S12. Fuzzy RD estimates at different bandwidths – CESD Scale – By gender at age 65

Male					Female				
Bandwidths	Estimate	95% CI (Conventional)	95% CI (Robust)	Obs L|R	Bandwidths	Estimate	95% CI (Conventional)	95% CI (Robust)	Obs L|R
2.6	-0.0820	[-7.3509, 7.187]	[-12.7522, 9.3024]	1636|1583	3.9	0.5681	[-13.562, 14.6982]	[-13.3049, 28.2534]	1256|1168
1	-0.0579	[-10.8867, 10.7708]	[-20.6684, 15.7711]	1636|1583	1	1989.868	[-535144.3067, 539124.0423]	[-679437.188, 1089168.5474]	1256|1168
1.5	-1.4327	[-9.942, 7.0766]	[-12.8373, 14.2046]	1636|1583	1.5	19.4501	[-74.1878, 113.0881]	[-53.4963, 242.2248]	1256|1168
2	-0.8207	[-8.9096, 7.2683]	[-14.3986, 10.2217]	1636|1583	2	8.1348	[-23.8998, 40.1694]	[-18.9281, 78.0463]	1256|1168
2.5	-0.1113	[-7.5306, 7.3081]	[-13.1124, 9.3676]	1636|1583	2.5	4.5466	[-15.7969, 24.89]	[-14.7806, 45.7037]	1256|1168
3	0.3831	[-6.6167, 7.3829]	[-12.3606, 8.8622]	1636|1583	3	3.0557	[-13.4324, 19.5437]	[-13.9327, 34.9419]	1256|1168
3.5	0.6762	[-5.6274, 6.9798]	[-10.6276, 8.4702]	1636|1583	3.5	1.3013	[-13.7275, 16.3302]	[-12.8618, 31.4481]	1256|1168
4	0.7978	[-5.0538, 6.6493]	[-9.4462, 8.2264]	1636|1583	4	0.5048	[-13.3833, 14.3929]	[-13.5265, 27.2934]	1256|1168
4.5	1.4381	[-4.2027, 7.0788]	[-9.4697, 7.4854]	1636|1583	4.5	0.8086	[-12.9747, 14.5919]	[-16.163, 24.3291]	1256|1168
5	2.0571	[-3.4293, 7.5434]	[-9.3071, 7.099]	1636|1583	5	0.8445	[-12.4187, 14.1077]	[-16.5065, 22.3724]	1256|1168

Note: (i) All estimates are computed using a triangular Kernel and local linear regression. (ii) The bandwidth in the first line is the MSE optimal bandwidth.

Table S13. Fuzzy RD estimates at different bandwidths – MMSE – By gender at age 65

Male					Female				
Bandwidths	Estimate	95% CI (Conventional)	95% CI (Robust)	Obs L|R	Bandwidths	Estimate	95% CI (Conventional)	95% CI (Robust)	Obs L|R
3.1	-1.9217	[-4.4678, 0.6244]	[-6.4228, 0.6868]	1557|1484	3.7	2.3324	[-2.8797, 7.5444]	[-7.7233, 7.7472]	1176|1057
1	-2.5408	[-6.4288, 1.3471]	[-10.8689, 1.6421]	1557|1484	1	-6.3453	[-28.7836, 16.093]	[-54.5053, 18.1423]	1176|1057
1.5	-2.5718	[-5.7876, 0.6439]	[-7.2529, 1.8389]	1557|1484	1.5	-0.7421	[-7.8411, 6.3568]	[-19.338, 2.0446]	1176|1057
2	-2.5651	[-5.711, 0.5808]	[-6.883, 1.6835]	1557|1484	2	0.2890	[-5.2399, 5.8179]	[-11.1598, 5.0983]	1176|1057
2.5	-2.2481	[-5.1015, 0.6053]	[-6.7903, 1.0153]	1557|1484	2.5	1.1956	[-3.9189, 6.3102]	[-9.2713, 5.7418]	1176|1057
3	-1.9824	[-4.6022, 0.6373]	[-6.5076, 0.7776]	1557|1484	3	1.8348	[-3.1756, 6.8452]	[-8.307, 6.5137]	1176|1057
3.5	-1.6606	[-3.9349, 0.6136]	[-6.0573, 0.3729]	1557|1484	3.5	2.2578	[-2.9258, 7.4413]	[-7.9753, 7.4068]	1176|1057
4	-1.6141	[-3.7403, 0.5121]	[-5.4225, 0.6445]	1557|1484	4	2.2048	[-2.8303, 7.2398]	[-6.6006, 8.2788]	1176|1057
4.5	-1.6708	[-3.7322, 0.3907]	[-4.9893, 0.9382]	1557|1484	4.5	1.9955	[-2.9831, 6.9741]	[-5.6989, 8.9342]	1176|1057
5	-1.6813	[-3.6595, 0.2968]	[-4.7754, 0.9631]	1557|1484	5	1.9403	[-2.8668, 6.7475]	[-5.2055, 8.8495]	1176|1057

Note: (i) All estimates are computed using a triangular Kernel and local linear regression. (ii) The bandwidth in the first line is the MSE optimal bandwidth.

Table S14. Fuzzy RD estimates at different bandwidths – ADL – By gender at age 65

Male					Female				
Bandwidths	Estimate	95% CI (Conventional)	95% CI (Robust)	Obs L|R	Bandwidths	Estimate	95% CI (Conventional)	95% CI (Robust)	Obs L|R
2.9	-0.0172	[-0.6949, 0.6605]	[-0.6117, 1.0061]	1820|1672	3.1	0.2526	[-0.5537, 1.0589]	[-0.3121, 2.4776]	1302|1251
1	0.8498	[8e-04, 1.6988]	[-0.1631, 2.4864]	1820|1672	1	6.8682	[-91.4755, 105.2118]	[-98.6259, 226.6819]	1302|1251
1.5	0.2184**	[-0.3892, 0.8261]	[0.3275, 2.3098]	1820|1672	1.5	1.3199	[-3.2060, 5.8458]	[-2.5456, 11.7096]	1302|1251
2	0.0233	[-0.6377, 0.6843]	[-0.0828, 1.7340]	1820|1672	2	0.9155	[-1.2241, 3.0550]	[-1.0583, 5.5888]	1302|1251
2.5	-0.0312	[-0.7048, 0.6425]	[-0.4369, 1.2612]	1820|1672	2.5	0.4704	[-0.6153, 1.5561]	[-0.2998, 3.3916]	1302|1251
3	-0.0146	[-0.6913, 0.6621]	[-0.6326, 0.9668]	1820|1672	3	0.2702	[-0.5457, 1.0861]	[-0.2786, 2.5797]	1302|1251
3.5	0.0044	[-0.6244, 0.6333]	[-0.6755, 0.7793]	1820|1672	3.5	0.208	[-0.5917, 1.0078]	[-0.4400, 2.1104]	1302|1251
4	0.0584	[-0.5351, 0.6518]	[-0.7399, 0.6431]	1820|1672	4	0.1331	[-0.6438, 0.9100]	[-0.4316, 1.7563]	1302|1251
4.5	0.092	[-0.4859, 0.6699]	[-0.7421, 0.6405]	1820|1672	4.5	0.1339	[-0.6551, 0.9228]	[-0.5793, 1.4579]	1302|1251
5	0.0831	[-0.4851, 0.6513]	[-0.6824, 0.7107]	1820|1672	5	0.1016	[-0.6642, 0.8674]	[-0.5672, 1.3152]	1302|1251

Note: (i) All estimates are computed using a triangular Kernel and local linear regression. (ii) The bandwidth in the first line is the MSE optimal bandwidth.
(iii) ** indicate significance level at 5%..

Table S15. Fuzzy RD estimates at different bandwidths – IADL Scale – By gender at age 65

Male					Female				
Bandwidths	Estimate	95% CI (Conventional)	95% CI (Robust)	Obs L|R	Bandwidths	Estimate	95% CI (Conventional)	95% CI (Robust)	Obs L|R
2.2	-0.7394	[-1.8003, 0.3215]	[-1.8990, 0.9691]	1554|1651	3.1	0.3889	[-0.5219, 1.2998]	[-0.6503, 2.0774]	1166|1221
1	-0.3256	[-1.1992, 0.5481]	[-2.3972, 1.0077]	1554|1651	1	1.2933	[-4.0734, 6.6600]	[-4.6470, 13.6277]	1166|1221
1.5	-0.6629	[-1.5841, 0.2584]	[-1.5594, 1.3068]	1554|1651	1.5	0.5481	[-1.1858, 2.2820]	[-0.9827, 4.1595]	1166|1221
2	-0.7432	[-1.7706, 0.2842]	[-1.7746, 1.0596]	1554|1651	2	0.5665	[-0.8342, 1.9673]	[-1.0734, 2.8577]	1166|1221
2.5	-0.6683	[-1.6330, 0.2965]	[-1.9716, 0.5914]	1554|1651	2.5	0.4947	[-0.5896, 1.5790]	[-0.9009, 2.3279]	1166|1221
3	-0.5053	[-1.3506, 0.3401]	[-1.9412, 0.2194]	1554|1651	3	0.4137	[-0.5137, 1.3410]	[-0.6954, 2.1028]	1166|1221
3.5	-0.3301*	[-1.0309, 0.3706]	[-1.7619, -0.0159]	1554|1651	3.5	0.2610	[-0.5788, 1.1008]	[-0.4297, 1.9650]	1166|1221
4	-0.1791*	[-0.8021, 0.4439]	[-1.6096, -0.0626]	1554|1651	4	0.1011	[-0.6704, 0.8725]	[-0.2503, 1.8032]	1166|1221
4.5	-0.0825	[-0.6683, 0.5033]	[-1.4444, 0.0337]	1554|1651	4.5	0.0567	[-0.7244, 0.8377]	[-0.4086, 1.5801]	1166|1221
5	-0.059	[-0.6234, 0.5054]	[-1.2519, 0.2028]	1554|1651	5	0.0103	[-0.7524, 0.7730]	[-0.4789, 1.4165]	1166|1221

Note: (i) All estimates are computed using a triangular Kernel and local linear regression. (ii) The bandwidth in the first line is the MSE optimal bandwidth.
(iii) * indicate significance level at 10%.

Table S16. Fuzzy RD estimates at different bandwidths – Total recall score – By gender at age 65

Male					Female				
Bandwidths	Estimate	95% CI (Conventional)	95% CI (Robust)	Obs L|R	Bandwidths	Estimate	95% CI (Conventional)	95% CI (Robust)	Obs L|R
3	-2.0079	[-7.3580, 3.3422]	[-7.2852, 7.9391]	1587|1533	3	8.8388	[-5.0985, 22.7760]	[-12.6402, 29.1719]	1216|1124
1	-0.8132	[-7.2062, 5.5798]	[-14.4197, 6.7452]	1587|1533	1	-1.0791	[-23.2813, 21.1231]	[-50.8273, 22.1633]	1216|1124
1.5	-0.9201	[-6.3528, 4.5126]	[-9.2897, 6.3735]	1587|1533	1.5	6.6848	[-13.1517, 26.5213]	[-33.8746, 26.9177]	1216|1124
2	-0.6231	[-6.0736, 4.8275]	[-8.3733, 6.6697]	1587|1533	2	9.0889	[-8.6784, 26.8562]	[-22.6625, 30.6305]	1216|1124
2.5	-1.6549	[-7.0621, 3.7523]	[-6.8965, 8.2822]	1587|1533	2.5	8.9127	[-6.1361, 23.9614]	[-15.5443, 29.3264]	1216|1124
3	-2.0079	[-7.3580, 3.3422]	[-7.2852, 7.9391]	1587|1533	3	8.8388	[-5.0985, 22.7760]	[-12.6402, 29.1719]	1216|1124
3.5	-2.0603	[-6.9143, 2.7936]	[-7.5372, 6.3253]	1587|1533	3.5	9.1657	[-4.8879, 23.2193]	[-13.0641, 29.0291]	1216|1124
4	-2.2052	[-6.7541, 2.3437]	[-7.5747, 5.4305]	1587|1533	4	8.4478	[-4.1705, 21.0661]	[-9.1042, 28.4361]	1216|1124
4.5	-2.2378	[-6.6006, 2.1250]	[-7.7773, 4.7588]	1587|1533	4.5	7.6278	[-3.9689, 19.2245]	[-6.6528, 27.5932]	1216|1124
5	-2.2999	[-6.5045, 1.9048]	[-7.7460, 4.3904]	1587|1533	5	6.8328	[-3.5750, 17.2405]	[-4.4282, 26.0819]	1216|1124

Note: (i) All estimates are computed using a triangular Kernel and local linear regression. (ii) The bandwidth in the first line is the MSE optimal bandwidth.
.

Table S17. Fuzzy RD estimates at different bandwidths – Self-reported health – By education at age 65

Low Educated					High Educated				
Bandwidths	Estimate	95% CI (Conventional)	95% CI (Robust)	Obs L|R	Bandwidths	Estimate	95% CI (Conventional)	95% CI (Robust)	Obs L|R
3.1	0.1037	[-0.4305, 0.6379]	[-0.5362, 0.9856]	1075|2270	4.2	0.2610	[-0.4065, 0.9286]	[-0.4855, 1.4509]	1274|1184
1	0.2323	[-0.3605, 0.8251]	[-0.2917, 1.5335]	1075|2270	1	0.2480	[-0.4073, 0.9033]	[-0.6388, 1.0363]	1274|1184
1.5	0.1501	[-0.3558, 0.6559]	[-0.4378, 0.9937]	1075|2270	1.5	0.4703	[-0.3518, 1.2923]	[-1.1477, 1.1277]	1274|1184
2	0.1540	[-0.3718, 0.6799]	[-0.5391, 0.9351]	1075|2270	2	0.4798	[-0.2974, 1.2571]	[-0.6928, 1.4444]	1274|1184
2.5	0.1467	[-0.3841, 0.6776]	[-0.5451, 0.9530]	1075|2270	2.5	0.3916	[-0.3080, 1.0913]	[-0.4478, 1.5134]	1274|1184
3	0.1083	[-0.4265, 0.6432]	[-0.5313, 0.9899]	1075|2270	3	0.3398	[-0.3586, 1.0381]	[-0.4353, 1.5570]	1274|1184
3.5	0.1111	[-0.4034, 0.6257]	[-0.5685, 0.9045]	1075|2270	3.5	0.2961	[-0.3723, 0.9645]	[-0.4221, 1.4986]	1274|1184
4	0.1072	[-0.3775, 0.5919]	[-0.5455, 0.8506]	1075|2270	4	0.2694	[-0.3976, 0.9364]	[-0.4680, 1.4607]	1274|1184
4.5	0.1256	[-0.3394, 0.5906]	[-0.5617, 0.7840]	1075|2270	4.5	0.2455	[-0.4083, 0.8992]	[-0.4838, 1.4162]	1274|1184
5	0.1153	[-0.3275, 0.5580]	[-0.5044, 0.7823]	1075|2270	5	0.2248	[-0.4142, 0.8637]	[-0.4988, 1.3608]	1274|1184

Note: (i) All estimates are computed using a triangular Kernel and local linear regression. (ii) The bandwidth in the first line is the MSE optimal bandwidth.

Table S18. Fuzzy RD estimates at different bandwidths – Life satisfaction – By education at age 65

Low Educated					High Educated				
Bandwidths	Estimate	95% CI (Conventional)	95% CI (Robust)	Obs L|R	Bandwidths	Estimate	95% CI (Conventional)	95% CI (Robust)	Obs L|R
3	0.0183	[-0.3591, 0.3956]	[-0.1869, 0.7948]	1339|1905	2.7	-0.1490	[-0.5675, 0.2694]	[-0.9286, 0.3162]	1529|842
1	0.3056	[-0.7440, 1.3553]	[-1.2471, 2.5727]	1339|1905	1	-0.6085	[-2.5244, 1.3073]	[-3.3045, 2.7108]	1529|842
1.5	0.3073	[-0.3342, 0.9488]	[-0.6475, 1.3159]	1339|1905	1.5	-0.2845	[-1.0476, 0.4786]	[-1.6916, 0.5533]	1529|842
2	0.2137	[-0.3150, 0.7424]	[-0.3664, 1.1281]	1339|1905	2	-0.2233	[-0.7472, 0.3006]	[-1.1220, 0.4049]	1529|842
2.5	0.1105	[-0.3112, 0.5322]	[-0.1944, 0.9418]	1339|1905	2.5	-0.1643	[-0.6009, 0.2723]	[-0.9673, 0.3224]	1529|842
3	0.0183	[-0.3591, 0.3956]	[-0.1869, 0.7948]	1339|1905	3	-0.1194	[-0.5183, 0.2795]	[-0.8935, 0.3026]	1529|842
3.5	-0.0263	[-0.3746, 0.3220]	[-0.2397, 0.6604]	1339|1905	3.5	-0.0838	[-0.4553, 0.2877]	[-0.8269, 0.3009]	1529|842
4	-0.0660	[-0.3963, 0.2643]	[-0.2670, 0.5971]	1339|1905	4	-0.0885	[-0.4466, 0.2696]	[-0.7254, 0.3564]	1529|842
4.5	-0.1045	[-0.4309, 0.2220]	[-0.3011, 0.5706]	1339|1905	4.5	-0.1064	[-0.4646, 0.2519]	[-0.6622, 0.4100]	1529|842
5	-0.1304	[-0.4526, 0.1918]	[-0.3435, 0.5286]	1339|1905	5	-0.1246	[-0.4803, 0.2311]	[-0.6222, 0.4386]	1529|842

Note: (i) All estimates are computed using a triangular Kernel and local linear regression. (ii) The bandwidth in the first line is the MSE optimal bandwidth.

Table S19. Fuzzy RD estimates at different bandwidths – CESD Scale – By education at age 65

Low Educated					High Educated				
Bandwidths	Estimate	95% CI (Conventional)	95% CI (Robust)	Obs L|R	Bandwidths	Estimate	95% CI (Conventional)	95% CI (Robust)	Obs L|R
3.1	4.5933	[-4.5766, 13.7633]	[-7.2359, 21.1120]	1355|1909	3.3	-0.7378	[-7.7782, 6.3026]	[-11.9882, 8.1417]	1537|842
1	12.8922	[-25.8925, 51.6769]	[-34.3597, 104.1168]	1355|1909	1	1.0046	[-23.2886, 25.2979]	[-37.1072, 35.1851]	1537|842
1.5	6.3741	[-10.2409, 22.9890]	[-11.1849, 43.3931]	1355|1909	1.5	-2.9237	[-16.0004, 10.1531]	[-19.9791, 19.9183]	1537|842
2	6.5653	[-7.8863, 21.0169]	[-13.9418, 30.8501]	1355|1909	2	-2.0343	[-11.1765, 7.1079]	[-16.2145, 10.2977]	1537|842
2.5	5.4788	[-5.6337, 16.5913]	[-8.9005, 25.4034]	1355|1909	2.5	-1.3118	[-9.0919, 6.4682]	[-13.8347, 8.3703]	1537|842
3	4.6340	[-4.7347, 14.0026]	[-7.2364, 21.7181]	1355|1909	3	-0.7203	[-7.9968, 6.5561]	[-12.8717, 7.8984]	1537|842
3.5	3.7120	[-4.4049, 11.8289]	[-5.2911, 19.6121]	1355|1909	3.5	-0.6621	[-7.6097, 6.2856]	[-11.7662, 8.1211]	1537|842
4	2.6811	[-4.5606, 9.9229]	[-4.129, 17.9318]	1355|1909	4	-0.0847	[-6.8103, 6.6410]	[-11.9001, 7.3165]	1537|842
4.5	2.6002	[-4.3178, 9.5182]	[-5.1024, 15.8800]	1355|1909	4.5	0.8457	[-5.9077, 7.5991]	[-12.6144, 6.7079]	1537|842
5	2.6665	[-4.0105, 9.3435]	[-5.7707, 14.3222]	1355|1909	5	1.4520	[-5.2827, 8.1866]	[-12.4272, 6.9578]	1537|842

Note: (i) All estimates are computed using a triangular Kernel and local linear regression. (ii) The bandwidth in the first line is the MSE optimal bandwidth.

Table S20. Fuzzy RD estimates at different bandwidths – MMSE – By education at age 65

Low Educated					High Educated				
Bandwidths	Estimate	95% CI (Conventional)	95% CI (Robust)	Obs L|R	Bandwidths	Estimate	95% CI (Conventional)	95% CI (Robust)	Obs L|R
2.9	-3.2411*	[-6.9105, 0.4283]	[-10.835, -0.0711]	1241|1720	2.6	1.3649	[-0.9414, 3.6711]	[-3.4222, 3.2389]	1492|821
1	-5.7041	[-18.0135, 6.6054]	[-35.2219, 3.9051]	1241|1720	1	-2.9704	[-9.9197, 3.9789]	[-21.8843, 0.5281]	1492|821
1.5	-4.6958	[-11.5427, 2.1512]	[-17.0152, 2.9751]	1241|1720	1.5	0.2724	[-2.6939, 3.2387]	[-7.8675, 0.6076]	1492|821
2	-4.7982	[-11.0826, 1.4862]	[-14.6964, 3.3361]	1241|1720	2	0.8730	[-1.5763, 3.3223]	[-4.2167, 2.5491]	1492|821
2.5	-3.8137	[-8.2871, 0.6598]	[-12.3314, 0.7000]	1241|1720	2.5	1.2712	[-1.0320, 3.5743]	[-3.4368, 3.1586]	1492|821
3	-3.1255*	[-6.6517, 0.4007]	[-10.5419, -0.1733]	1241|1720	3	1.6374	[-0.6708, 3.9456]	[-3.1943, 3.6122]	1492|821
3.5	-2.5106*	[-5.3989, 0.3778]	[-9.1558, -0.6713]	1241|1720	3.5	1.707	[-0.4992, 3.9132]	[-2.5599, 4.0355]	1492|821
4	-2.3161*	[-4.9664, 0.3342]	[-7.9861, -0.2415]	1241|1720	4	1.6204	[-0.4793, 3.7200]	[-1.8970, 4.3963]	1492|821
4.5	-2.2872	[-4.8512, 0.2768]	[-7.2322, 0.2221]	1241|1720	4.5	1.4863	[-0.5643, 3.5368]	[-1.4694, 4.6766]	1492|821
5	-2.0955	[-4.5032, 0.3121]	[-6.8579, 0.1068]	1241|1720	5	1.3075	[-0.6699, 3.2849]	[-1.1008, 4.8371]	1492|821

Note: (i) All estimates are computed using a triangular Kernel and local linear regression. (ii) The bandwidth in the first line is the MSE optimal bandwidth.
(iii) * indicate significance level at 10%.

Table S21. Fuzzy RD estimates at different bandwidths – ADL – By education at age 65

Low Educated					High Educated				
Bandwidths	Estimate	95% CI (Conventional)	95% CI (Robust)	Obs L|R	Bandwidths	Estimate	95% CI (Conventional)	95% CI (Robust)	Obs L|R
3.7	0.0854	[-0.3663, 0.5371]	[-0.2039, 0.9728]	1454|2012	4.1	0.0179	[-0.6804, 0.7161]	[-0.9862, 0.7475]	1668|911
1	1.6791*	[-1.8872, 5.2454]	[0.5349, 12.1784]	1454|2012	1	1.3315	[-1.6804, 4.3433]	[-1.8291, 7.7936]	1668|911
1.5	0.6873*	[-0.4134, 1.7880]	[0.4428, 3.8974]	1454|2012	1.5	0.1013*	[-0.7889, 0.9916]	[0.0199, 2.9597]	1668|911
2	0.5218*	[-0.3230, 1.3666]	[0.0825, 2.6875]	1454|2012	2	-0.0647	[-0.8285, 0.6991]	[-0.4563, 1.4944]	1668|911
2.5	0.2221*	[-0.3131, 0.7574]	[0.1621, 1.9872]	1454|2012	2.5	-0.0600	[-0.7875, 0.6674]	[-0.7269, 0.9763]	1668|911
3	0.1170	[-0.3558, 0.5899]	[-0.0411, 1.4454]	1454|2012	3	-0.0490	[-0.7869, 0.6889]	[-0.8529, 0.8797]	1668|911
3.5	0.0990	[-0.3537, 0.5516]	[-0.1843, 1.0557]	1454|2012	3.5	-0.0400	[-0.7668, 0.6867]	[-0.9031, 0.8376]	1668|911
4	0.0909	[-0.3630, 0.5448]	[-0.2640, 0.8320]	1454|2012	4	0.0090	[-0.6921, 0.7102]	[-0.9762, 0.7520]	1668|911
4.5	0.1088	[-0.3588, 0.5764]	[-0.3387, 0.7137]	1454|2012	4.5	0.0500	[-0.6371, 0.7371]	[-1.0103, 0.7512]	1668|911
5	0.1029	[-0.3726, 0.5784]	[-0.3473, 0.6905]	1454|2012	5	0.0405	[-0.6309, 0.7120]	[-0.9418, 0.8432]	1668|911

Note: (i) All estimates are computed using a triangular Kernel and local linear regression. (ii) The bandwidth in the first line is the MSE optimal bandwidth.
(iii) * indicate significance level at 10%.

Table S22. Fuzzy RD estimates at different bandwidths – IADL Scale – By education at age 65

Low Educated					High Educated				
Bandwidths	Estimate	95% CI (Conventional)	95% CI (Robust)	Obs L|R	Bandwidths	Estimate	95% CI (Conventional)	95% CI (Robust)	Obs L|R
2.6	-0.0380	[-0.7084, 0.6323]	[-1.1225, 0.7310]	1251|1968	3.7	-0.2889	[-0.9966, 0.4189]	[-1.3405, 0.4968]	1469|904
1	0.0685	[-1.5592, 1.6961]	[-2.3346, 3.6564]	1251|1968	1	0.0453	[-1.0647, 1.1553]	[-1.9032, 1.9639]	1469|904
1.5	-0.2811	[-1.2231, 0.6609]	[-1.4205, 1.600]	1251|1968	1.5	-0.3975	[-1.3981, 0.6032]	[-1.2051, 1.4696]	1469|904
2	-0.1227	[-0.9755, 0.7300]	[-1.452, 0.9249]	1251|1968	2	-0.4279	[-1.3060, 0.4503]	[-1.3003, 0.8383]	1469|904
2.5	-0.0584	[-0.7555, 0.6387]	[-1.1584, 0.7759]	1251|1968	2.5	-0.3697	[-1.1469, 0.4076]	[-1.3858, 0.5412]	1469|904
3	0.0207	[-0.5876, 0.6291]	[-0.9583, 0.6423]	1251|1968	3	-0.3143	[-1.0568, 0.4282]	[-1.3948, 0.4957]	1469|904
3.5	0.0595	[-0.4928, 0.6117]	[-0.7877, 0.5906]	1251|1968	3.5	-0.2992	[-1.0156, 0.4173]	[-1.3523, 0.5027]	1469|904
4	0.0611	[-0.4673, 0.5896]	[-0.6866, 0.6065]	1251|1968	4	-0.2363	[-0.9139, 0.4413]	[-1.3551, 0.4039]	1469|904
4.5	0.0730	[-0.4581, 0.6041]	[-0.6711, 0.6257]	1251|1968	4.5	-0.1658	[-0.8184, 0.4868]	[-1.3517, 0.3587]	1469|904
5	0.0620	[-0.4659, 0.5899]	[-0.6128, 0.6763]	1251|1968	5	-0.1538	[-0.7917, 0.4840]	[-1.2552, 0.4553]	1469|904

Note: (i) All estimates are computed using a triangular Kernel and local linear regression. (ii) The bandwidth in the first line is the MSE optimal bandwidth.

Table S23. Fuzzy RD estimates at different bandwidths – Total recall score – By education at age 65

Low Educated					High Educated				
Bandwidths	Estimate	95% CI (Conventional)	95% CI (Robust)	Obs L|R	Bandwidths	Estimate	95% CI (Conventional)	95% CI (Robust)	Obs L|R
2.7	2.1123	[-4.0938, 8.3185]	[-6.7440, 10.7301]	1294|1823	2.6	2.5517	[-2.0053, 7.1088]	[-5.3186, 7.6225]	1509|834
1	0.9066	[-10.1851, 11.9984]	[-13.3454, 19.8756]	1294|1823	1	-8.1809*	[-29.0404, 12.6787]	[-73.1642, -5.0546]	1509|834
1.5	0.9129	[-6.9476, 8.7733]	[-9.5736, 12.2866]	1294|1823	1.5	0.8105*	[-5.7406, 7.3615]	[-20.8791, -2.1004]	1509|834
2	2.3885	[-5.3057, 10.0826]	[-10.4985, 10.5675]	1294|1823	2	2.6453	[-2.6004, 7.8910]	[-9.7701, 5.0061]	1509|834
2.5	2.1451	[-4.4684, 8.7587]	[-7.4148, 11.0517]	1294|1823	2.5	2.4855	[-2.0986, 7.0696]	[-5.4566, 7.5087]	1509|834
3	2.1496	[-3.7939, 8.0931]	[-6.2970, 10.5918]	1294|1823	3	2.6682	[-1.8045, 7.1410]	[-4.6663, 8.0908]	1509|834
3.5	1.9742	[-3.3313, 7.2796]	[-5.2143, 9.9944]	1294|1823	3.5	2.8003	[-1.5400, 7.1406]	[-4.3576, 8.0955]	1509|834
4	1.8216	[-3.0169, 6.6601]	[-4.6029, 9.2828]	1294|1823	4	2.5137	[-1.6700, 6.6975]	[-3.3203, 8.6774]	1509|834
4.5	1.7611	[-2.8101, 6.3322]	[-4.4059, 8.7394]	1294|1823	4.5	2.1898	[-1.9680, 6.3475]	[-2.8023, 9.1130]	1509|834
5	1.5275	[-2.8065, 5.8616]	[-3.8052, 8.6422]	1294|1823	5	2.0076	[-2.1176, 6.1328]	[-2.7400, 9.1110]	1509|834

Note: (i) All estimates are computed using a triangular Kernel and local linear regression. (ii) The bandwidth in the first line is the MSE optimal bandwidth.

Table S24. Nonparametric fuzzy regression discontinuity (RD) estimates—Gender heterogeneity at age 65

	Smoking		Drinking		Physical Activity	
	Male	Female	Male	Female	Male	Female
MSE Optimal						
Bandwidth	2.7	3.3	2.6	3.4	3.1	3.4
Point Estimate	0.3905	0.0198	0.3367	-0.4098	-0.1775	0.0838
Conventional 95% CI	[-0.3478, 1.1289]	[-0.4446, 0.4841]	[-0.3981, 1.0714]	[-1.6618, 0.8423]	[-0.4870, 0.1321]	[-0.2604, 0.4281]
Robust 95% CI	[-0.4624, 1.2591]	[-0.5419, 0.5875]	[-0.5524, 1.1842]	[-1.8583, 1.1398]	[-0.5349, 0.1782]	[-0.3378, 0.4700]
Effective obs L|R	455|401	453|439	495|454	452|441	311|341	315|293
CER Optimal						
Bandwidth	1.8	2.2	1.8	2.3	2.1	2.4
Point Estimate	0.0633	0.0205	0.0207	-0.2929	-0.0846	-0.0186
Conventional 95% CI	[-0.7083, 0.8349]	[-0.5580, 0.5990]	[-0.7799, 0.8213]	[-1.7301, 1.1444]	[-0.4170, 0.2479]	[-0.3319, 0.2946]
Robust 95% CI	[-0.7487, 0.8915]	[-0.6005, 0.6441]	[-0.8456, 0.867]	[-1.8202, 1.2819]	[-0.4400, 0.2687]	[-0.3608, 0.3165]
Effective obs L|R	322|276	299|300	333|287	298|300	232|219	217|204
Obs L|R	1628|1452	1370|1248	1821|1673	1370|1251	1020|1061	786|805

Note: (i) All estimates are computed using a triangular Kernel and nearest neighbor heteroskedasticity-robust variance estimator as suggested by CCT framework. (ii) Effective number of observations depend on the size of the data-driven selected bandwidth.
All of the p-values > 0.05.

Table S25. Nonparametric fuzzy regression discontinuity (RD) estimates—Education heterogeneity at age 65

	Smoking		Drinking		Physical Activity	
	Low educated	High educated	Low educated	High educated	Low educated	High educated
MSE Optimal						
Bandwidth	3.3	3.8	2.6	3.9	2.9	3.6
Point Estimate	0.2023	-0.2615	-0.0300	-0.4835	-0.0367	-0.0543
Conventional 95% CI	[-0.4505, 0.8550]	[-0.8219, 0.2989]	[-0.9143, 0.8544]	[-1.1796, 0.2126]	[-0.328, 0.2546]	[-0.2399, 0.1313]
Robust 95% CI	[-0.5527, 1.0100]	[-0.9539, 0.3703]	[-1.2407, 0.8613]	[-1.2607, 0.4070]	[-0.3515, 0.3231]	[-0.3154, 0.1283]
Effective obs L|R	478|572	559|407	429|474	625|453	258|317	373|284
CER Optimal						
Bandwidth	2.2	2.6	1.7	2.6	2	2.5
Point Estimate	0.0043	-0.2025	-0.3487	-0.2791	-0.0764	-0.0212
Conventional 95% CI	[-0.9160, 0.9246]	[-0.8393, 0.4343]	[-1.6528, 0.9555]	[-1.0221, 0.4640]	[-0.4574, 0.3047]	[-0.1939, 0.1515]
Robust 95% CI	[-0.9651, 1.0175]	[-0.9039, 0.4692]	[-1.8367, 0.9567]	[-1.0603, 0.5469]	[-0.4725, 0.3449]	[-0.2308, 0.1512]
Effective obs L|R	345|363	382|297	292|318	416|324	181|227	269|205
Obs L|R	1387|1844	1611|856	1489|2013	1702|911	843|1258	963|608

Note: (i) All estimates are computed using a triangular Kernel and nearest neighbor heteroskedasticity-robust variance estimator as suggested by CCT framework.
(ii) Effective number of observations depend on the size of the data-driven selected bandwidth.
All of the p-values > 0.05.

Table S26. Formal falsification test for the validity of the Continuity-Based Analysis for predetermined Covariates

variables	MSE Optimal Bandwidth	RD Estimator	Robust Inference P-value	Robust Inference [Confidence Interval]	Effective L|R	L|R	Observations
Males	1.843	-0.07305	0.2808	[-0.20642, 0.05989]	606|590	3196|2926	6122
Rural Hukou	1.447	0.01287	0.8694	[-0.12558, 0.14858]	481|471	3196|2926	6122
Education	1.642	0.05858	0.3472	[-0.07202, 0.20478]	536|517	3196|2926	6122
Divorced	2.022	-0.00073	0.9628	[-0.03624, 0.03456]	666|625	3196|2926	6122
Married	2.543	0.00209	0.9425	[-0.07451, 0.08021]	819|766	3196|2926	6122
Never Married	1.904	-0.01302	0.1677	[-0.03351, 0.00582]	628|601	3196|2926	6122
Partnered	2.576	-0.01338	0.6344	[-0.07024, 0.04281]	819|766	3196|2926	6122
Separated	1.797	0.00701	0.3065	[-0.00701, 0.02231]	558|539	3196|2926	6122
Widowed	2.535	0.01591	0.4839	[-0.02894, 0.0611]	819|766	3196|2926	6122

Notes: RD estimated using local polynomial estimator with triangular kernel weights and the MSE-optimal bandwidth selector. Since each covariate may have a different conditional mean function and different curvature, the optimal bandwidth for local polynomial estimation and inference will also be different for every variable; i.e., the statistical analysis is conducted separately for each covariate using the local polynomial estimators. (ii) the number of effective observations used in the analysis varies for each covariate, which occurs because the MSE-optimal bandwidth is different for every covariate tested for continuity.

Table S27. Manipulation test of running variable

Method	Test statistic	P-value
Unrestricted		
Conventional	-0.5373	0.5911
Robust	-0.6405	0.5219
Restricted		
Conventional	-0.2731	0.7848
Robust	-0.2549	0.7988

Note: (a) The manipulation test was constructed using a 2nd-order local-polynomial density estimator, an MSE-optimal bandwidth choice coupled with robust bias-correction, and the corresponding jackknife SE estimator. (b) The RD estimates are based on the conventional RD estimator and the Robust biased-corrected RD estimator with and without restriction. (c) The test is constructed by using “rddensity” statistical package.

Table S28. Placebo cutoff

	Females Placebo cutoff at age 50		Placebo cutoff at age 59 for males and 54 for females		Placebo cutoff at age 61 for males and 56 for females	
Outcomes	estimate	p-value	estimate	p-value	estimate	p-value
Self-reported health	0.2627	0.1996	0.8070	0.4304	0.2627	0.1996
Life Satisfaction	0.0601	0.6502	0.1652	0.6186	0.0599	0.6534
CESD Scale	-1.2230	0.6686	-11.7595	0.1435	-1.2227	0.6682
MMSE	-1.3207	0.1826	0.8860	0.6832	-1.3138	0.1847
ADL	0.0386	0.9208	-0.4631	0.6063	0.0388	0.9188
IADL	-0.3019	0.1478	-0.7253	0.3883	-0.2978	0.1535
Total Recall Score	-1.2272	0.5864	5.4550	0.3121	-1.2205	0.5912

Table S29. Fuzzy RD sensitivity test to observations near cutoff: The Donut-Hole Approach

Donut-hole radius	MSE-optimal bandwidth	Estimate	Robust 95% CI	Excluded observations L|R
		**Self-reported Health**		
0	3.9	0.1374	[-0.2580, 0.6651]	0|0
0.1	4.4	0.1010	[-0.3399, 0.6815]	16|39
0.2	5.1	0.1014	[-0.2955, 0.7022]	27|48
0.3	3.5	-0.2648	[-1.5994, 1.0837]	118|115
		**Life Satisfaction**		
0	3.8	-0.0567	[-0.3100, 0.2671]	0|0
0.1	2.9	-0.0732	[-0.3523, 0.2905]	0|28
0.2	2.9	-0.0784	[-0.3322, 0.2522]	26|41
0.3	2.5	0.1074	[-0.2387, 0.5882]	109|112
		**CESD Scale**		
0	3.6	1.4545	[-4.8459, 7.3168]	0|0
0.1	2.9	1.0680	[-5.4796, 6.7111]	0|29
0.2	2.7	1.2889	[-4.8091, 6.7180]	26|43
0.3	2.4	-3.2049	[-12.0116, 4.1531]	110|115
		**MMSE**		
0	3.6	-0.4198	[-2.3391, 1.3704]	0|0
0.1	3.4	-0.4724	[-2.4416, 1.3340]	0|26
0.2	3.2	-0.4466	[-2.3756, 1.3820]	25|38
0.3	2.4	1.8145	[-0.7288, 5.0330]	102|103
		**ADL**		
0	4	0.0404	[-0.4302, 0.5152]	0|0
0.1	3.6	-0.0986	[-0.5897, 0.3324]	0|29
0.2	2.9	-0.2246	[-0.7626, 0.1943]	28|44
0.3	2.0	-0.5259	[-1.7930, 0.5058]	120|122
		**IADL**		
0	2.8	-0.1869	[-0.8337, 0.2795]	0|0
0.1	3.9	-0.0739	[-0.5574, 0.3672]	0|27
0.2	3.2	-0.0997	[-0.6271, 0.3768]	28|41
0.3	1.9	-0.5925	[-2.0847, 0.6805]	107|116
		**Total Recall Score**		
0	3.9	2.2990	[-1.0749, 7.1681]	0|0
0.1	3.0	2.2326	[-2.0861, 7.2232]	0|26
0.2	2.7	3.5337	[-0.5950, 8.8061]	26|39
0.3	2.4	8.9991	[1.3692, 20.6469]	106|108

Fig. S1 Cutoff age for female employees

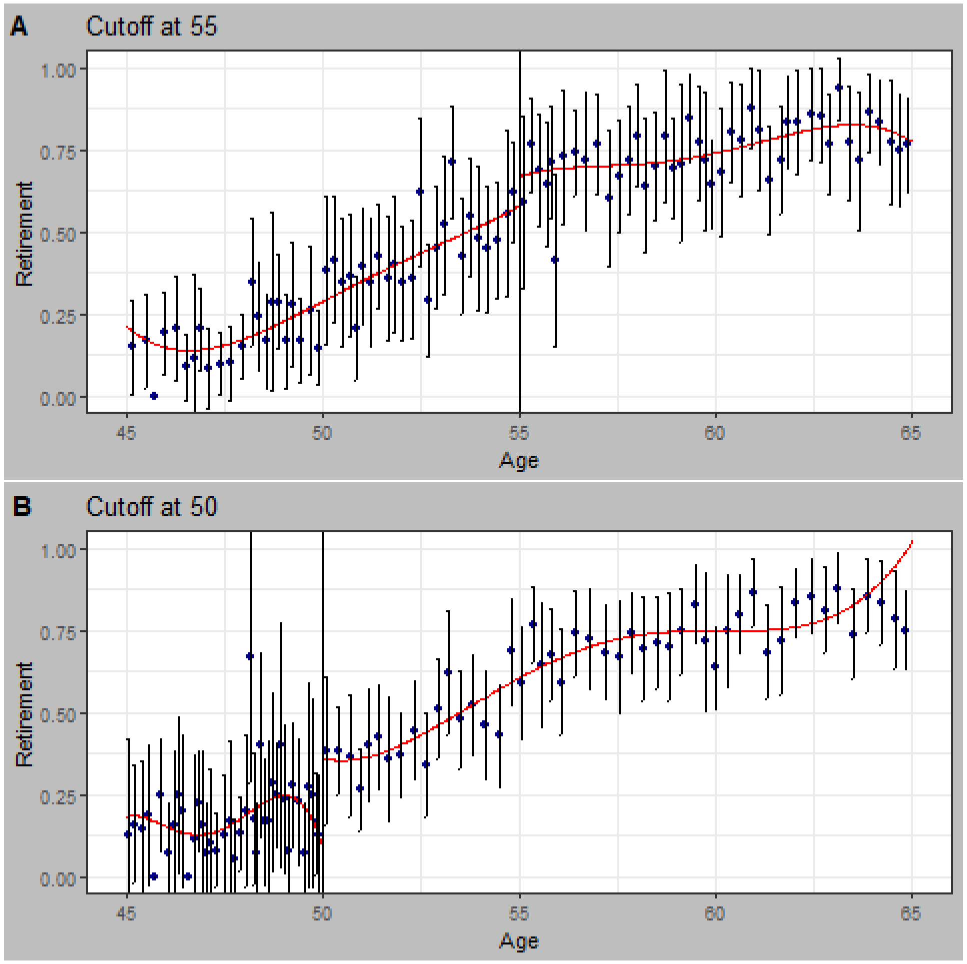

Note: We used the mimicking variance quantile-spaced method to estimate the sample means.

Fig. S2 Self-reported Health RD Plot
Integrated Mean Square Error (IMSE) approach vs. Mimicking Variance (MV) approach

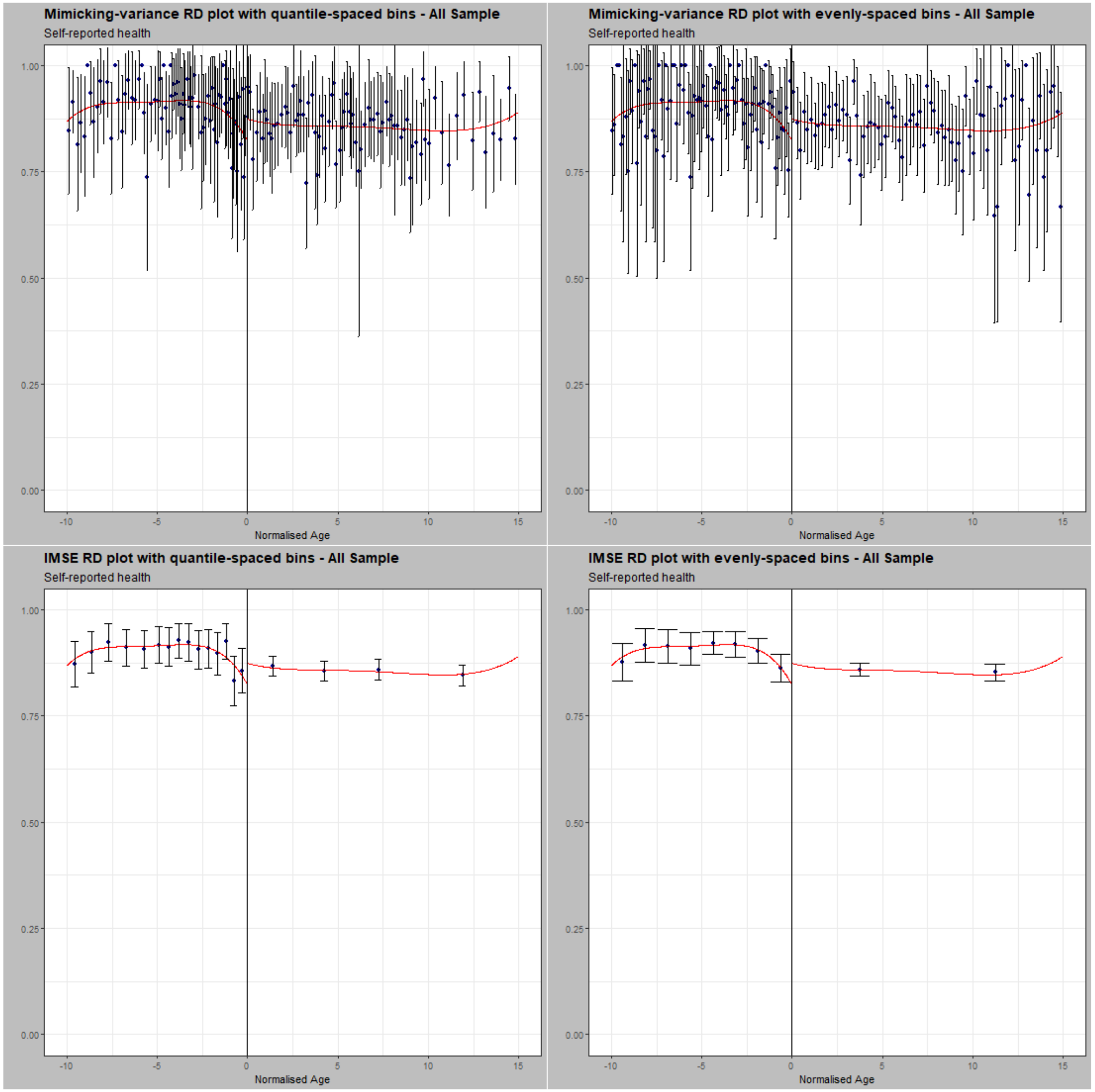

Fig. S3 Life Satisfaction RD Plot
Integrated Mean Square Error (IMSE) approach vs. Mimicking Variance (MV) approach

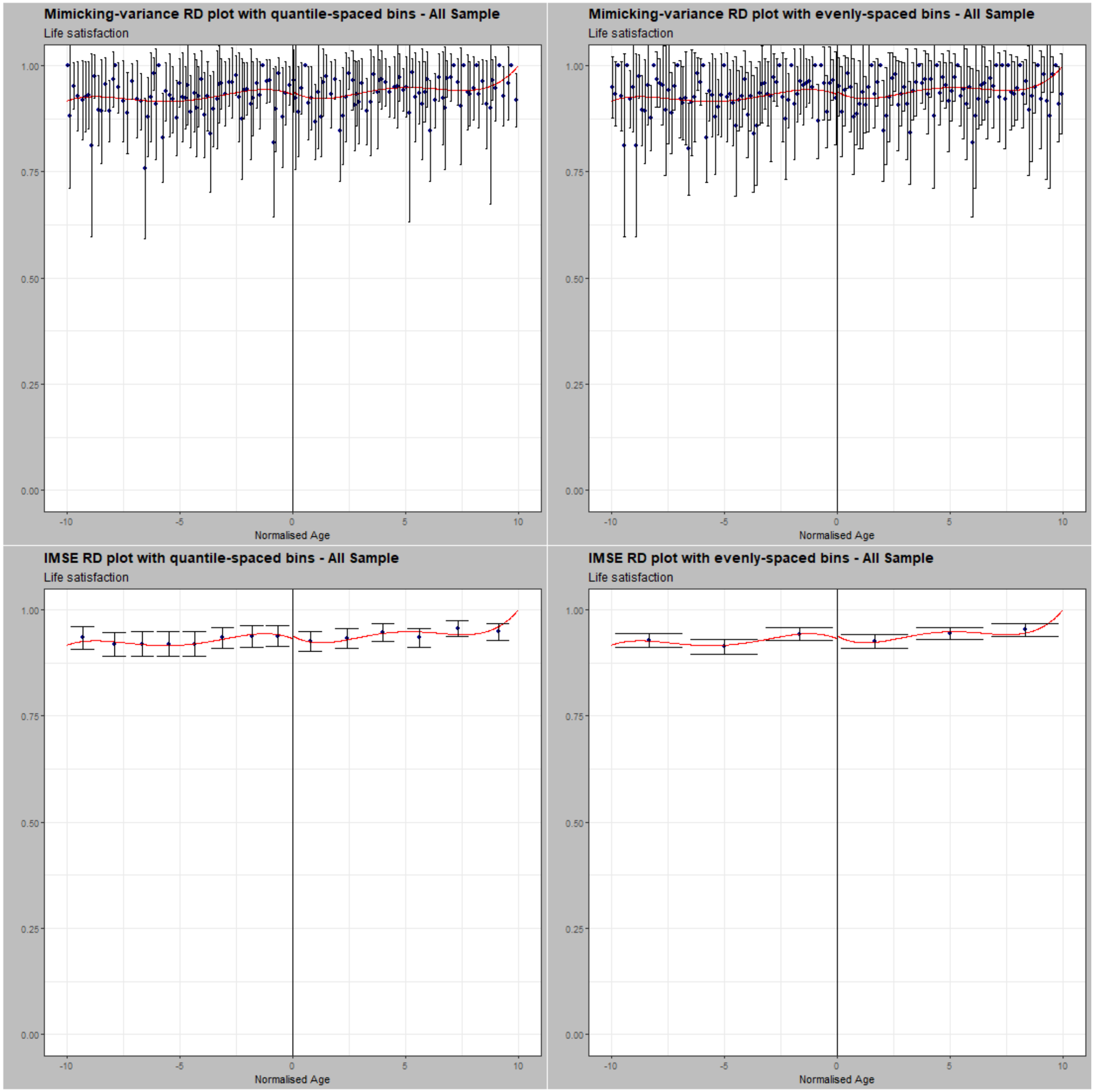

Fig. S4 CESD Scale RD Plot
Integrated Mean Square Error (IMSE) approach vs. Mimicking Variance (MV) approach

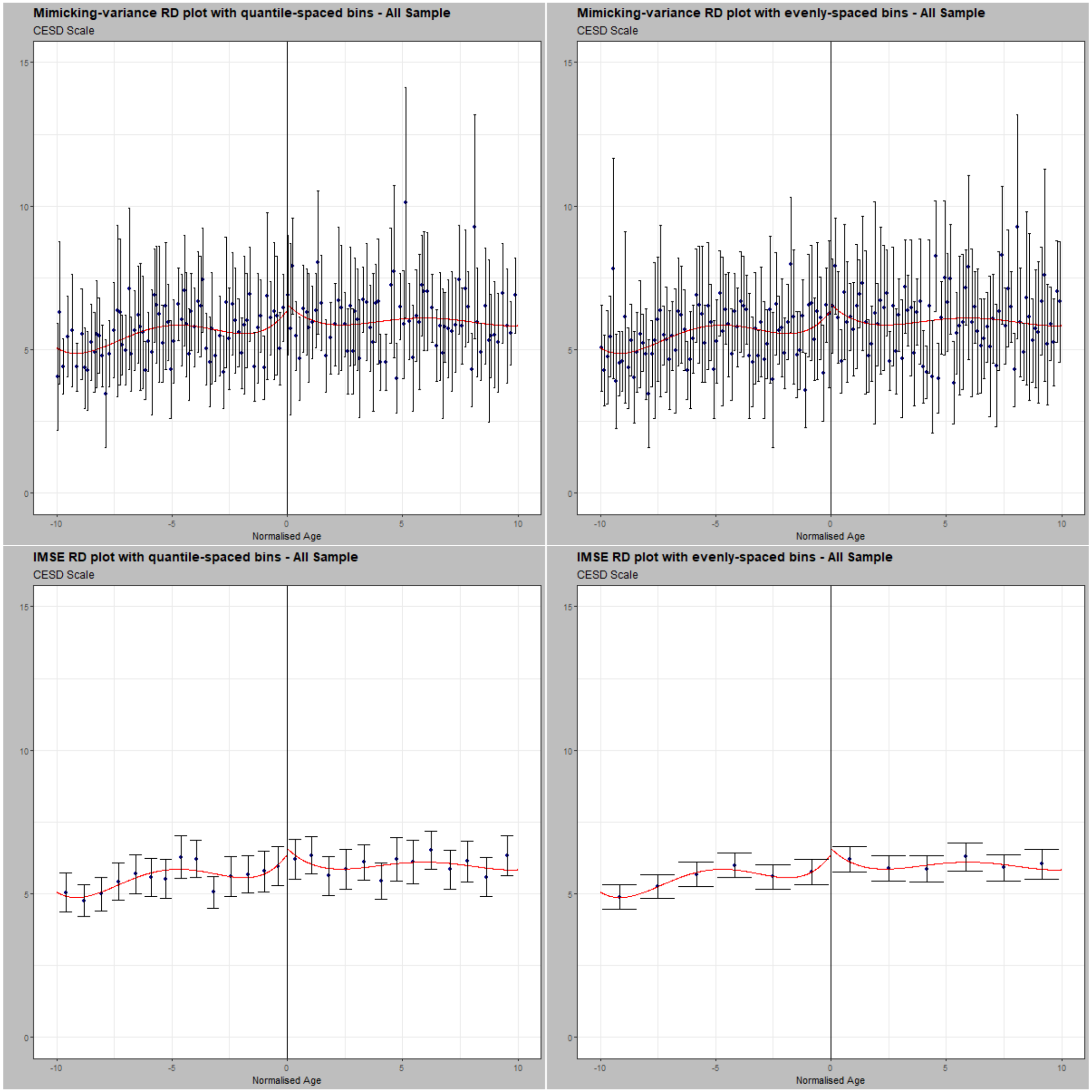

Fig. S5 MMSE RD Plot
Integrated Mean Square Error (IMSE) approach vs. Mimicking Variance (MV) approach

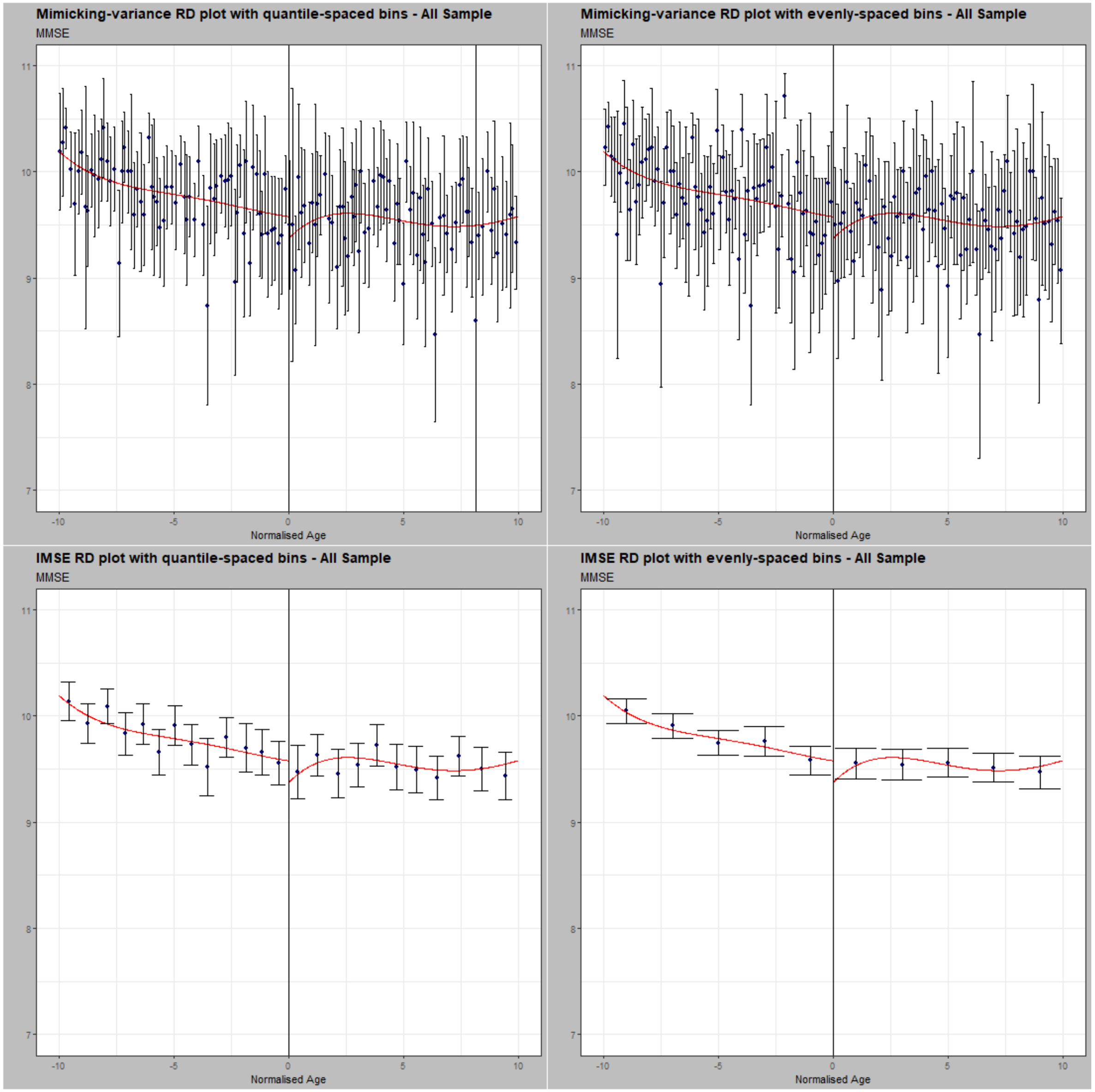

Fig. S6 ADL RD Plot
Integrated Mean Square Error (IMSE) approach vs. Mimicking Variance (MV) approach

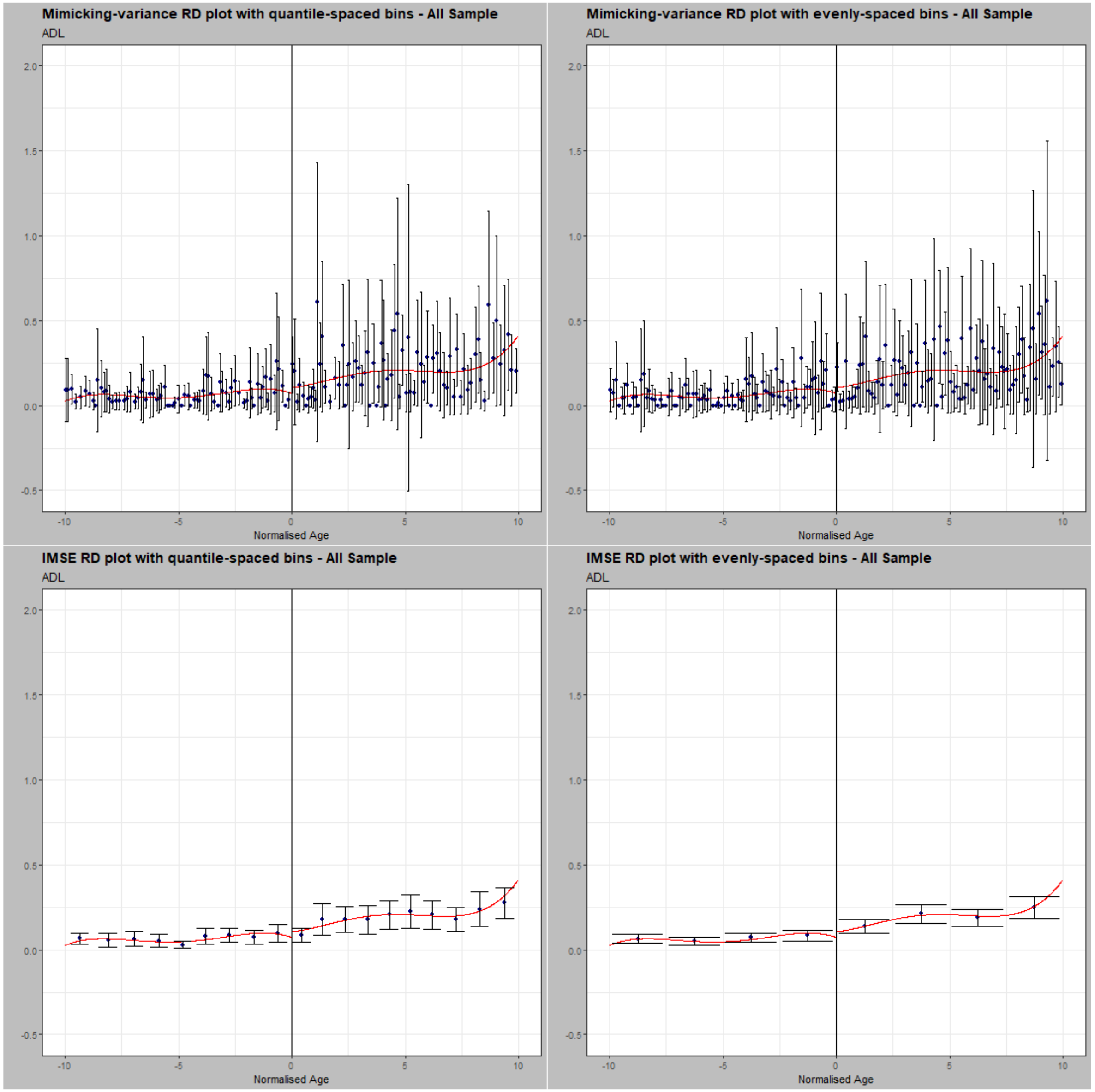

Fig. S7 IADL RD Plot

Integrated Mean Square Error (IMSE) approach vs. Mimicking Variance (MV) approach

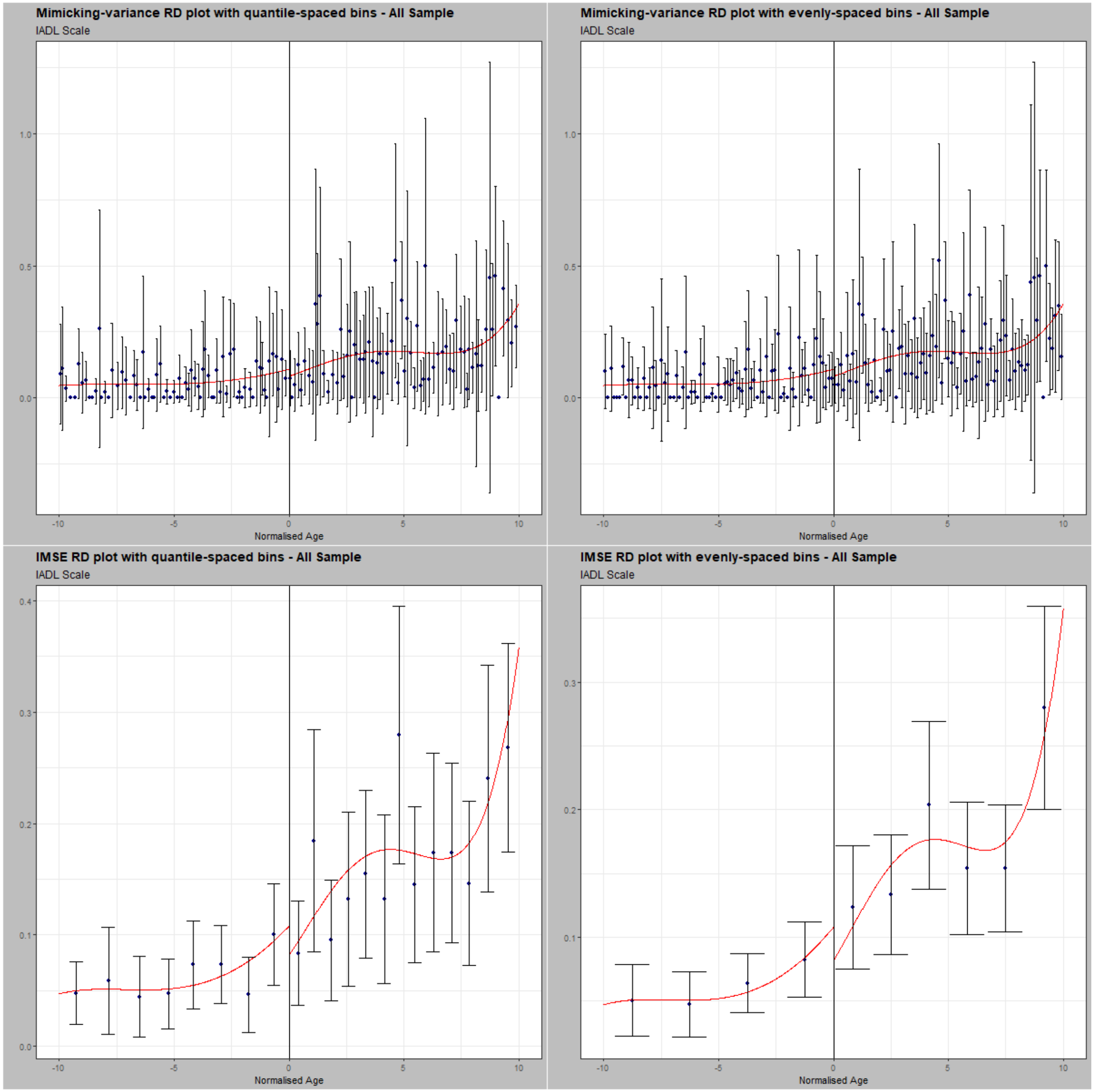

Fig. S8 Total Recall Score RD Plot
Integrated Mean Square Error (IMSE) approach vs. Mimicking Variance (MV) approach

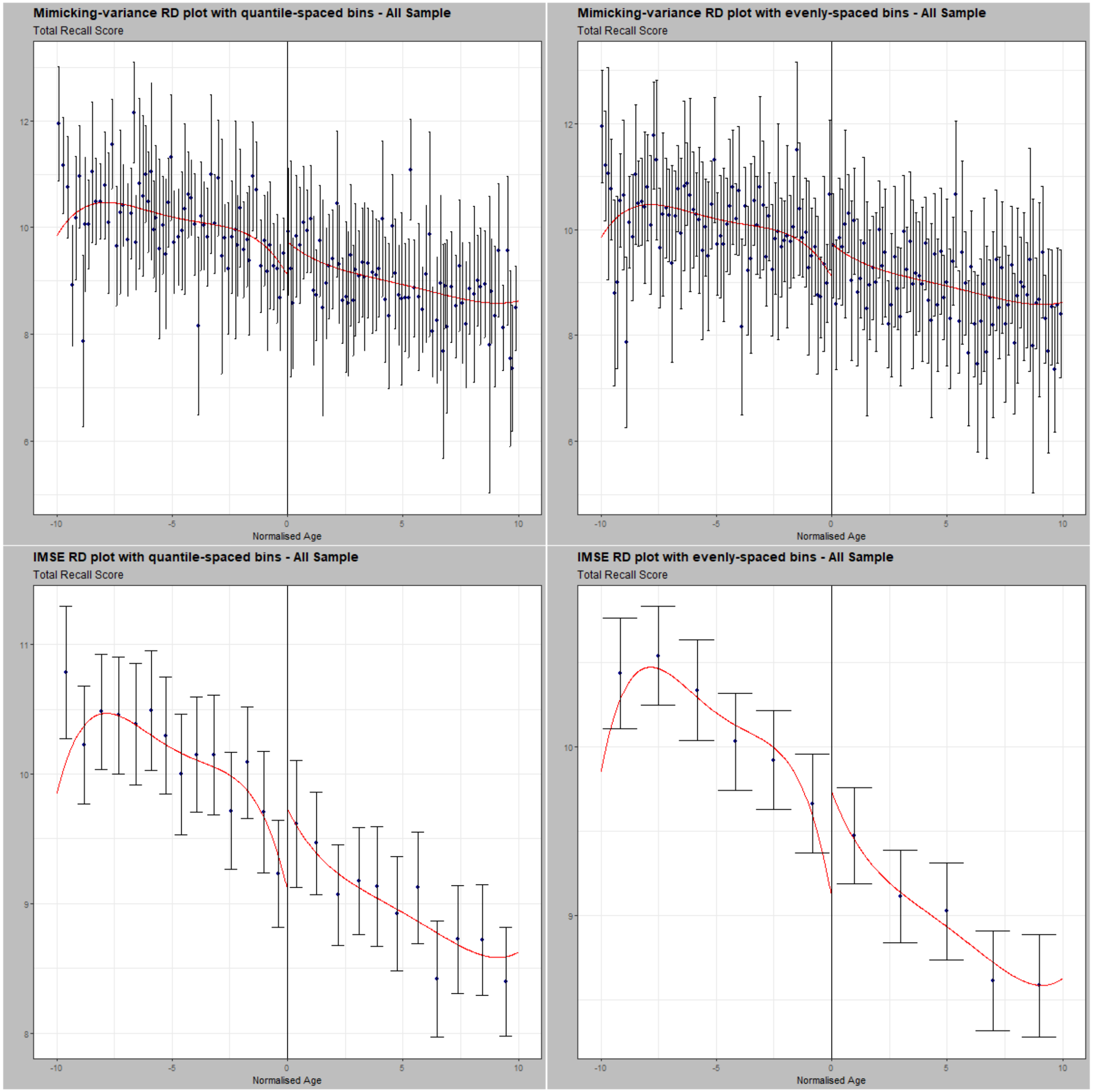

Fig. S9 Nonparametric Regression discontinuity robustness of bandwidth choice for self-reported health

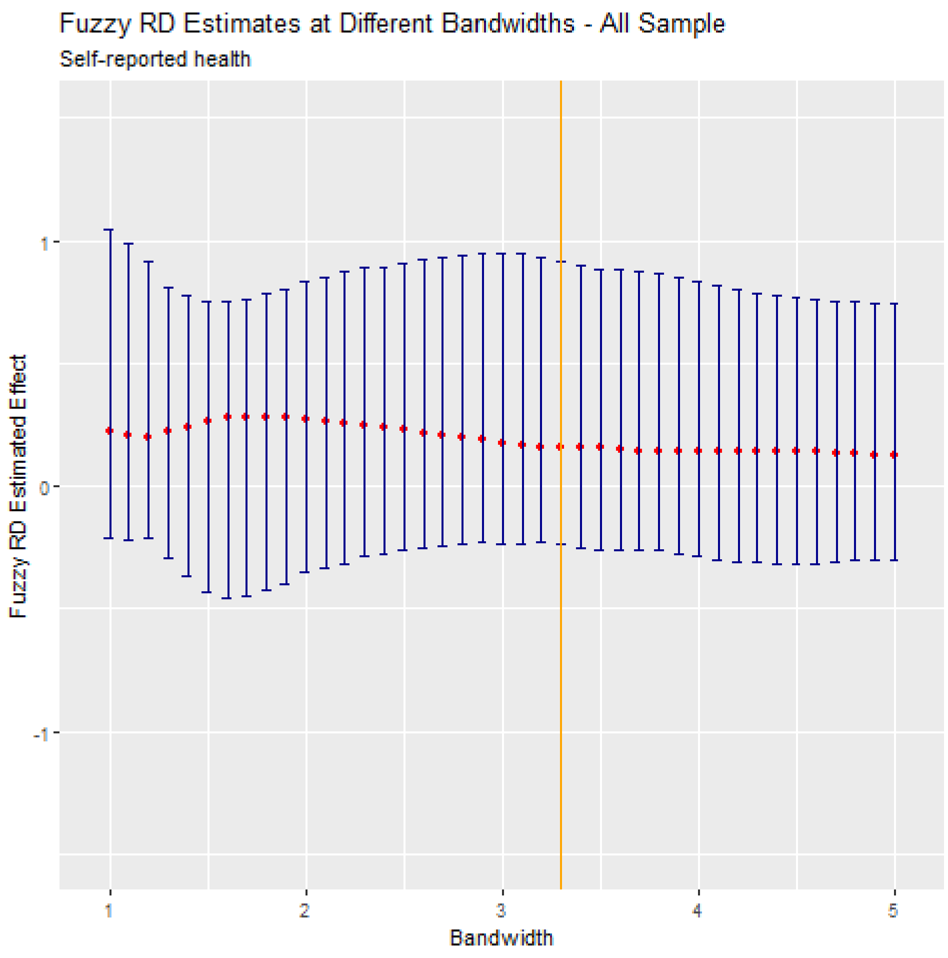

Fig. S10 Nonparametric Regression discontinuity robustness of bandwidth choice for life satisfaction

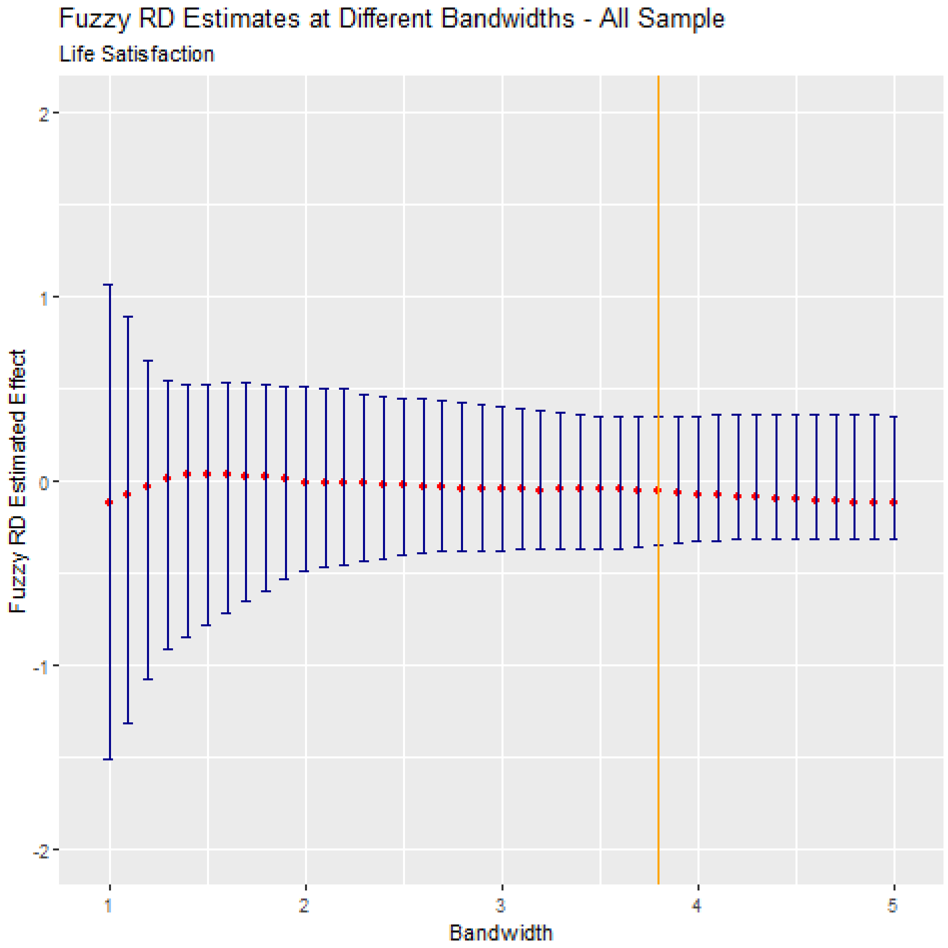

Fig. S11 Nonparametric Regression discontinuity robustness of bandwidth choice for CESD Scale

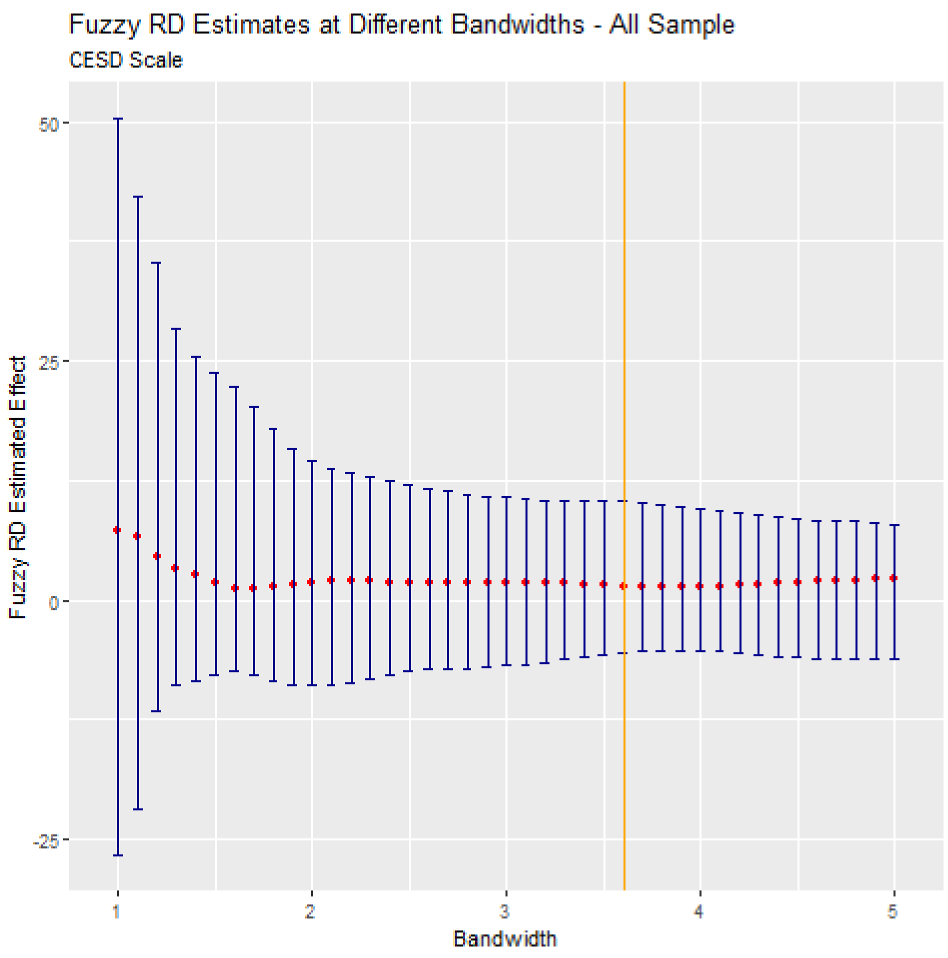

Fig. S12 Nonparametric Regression discontinuity robustness of bandwidth choice for MMSE

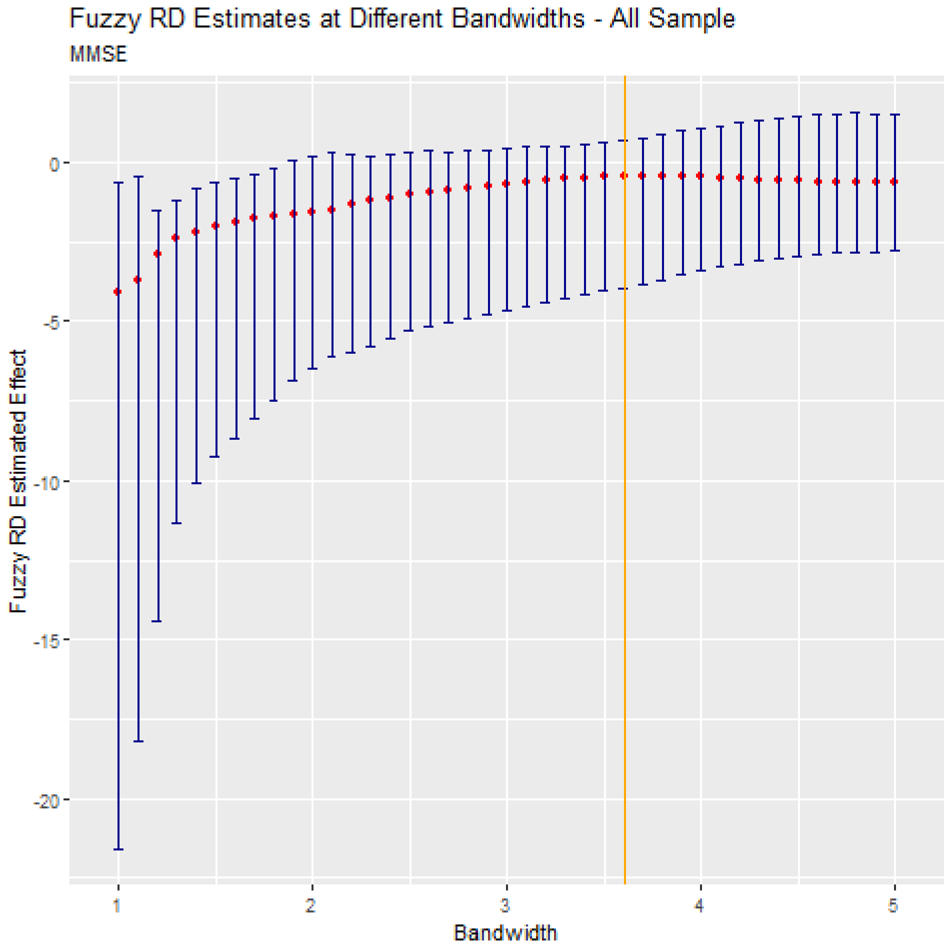

Fig. S13 Nonparametric Regression discontinuity robustness of bandwidth choice for ADL

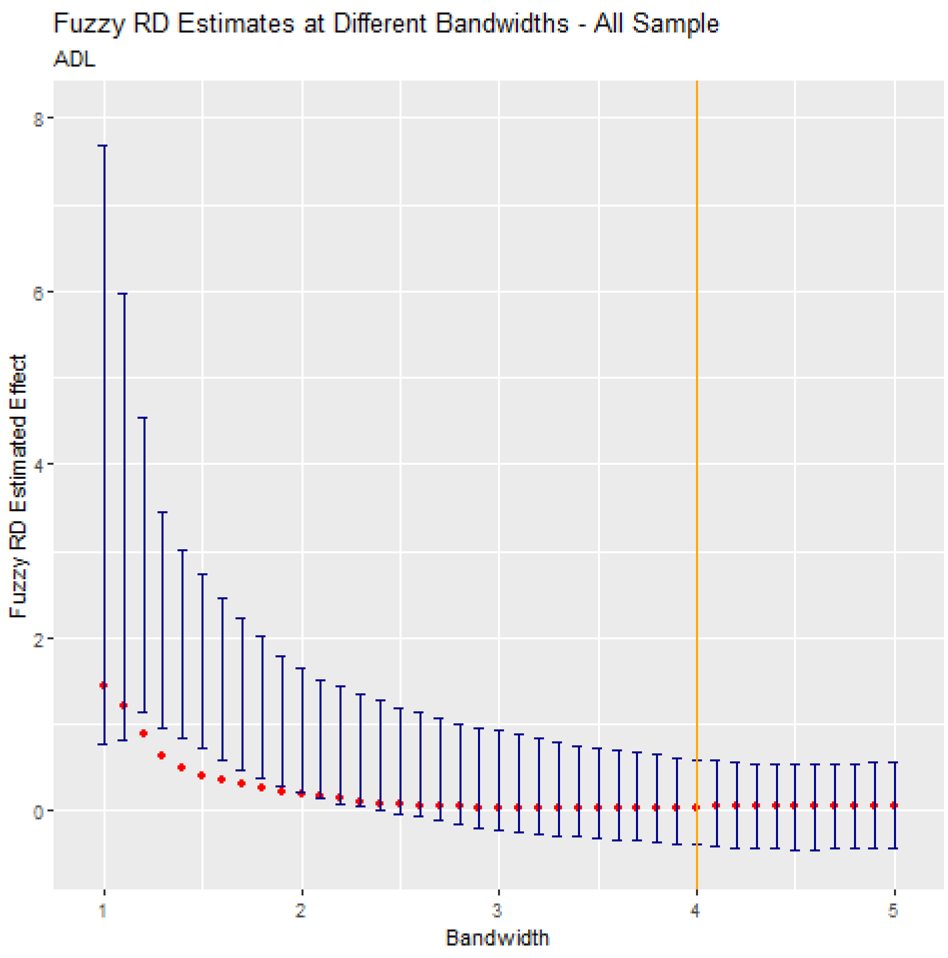

Fig. S14 Nonparametric Regression discontinuity robustness of bandwidth choice for IADL

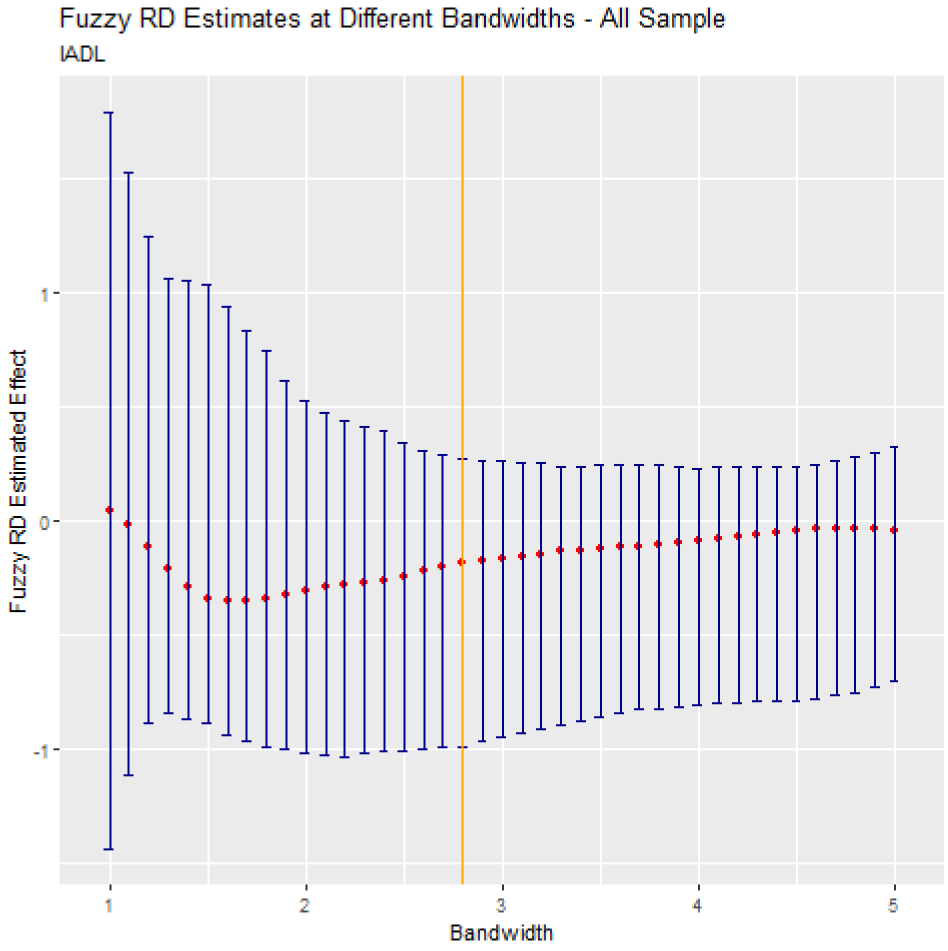

Fig S15 Nonparametric Regression discontinuity robustness of bandwidth choice for total recall score

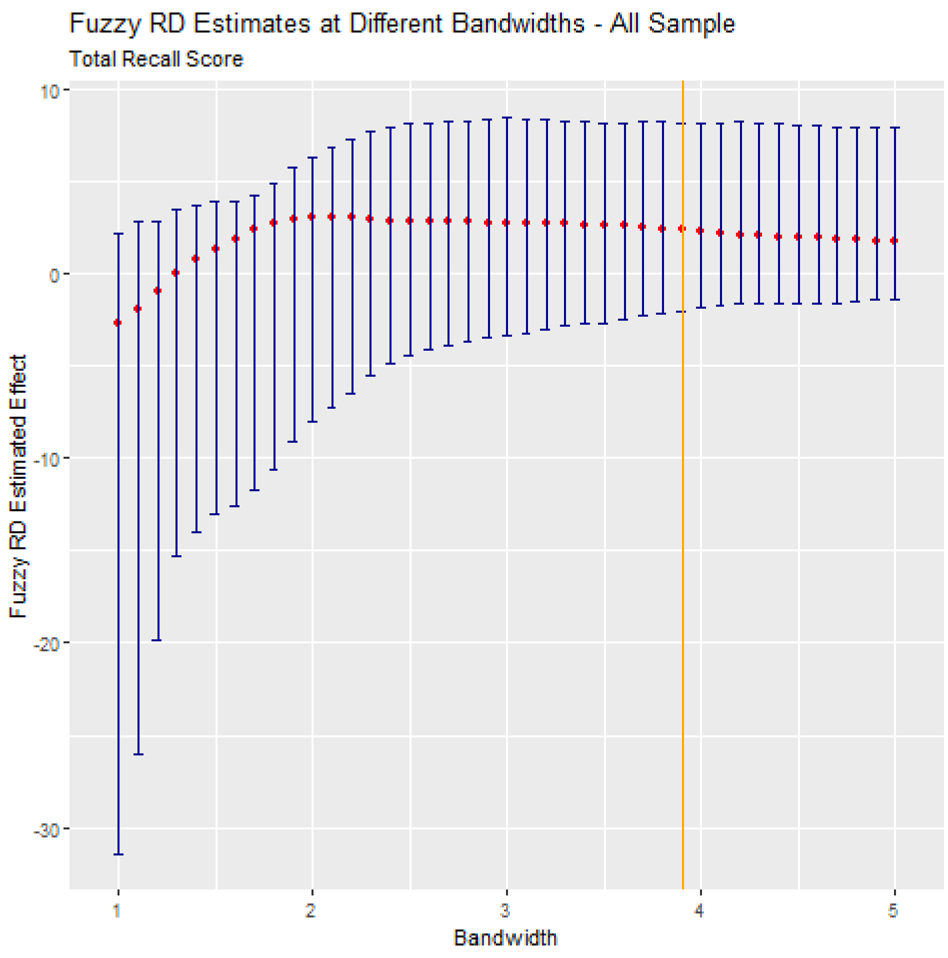

Fig. S16 Self-reported Health RD Plot by Gender

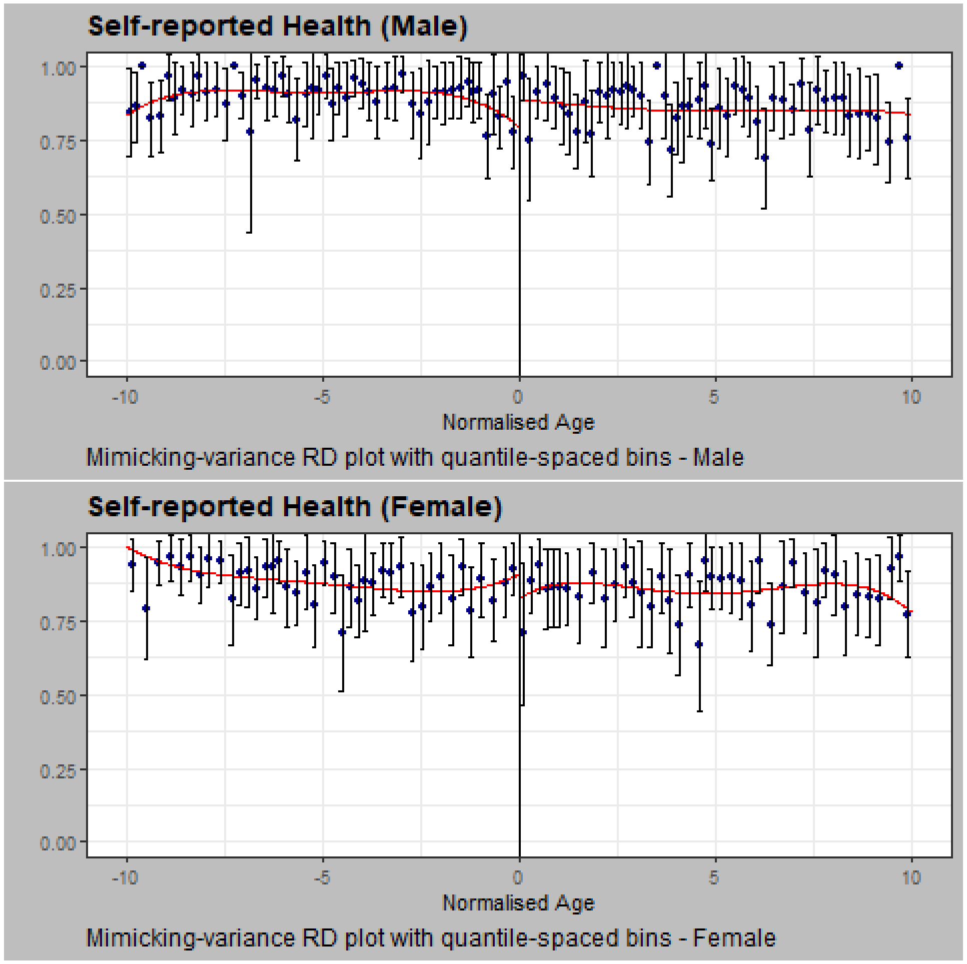

Fig. S17 Life Satisfaction RD Plot by Gender

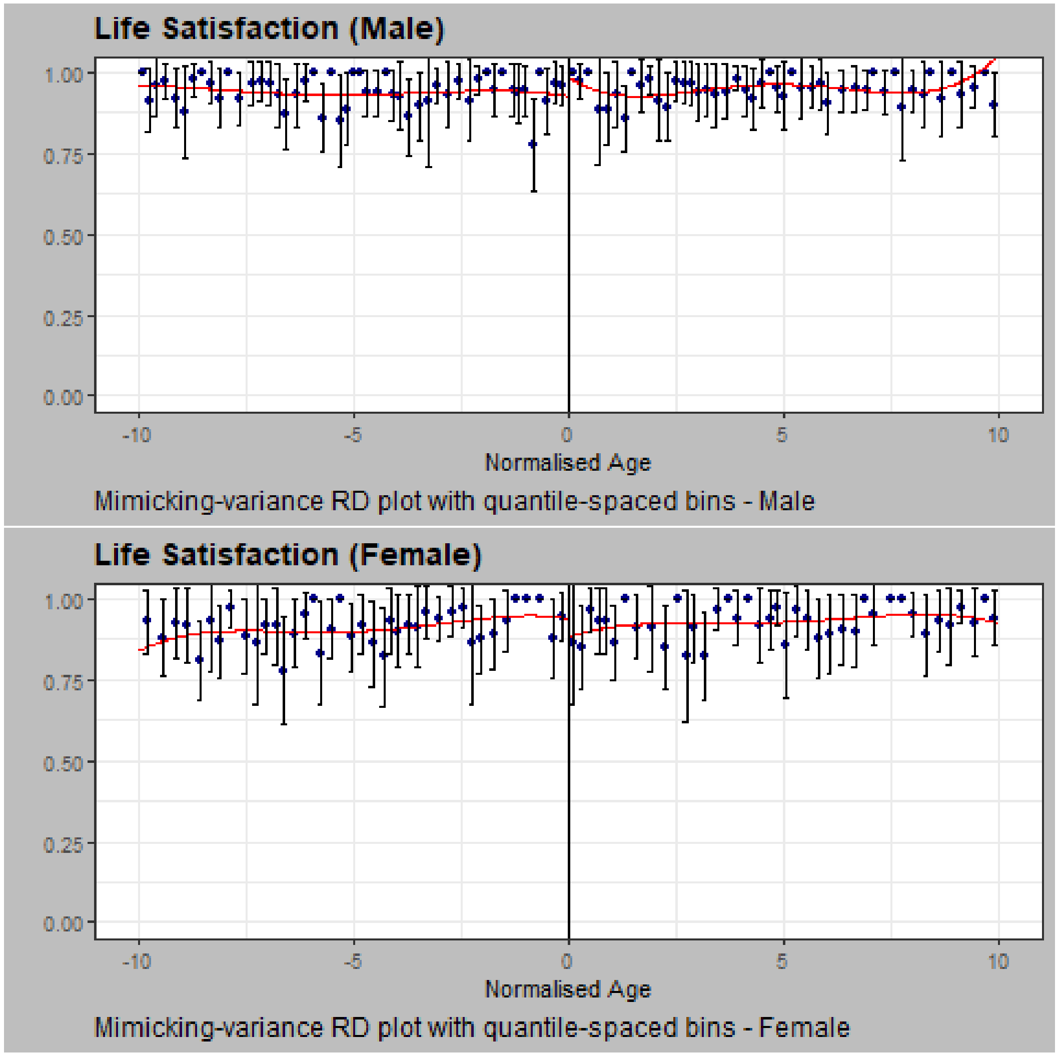

Fig. S18 CESD Scale RD Plot by Gender

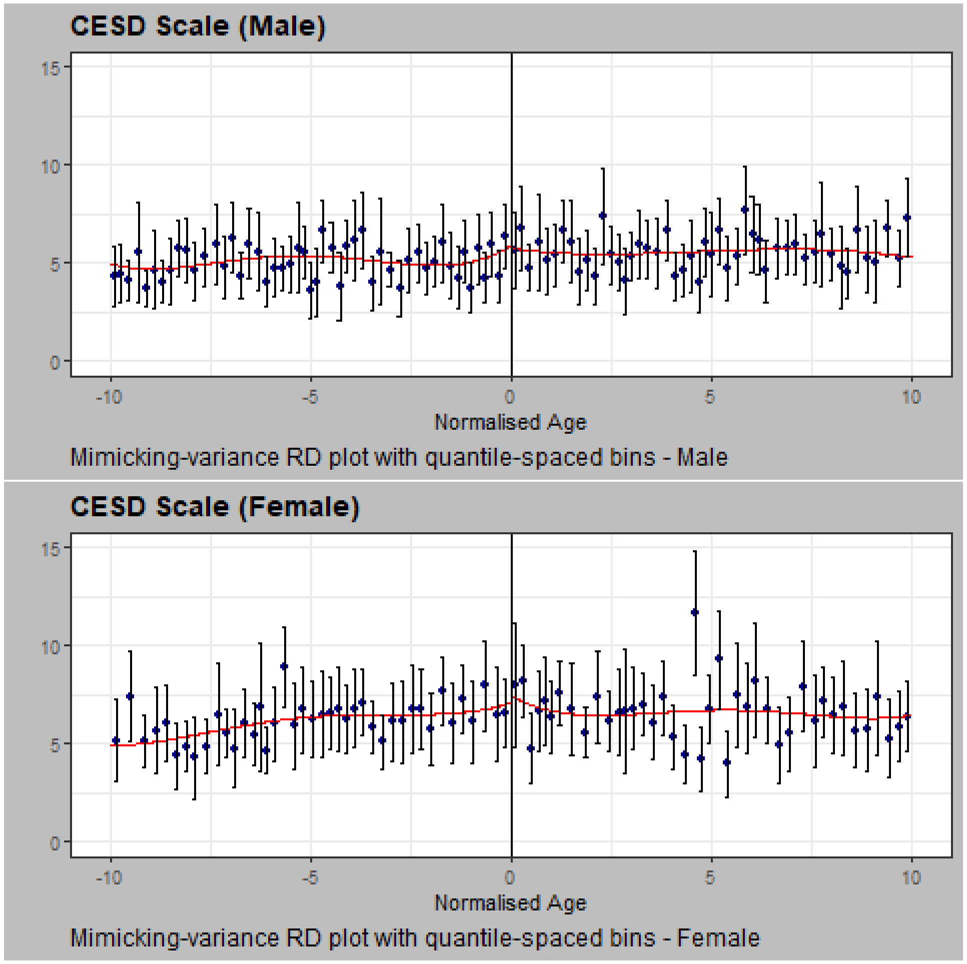

Fig. S19 MMSE RD Plot by Gender

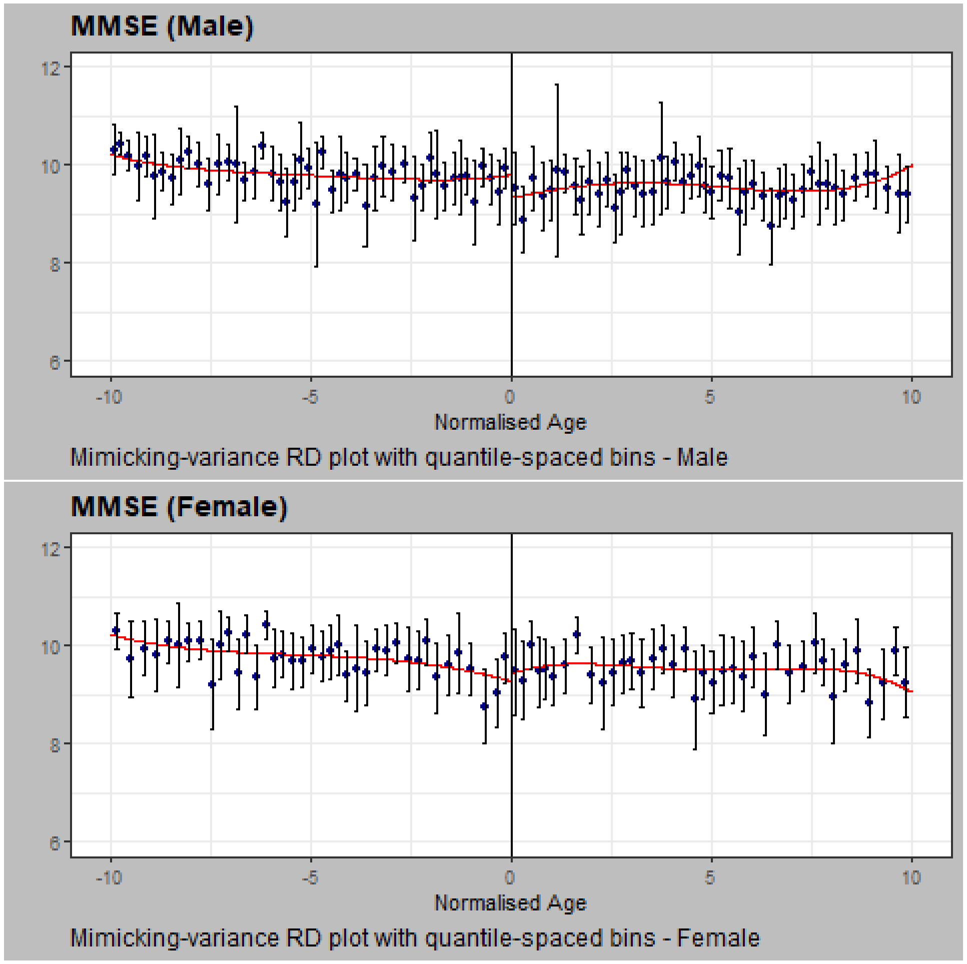

Fig. S20 ADL RD Plot by Gender

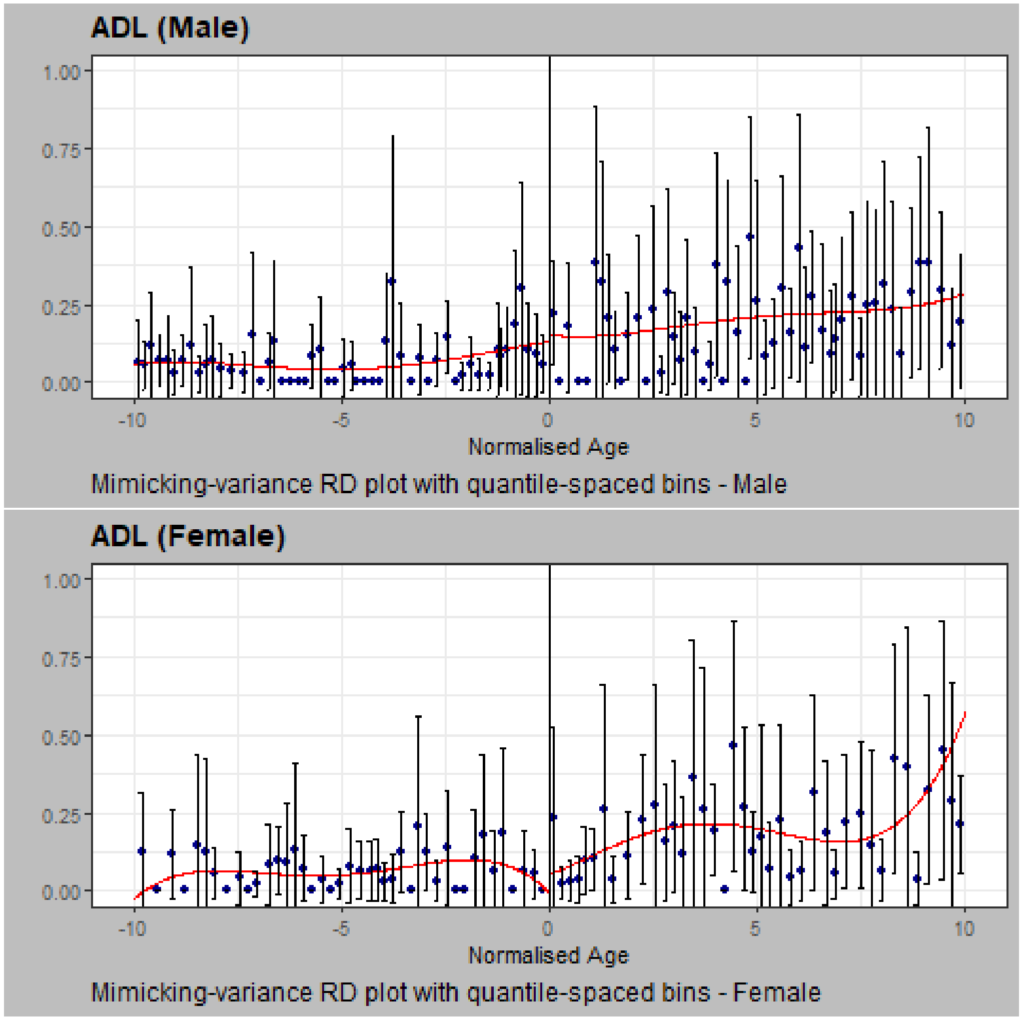

Fig. S21 IADL RD Plot by Gender

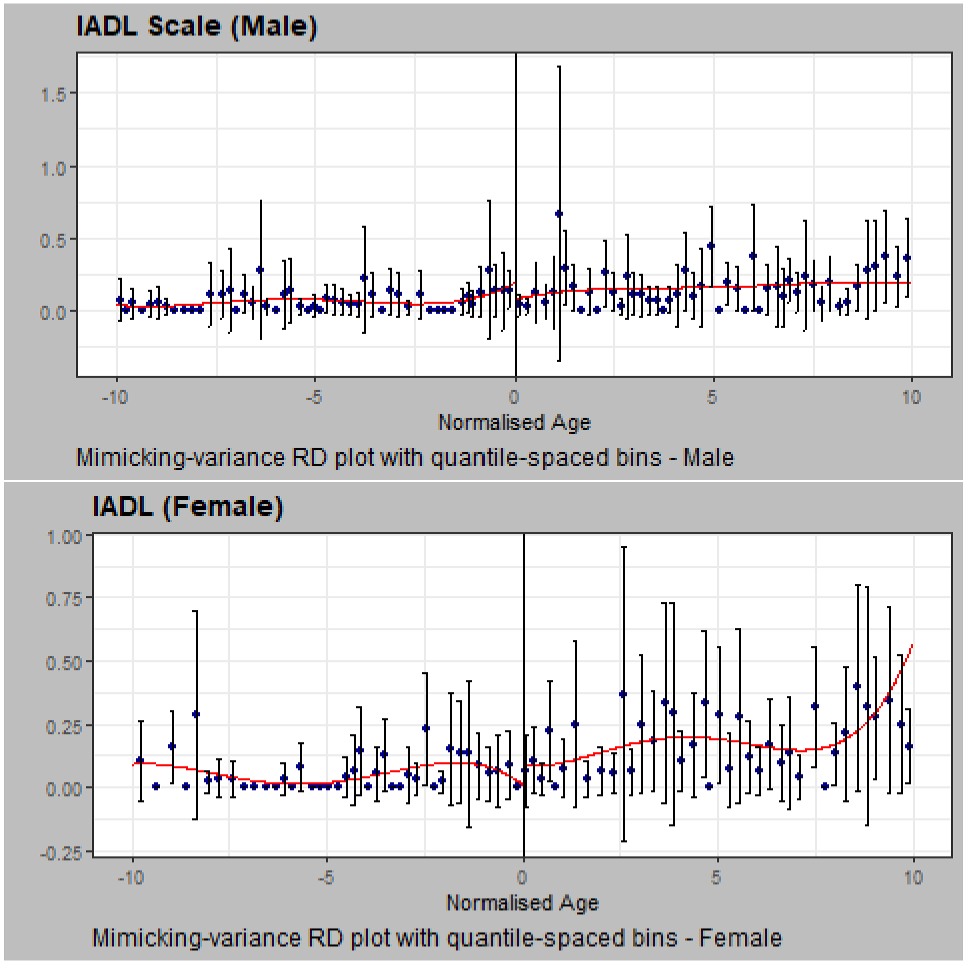

Fig. S22 Total Recall Score RD Plot by Gender

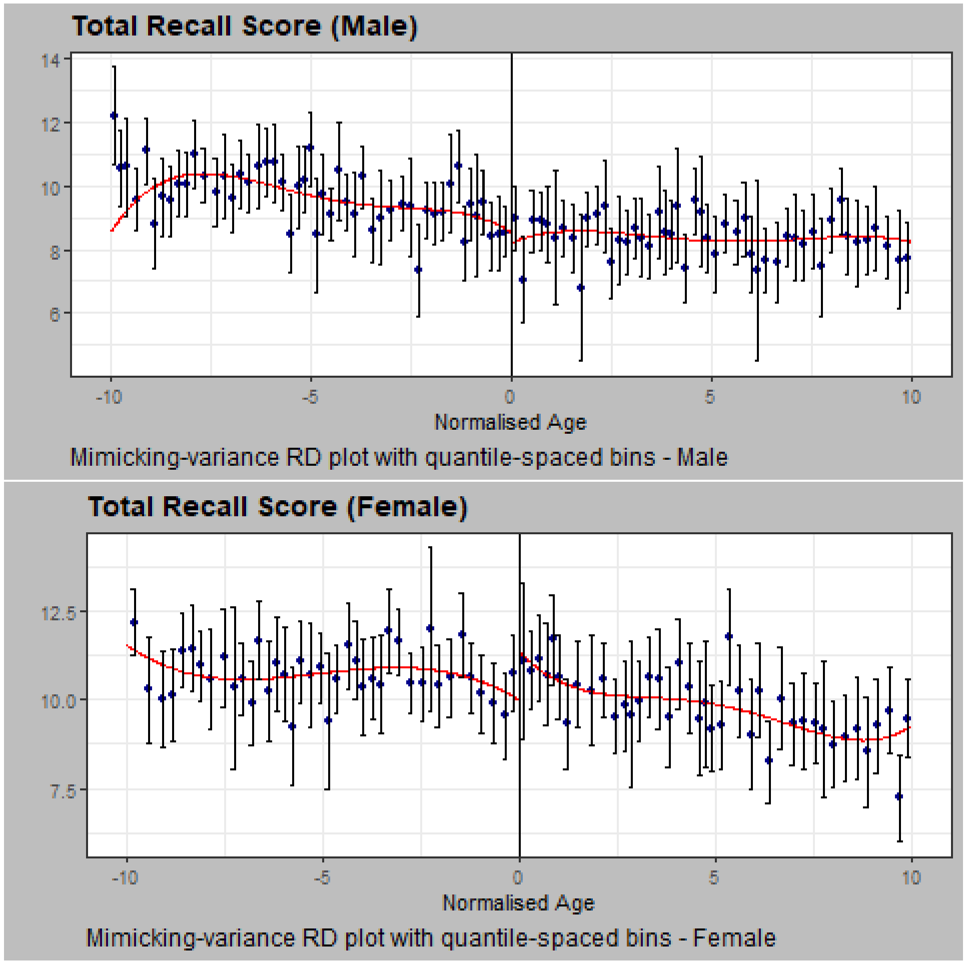

Fig. S23 Nonparametric Regression discontinuity robustness of bandwidth choice for self-reported health by gender

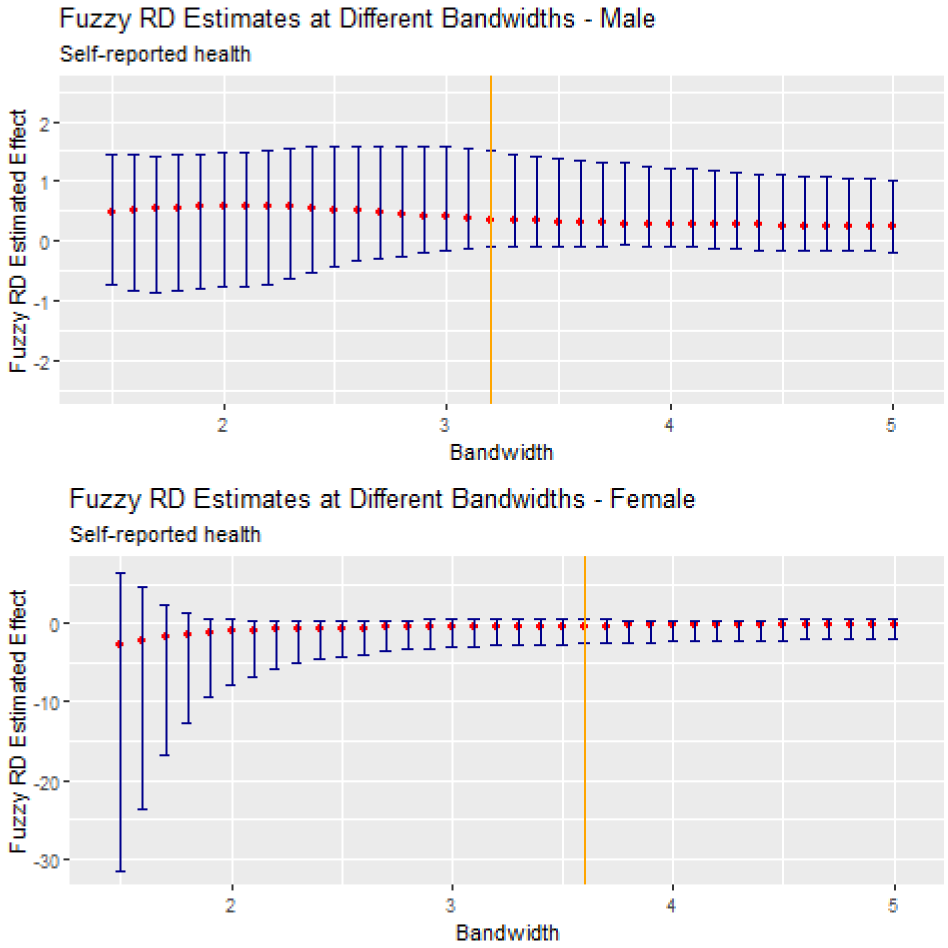

Fig. S24 Nonparametric Regression discontinuity robustness of bandwidth choice for life satisfaction by gender

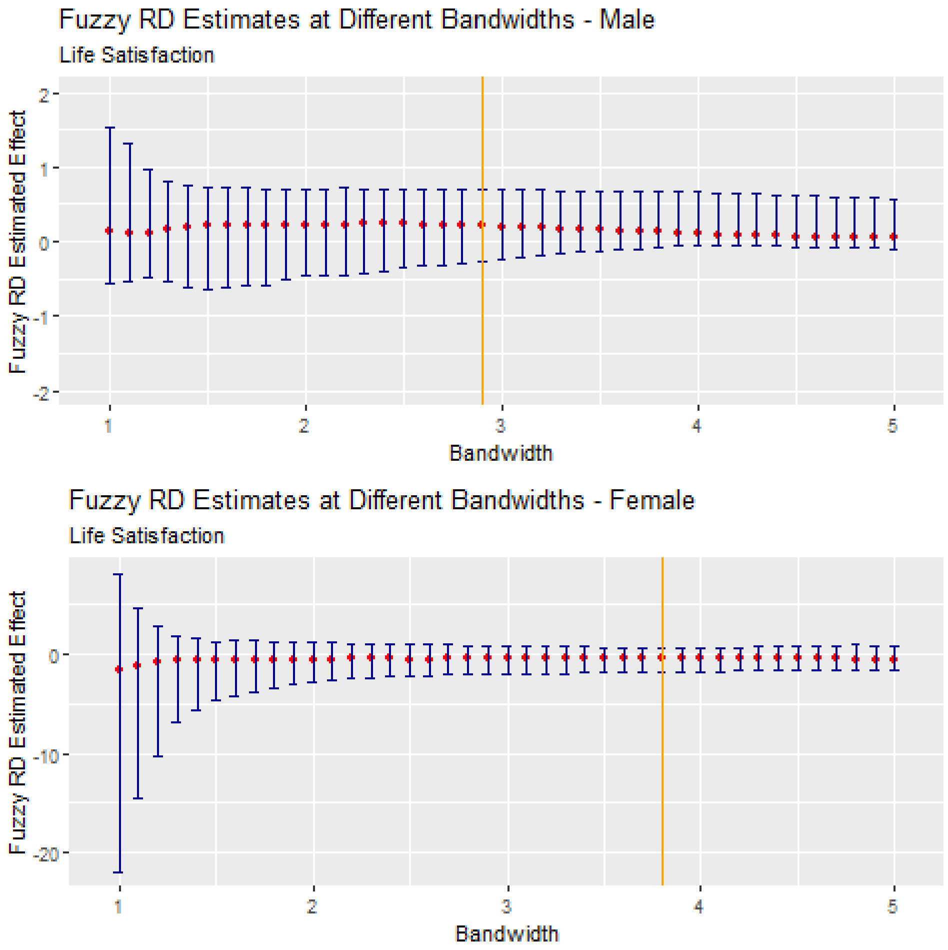

Fig. S25 Nonparametric Regression discontinuity robustness of bandwidth choice for CESD scale by gender

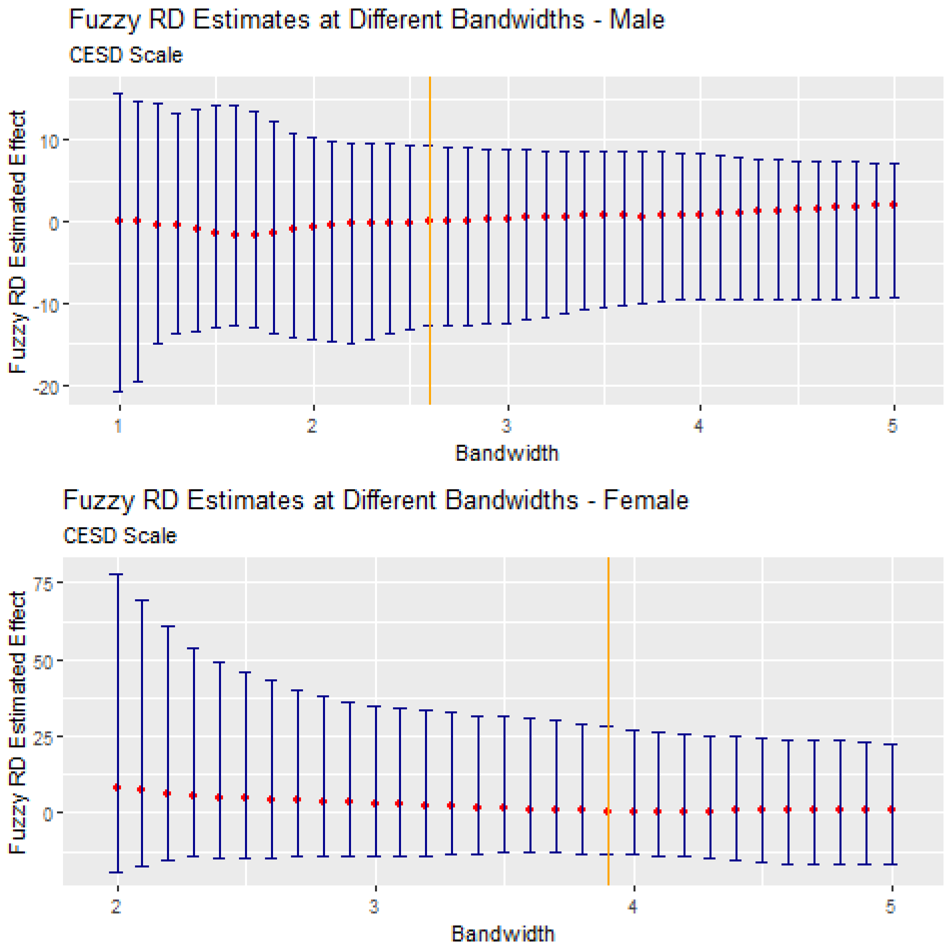

Fig. S26 Nonparametric Regression discontinuity robustness of bandwidth choice for MMSE by gender

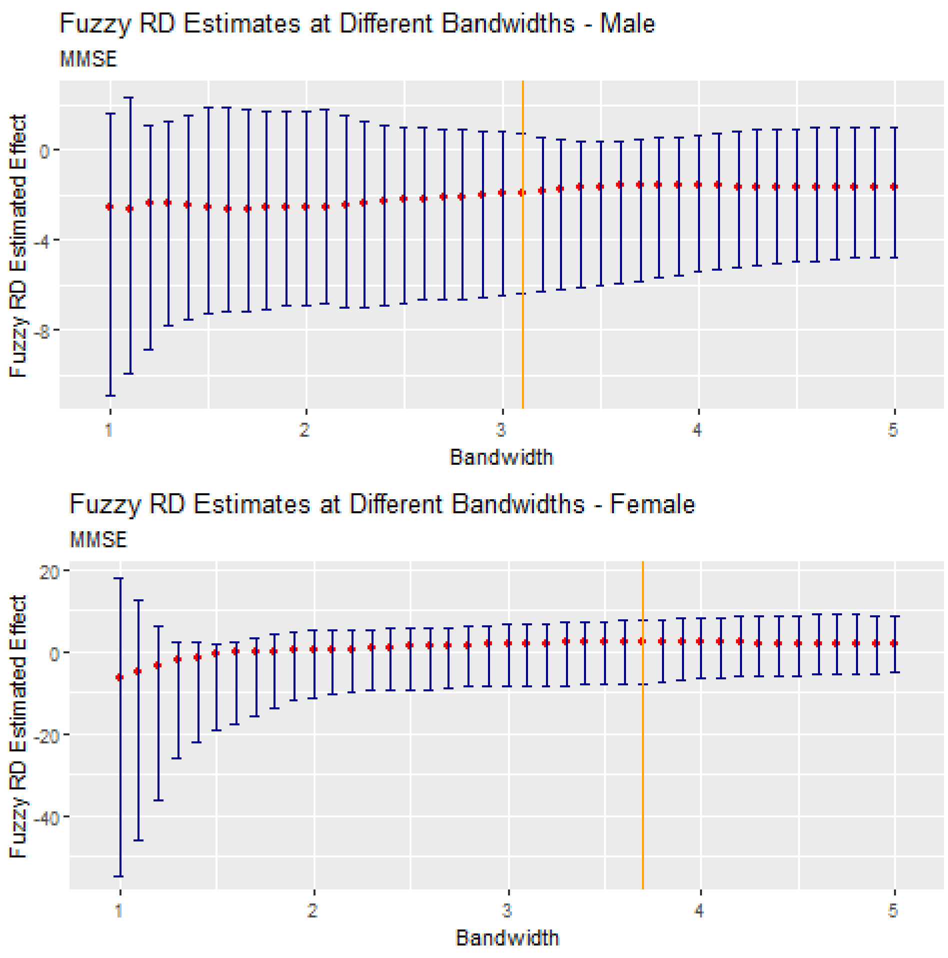

Fig. S27 Nonparametric Regression discontinuity robustness of bandwidth choice for ADL by gender

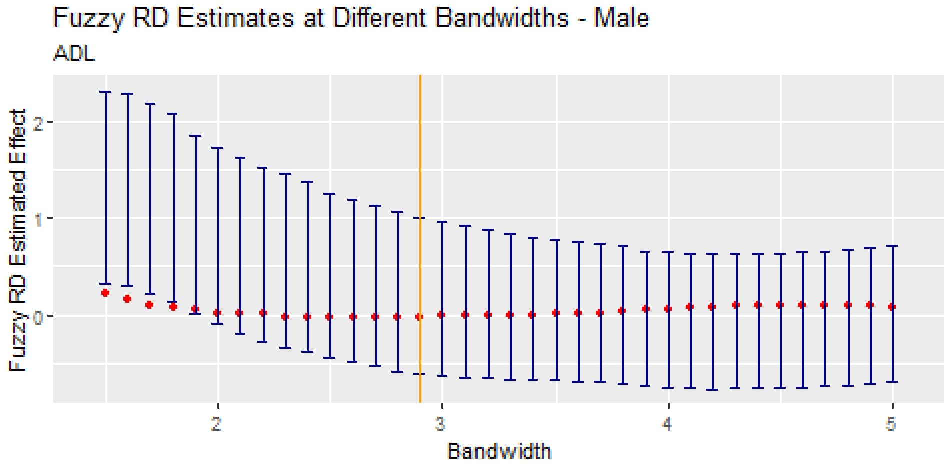

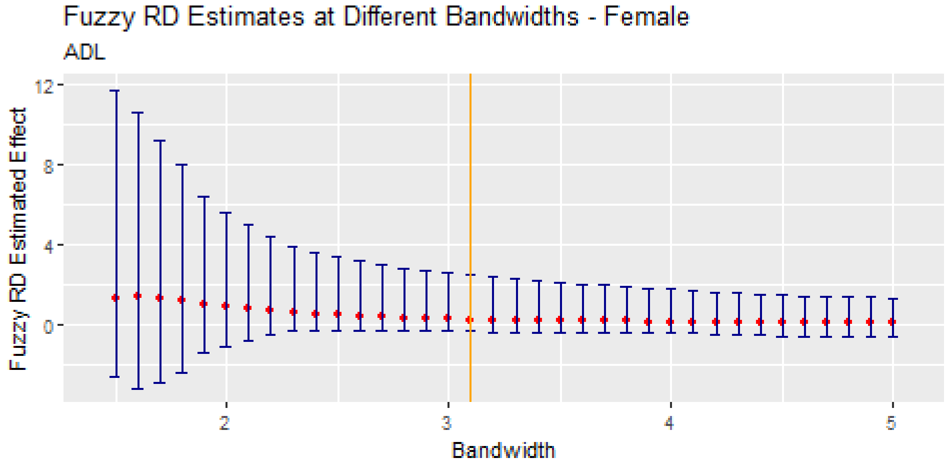

Fig. S28 Nonparametric Regression discontinuity robustness of bandwidth choice for IADL by gender

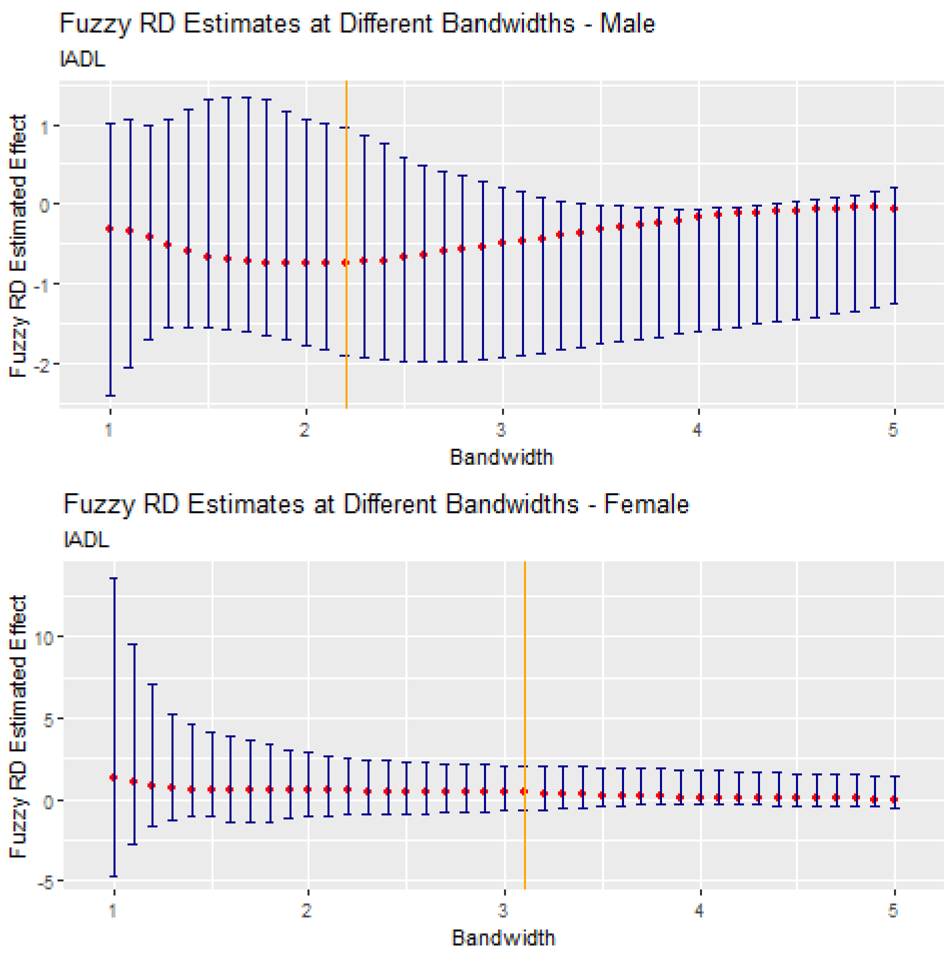

Fig. S29 Nonparametric Regression discontinuity robustness of bandwidth choice for Total recall score by gender

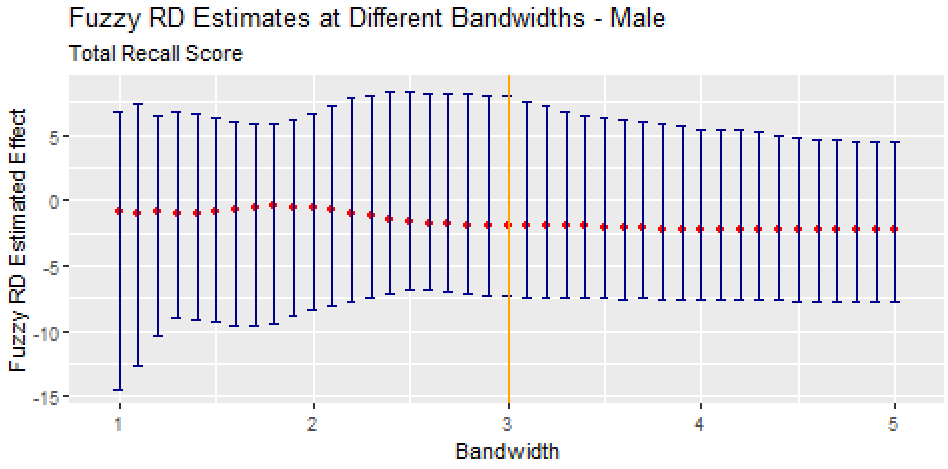

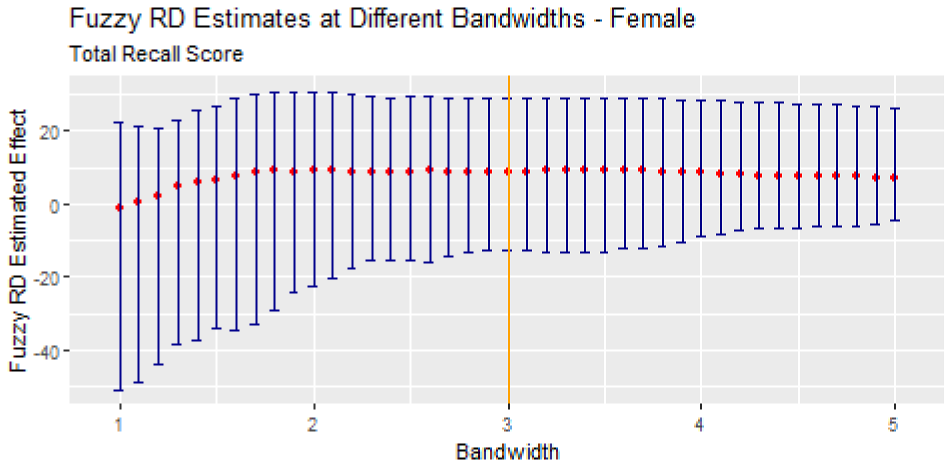

Fig. S30 Self-reported Health RD Plot by Education

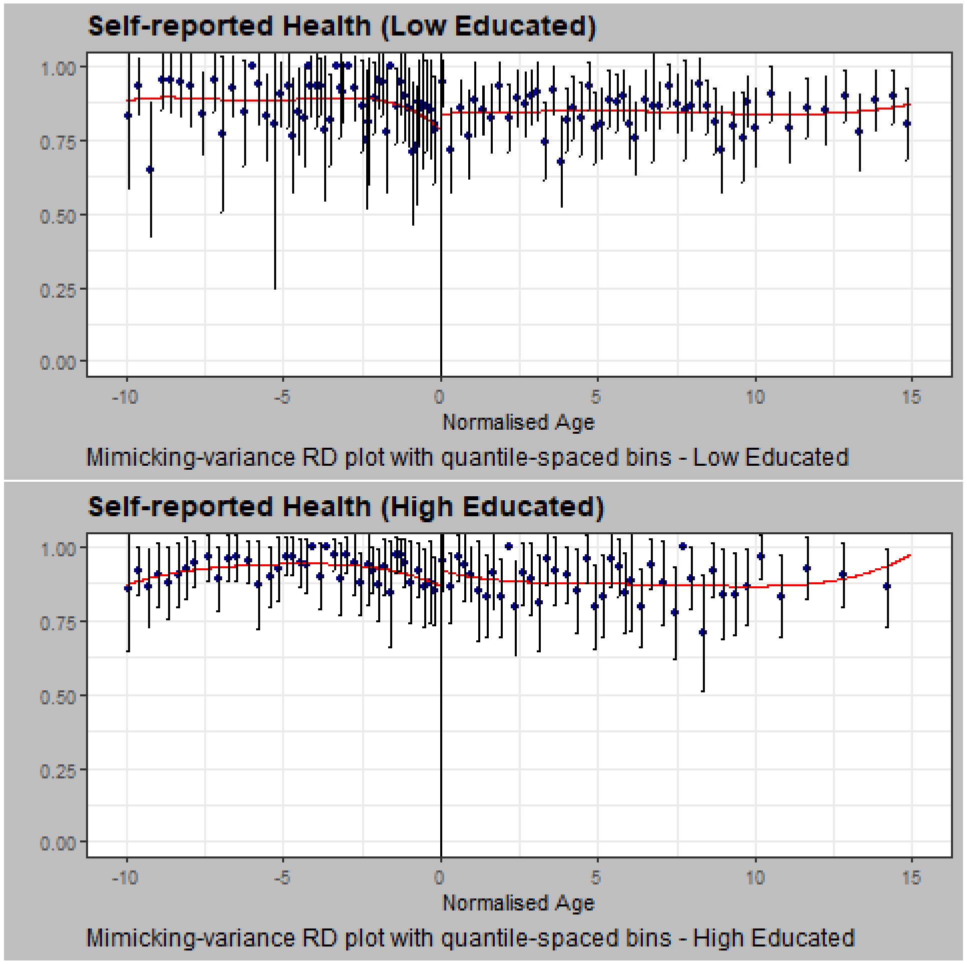

Fig. S31 Life Satisfaction RD Plot by Education

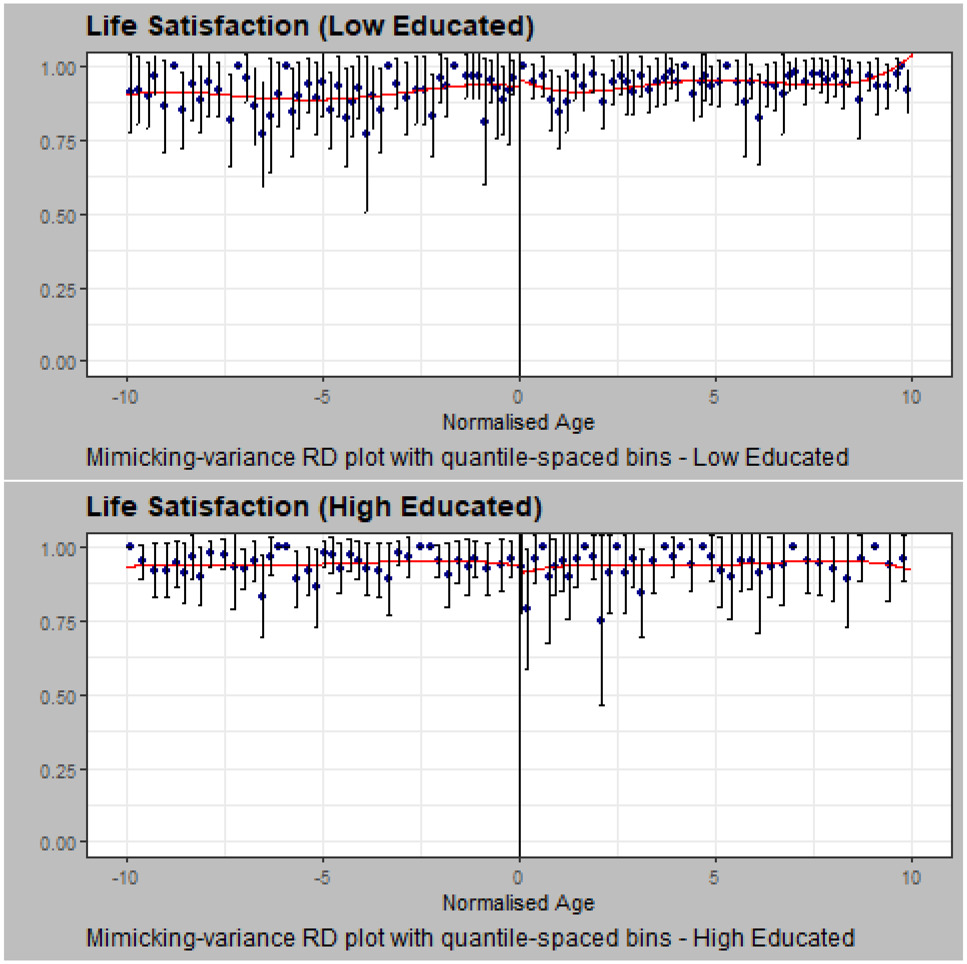

Fig. S32 CESD Scale RD Plot by Education

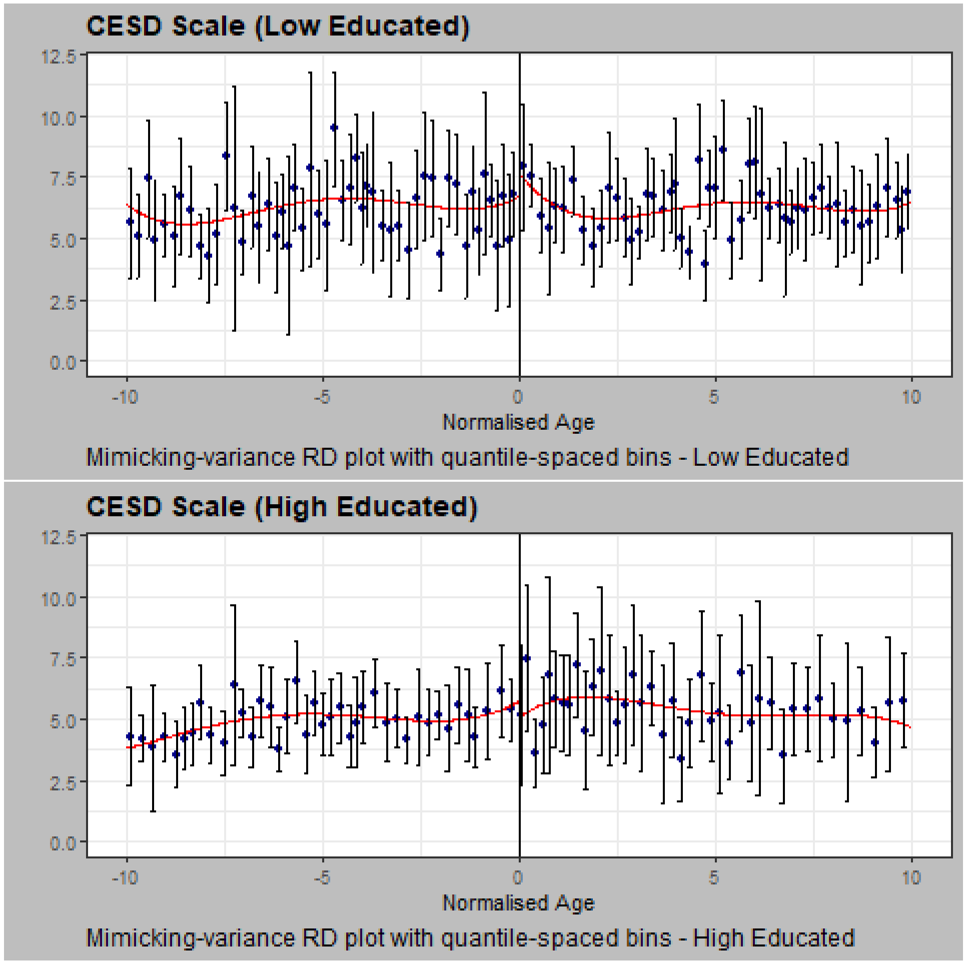

Fig. S33 MMSE RD Plot by Education

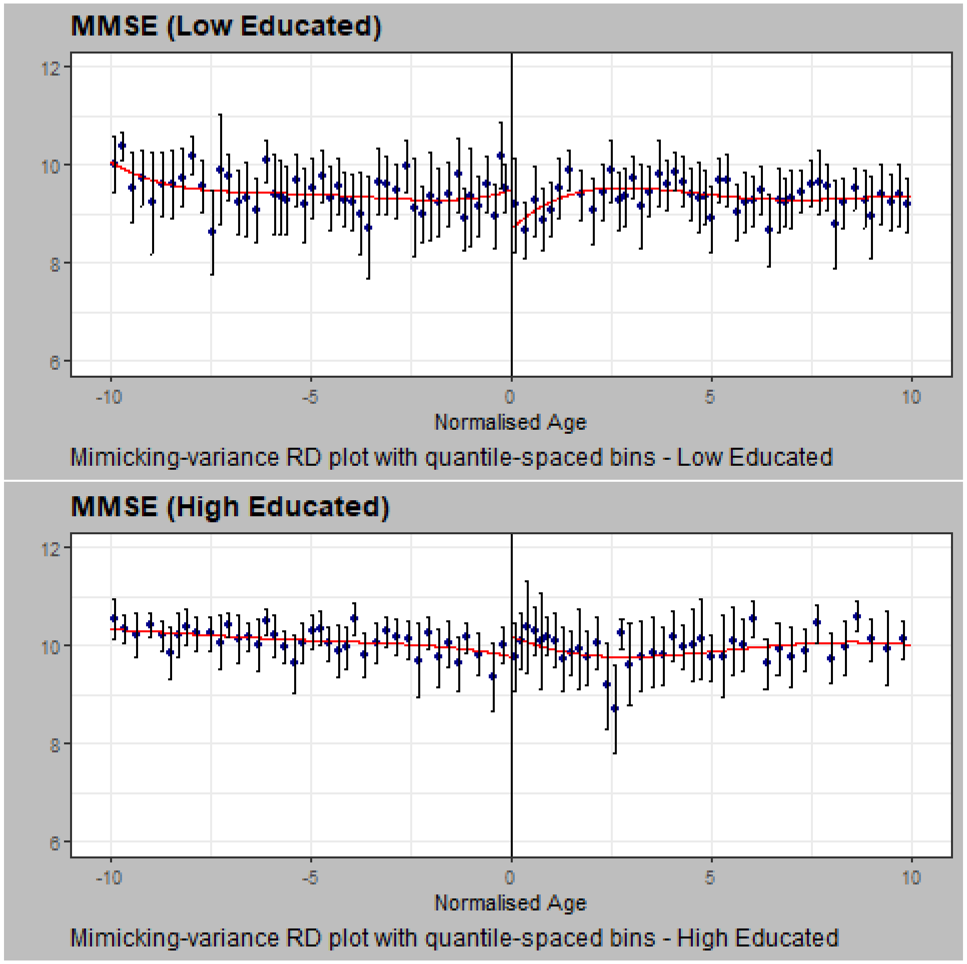

Fig. S34 ADL RD Plot by Education

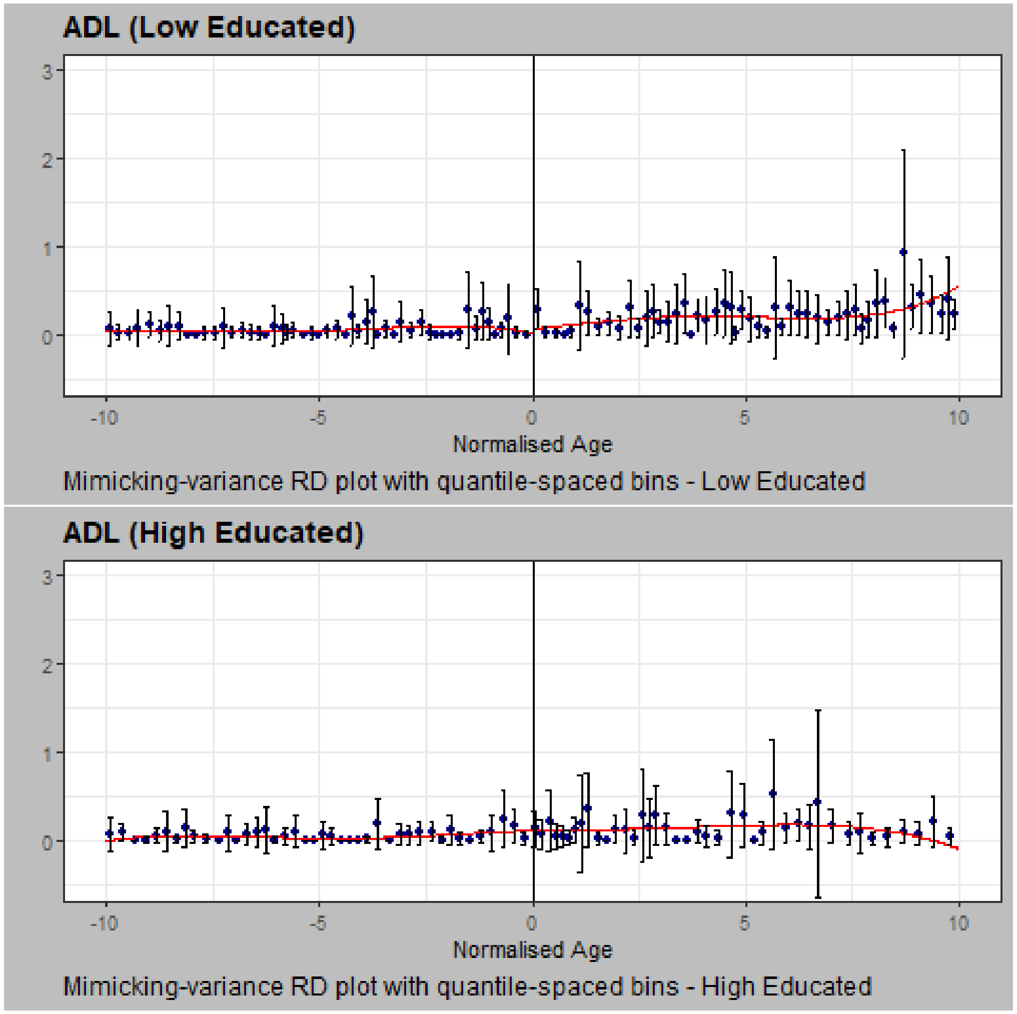

Fig. S35 IADL RD Plot by Education

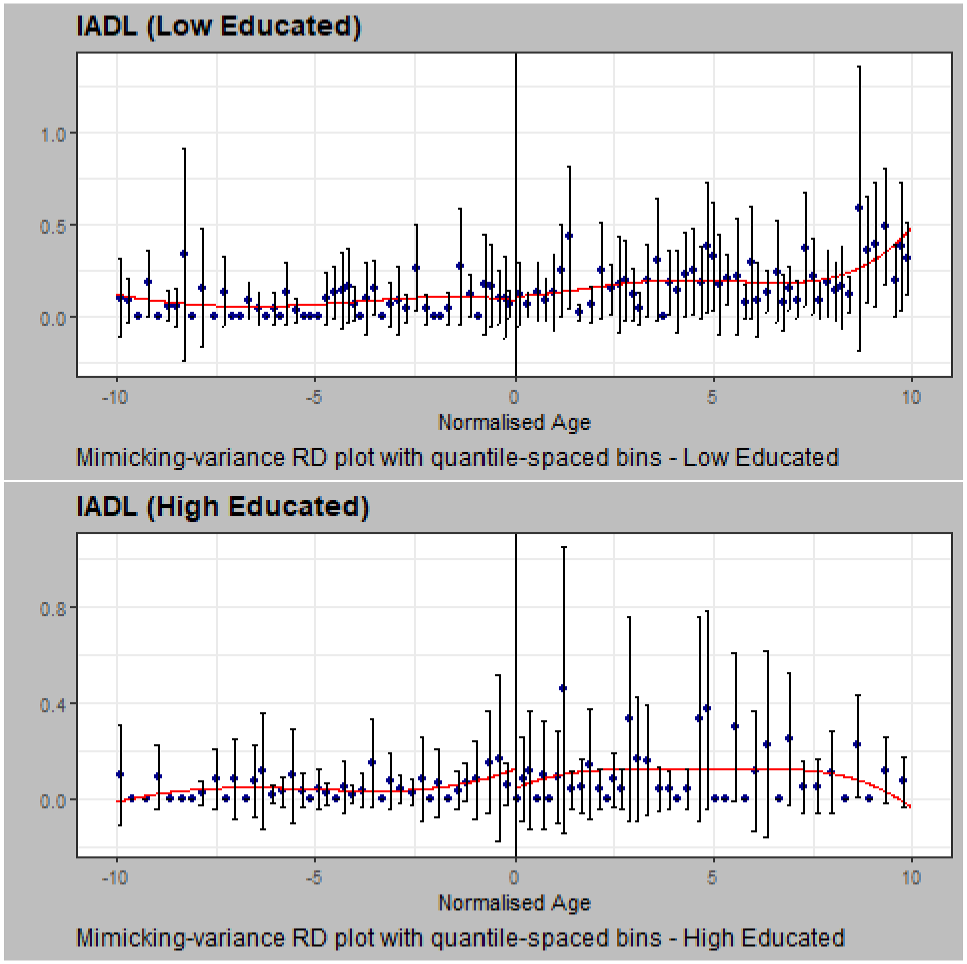

Fig. S36 Total Recall Score RD Plot by Education

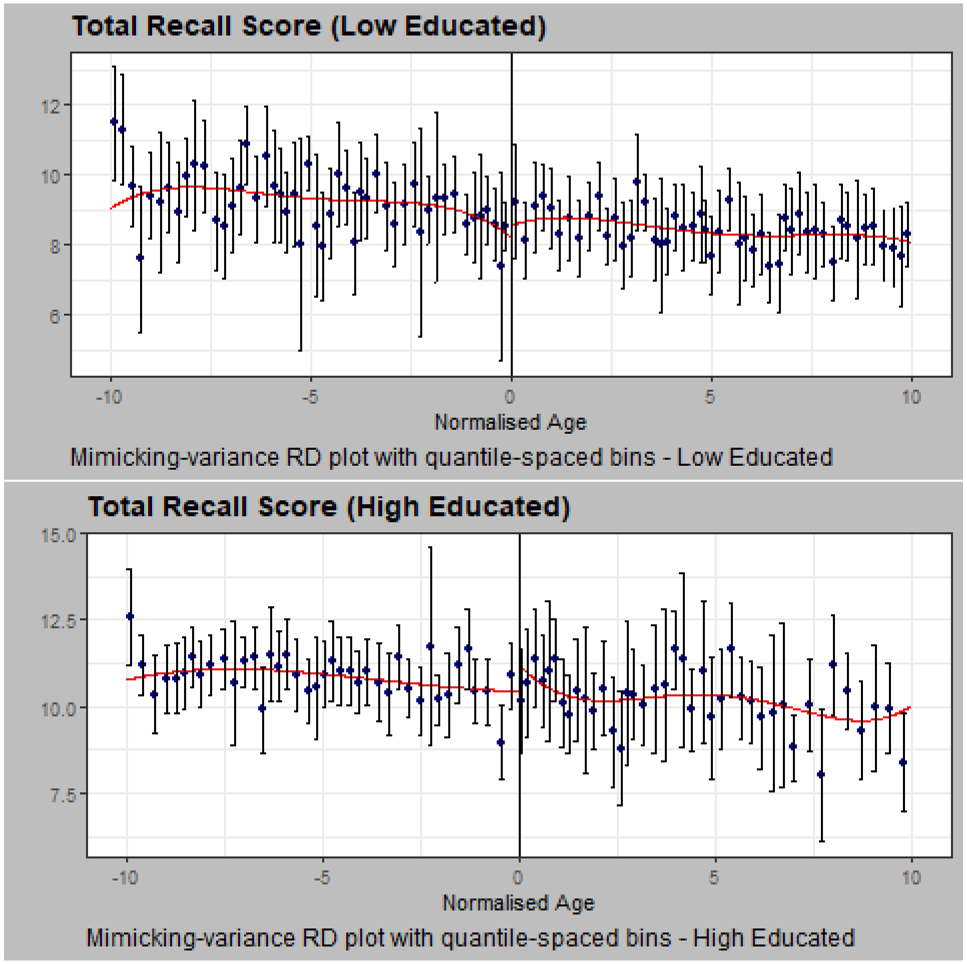

Fig. S37 Nonparametric Regression discontinuity robustness of bandwidth choice for self-reported health by education

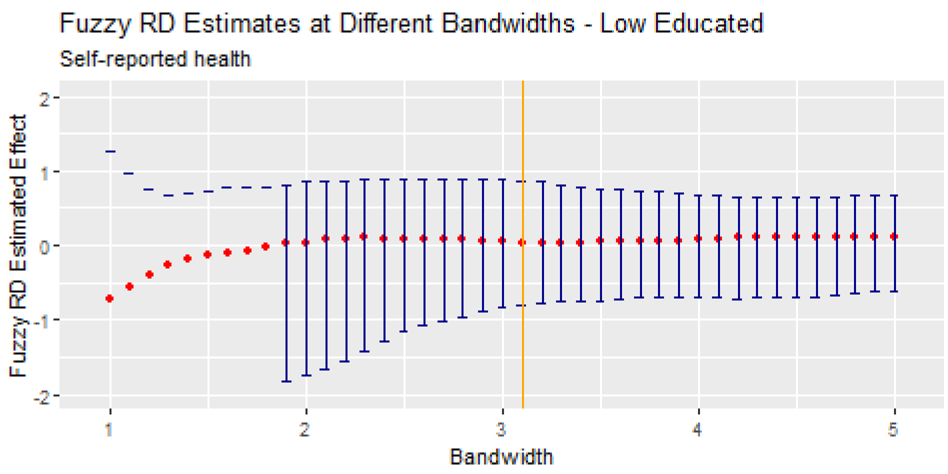

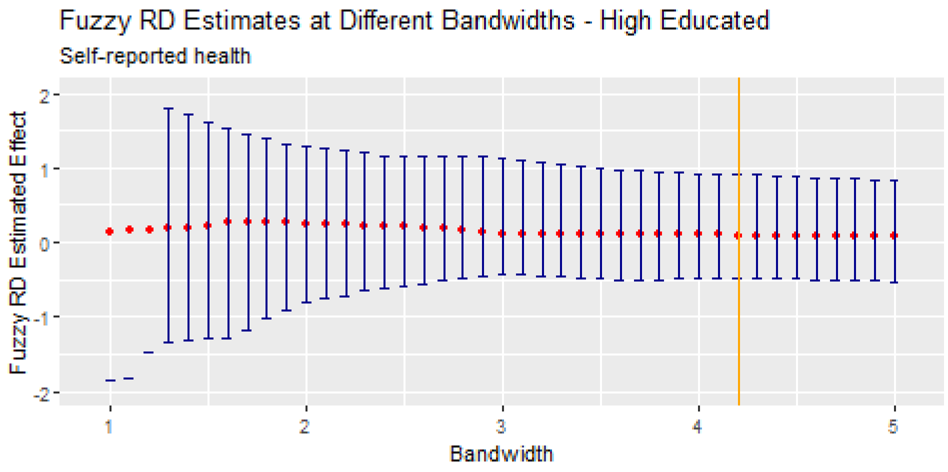

Fig. S38 Nonparametric Regression discontinuity robustness of bandwidth choice for life satisfaction by education

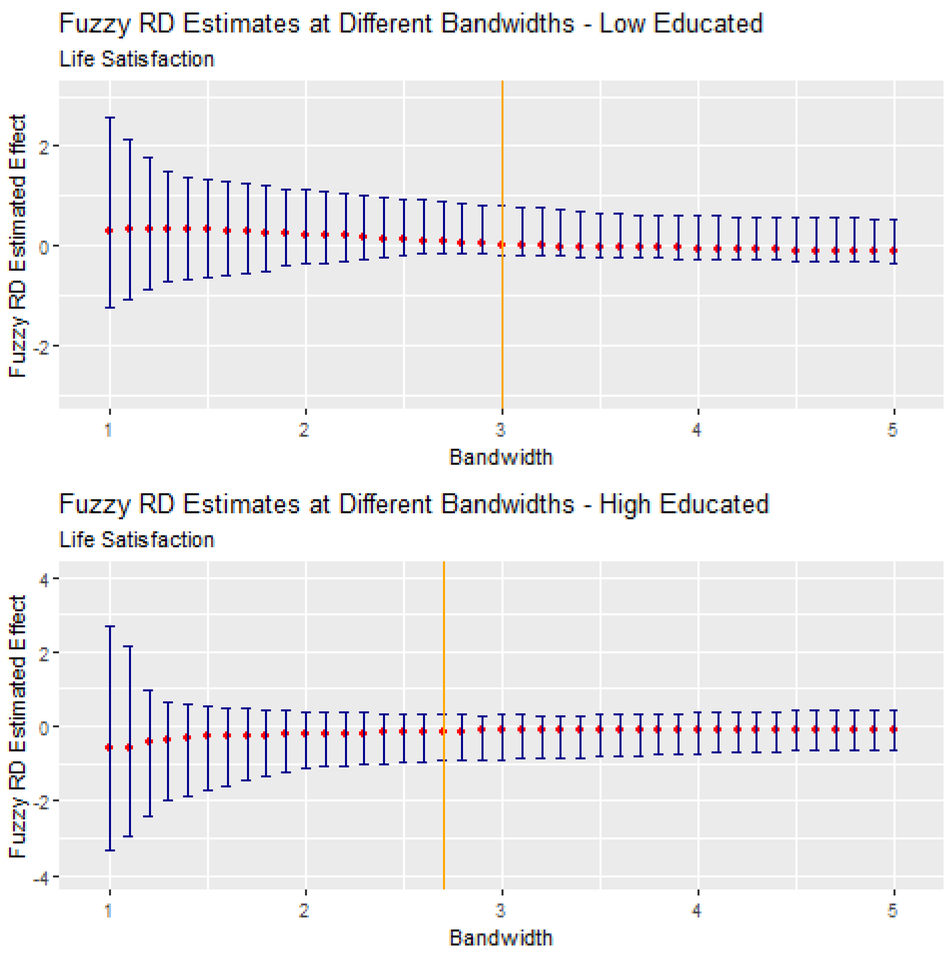

Fig. S39 Nonparametric Regression discontinuity robustness of bandwidth choice for CESD scale by education

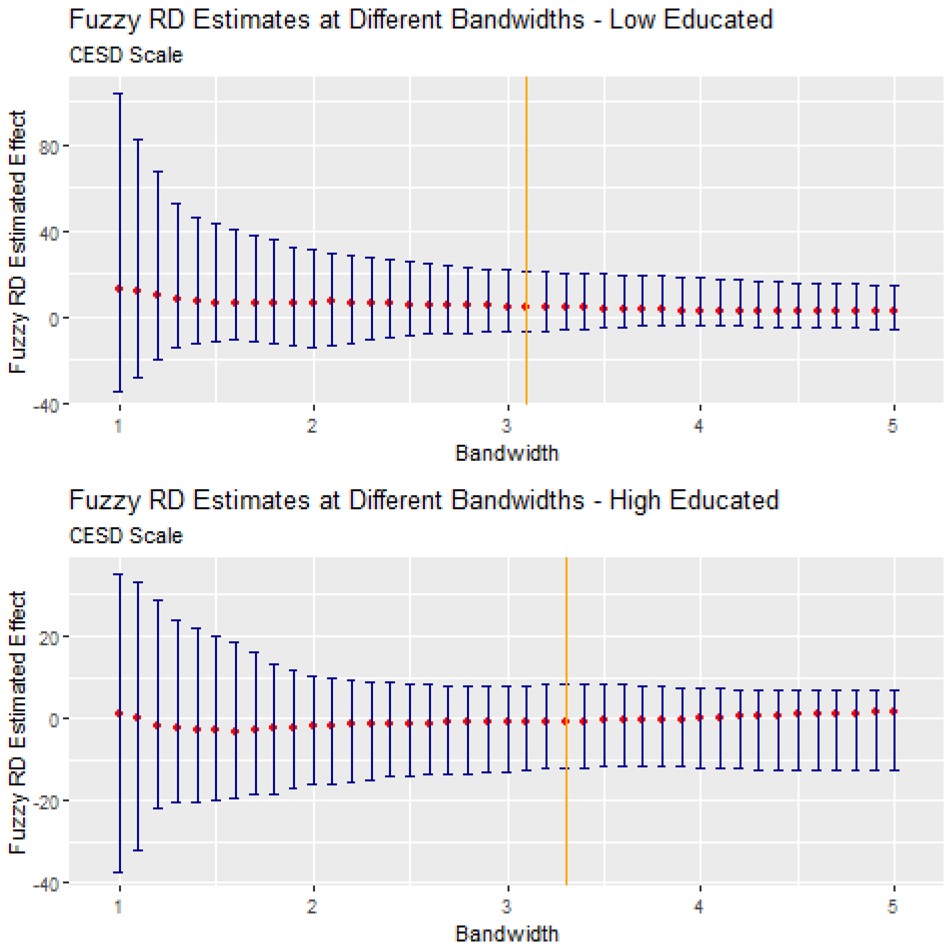

Fig. S40 Nonparametric Regression discontinuity robustness of bandwidth choice for MMSE by education

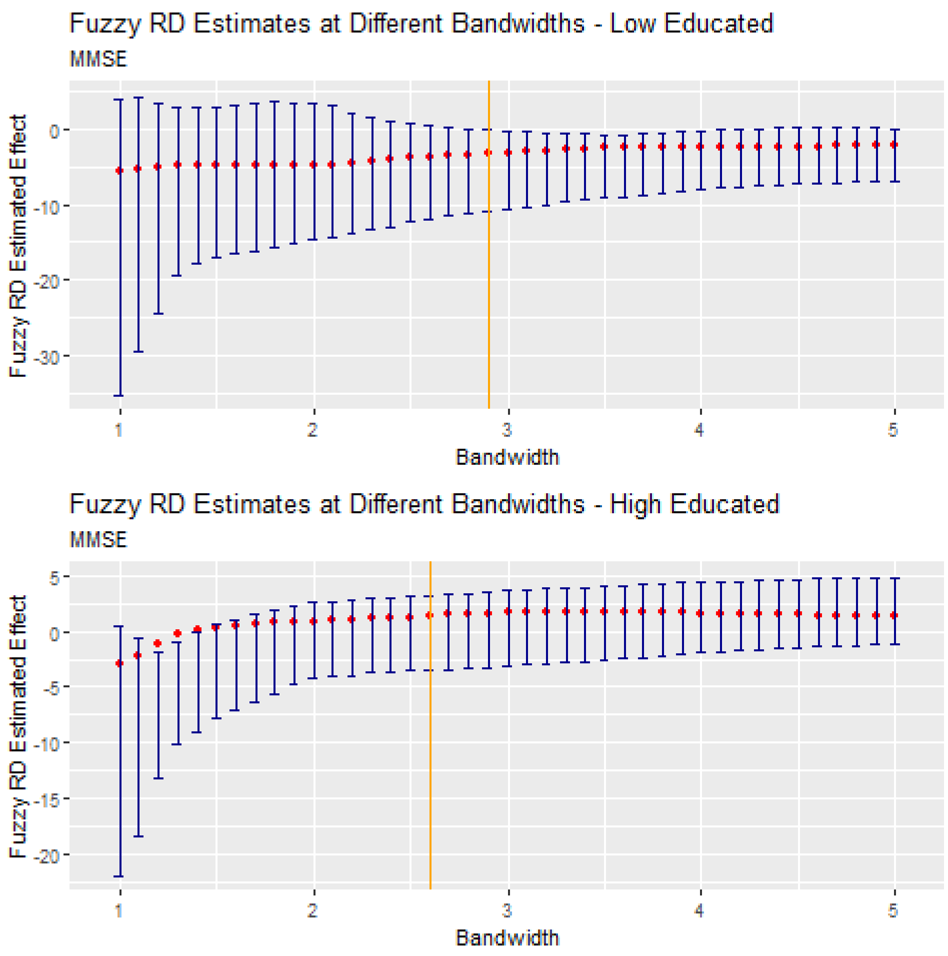

Fig. S41 Nonparametric Regression discontinuity robustness of bandwidth choice for ADL by education

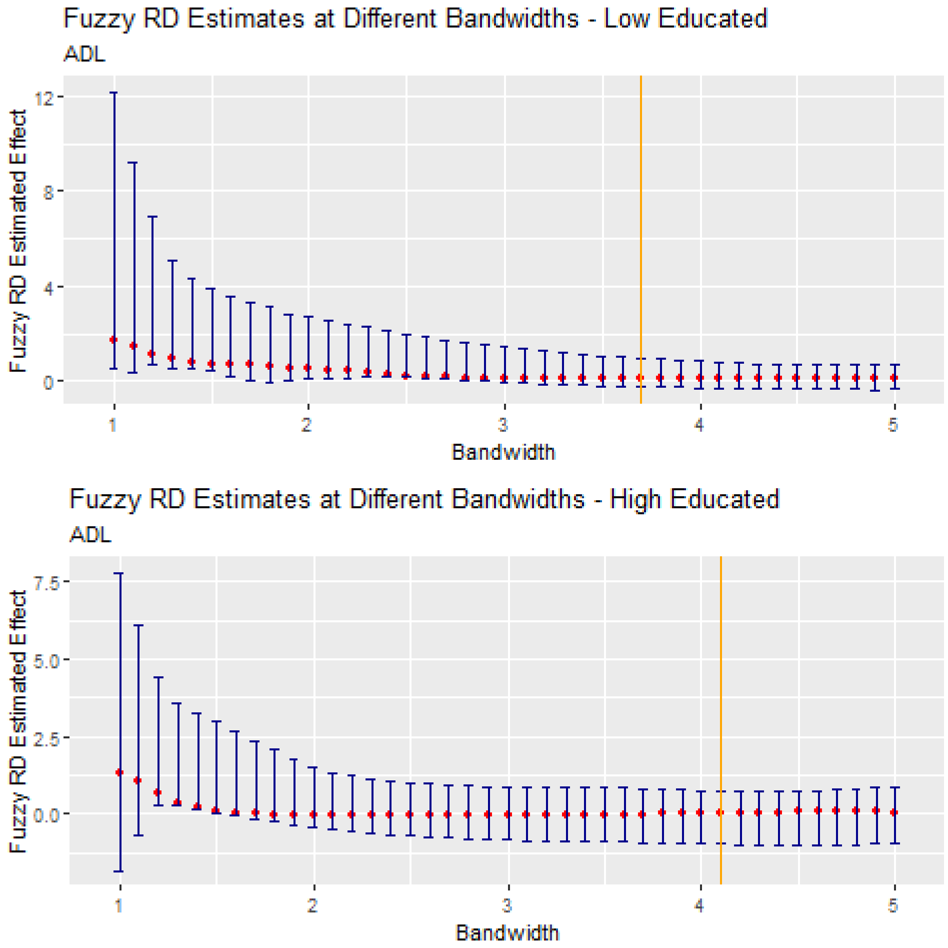

Fig. S42 Nonparametric Regression discontinuity robustness of bandwidth choice for IADL by education

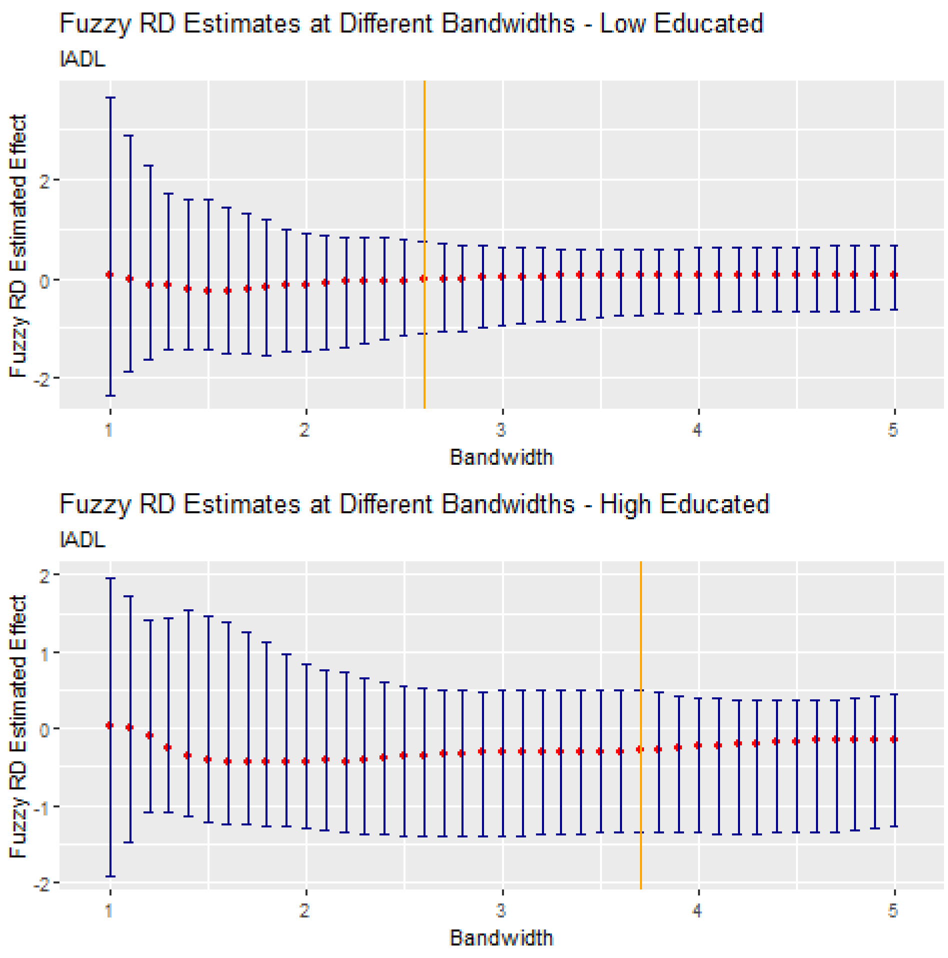

Fig. S43 Nonparametric Regression discontinuity robustness of bandwidth choice for Total recall score by education

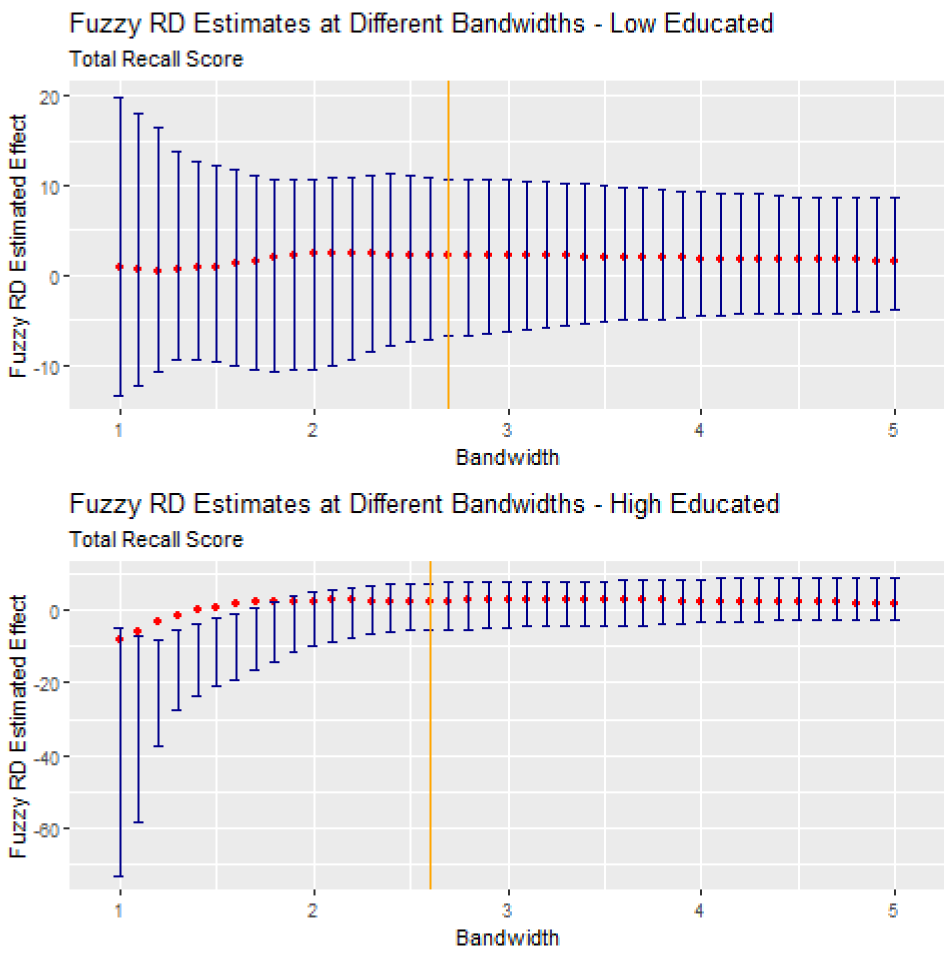

Fig. S44 The predetermined covariates by normalized age

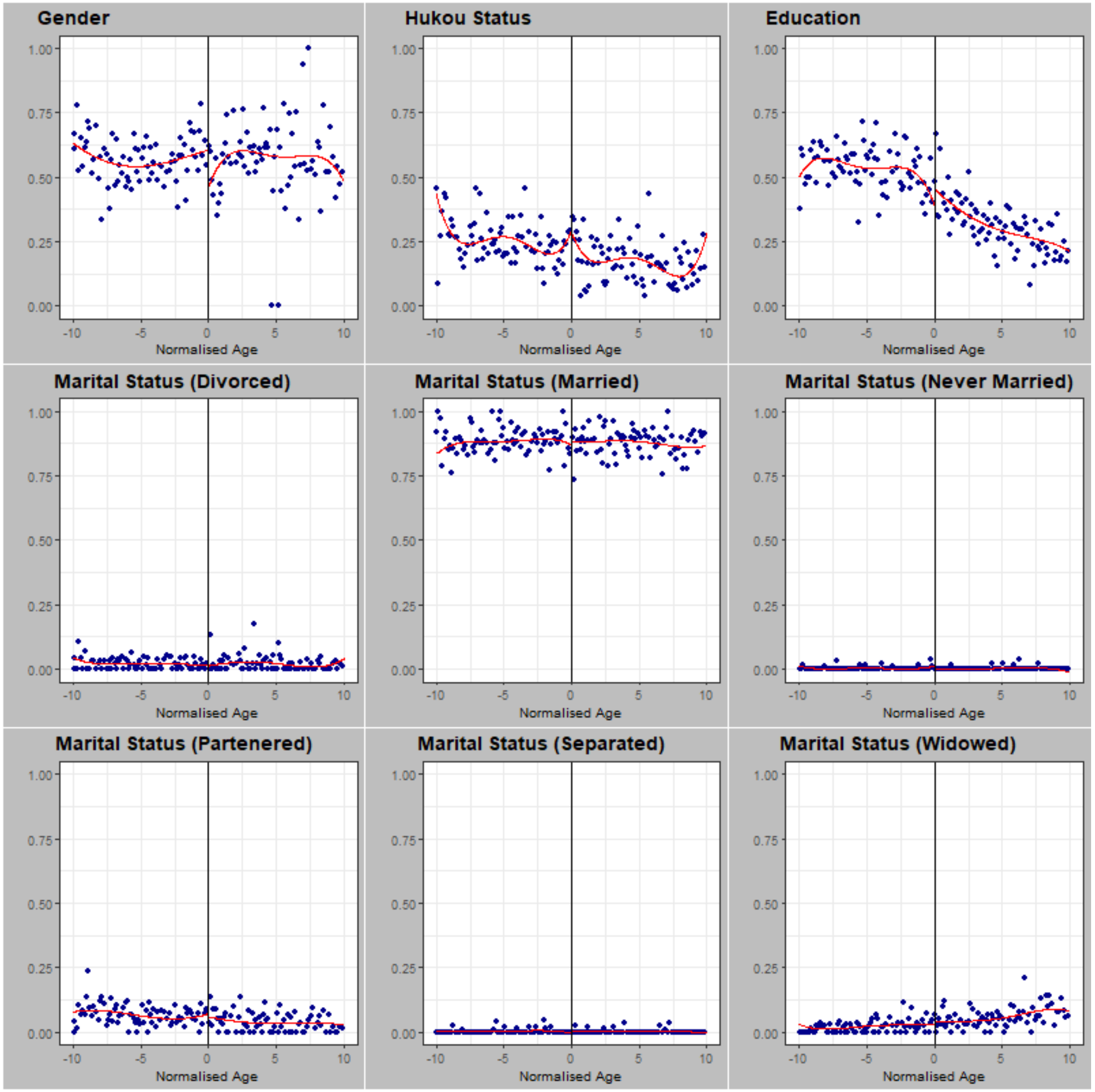

Note: We used mimicking variance quantile-spaced method to estimate the sample means.

Fig. S45 Running variable density test

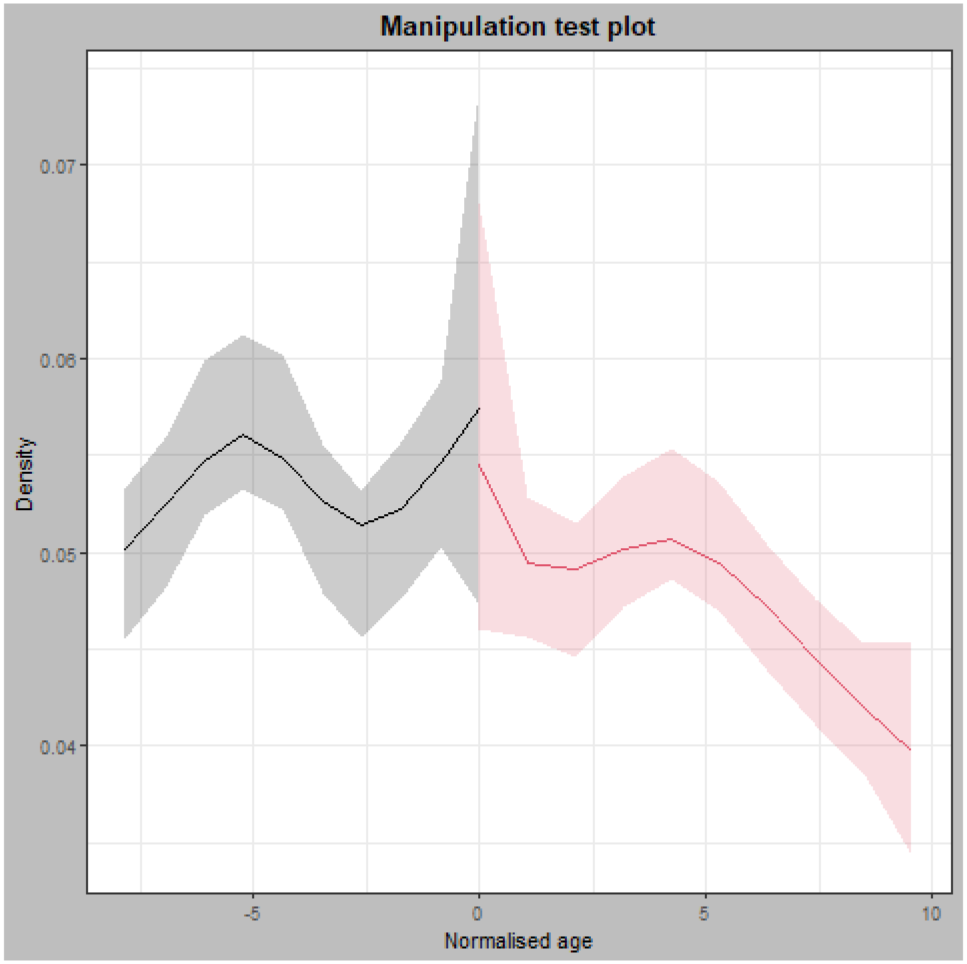

Note: (a) The manipulation test was constructed using a 2nd-order local-polynomial density estimator, an MSE-optimal bandwidth choice coupled with robust bias-correction, and the corresponding jackknife SE estimator. (b) The solid line represents point estimate, while the shaded area represents 95% CI.
