# Supplementary material for: The effect of retirement on physical and mental health in China: a nonparametric fuzzy regression discontinuity study
Source: BMC Public Health. 2024 Apr 27;24:1184. doi: 10.1186/s12889-024-18649-w (PMC11520119; doi:10.1186/s12889-024-18649-w)
Supplement: Supplementary file 2 [file 12889_2024_18649_MOESM1_ESM.docx]

Contents

Table S1. Flowchart of sample selection	5

Table S2. Cutoff age of female employees	6

Table S3. Fuzzy RD estimates at different bandwidths – Self-reported health – All samples at age 65	7

Table S4. Fuzzy RD estimates at different bandwidths – Life satisfaction – All samples at age 65	8

Table S5. Fuzzy RD estimates at different bandwidths – CESD Scale – All samples at age 65	9

Table S6. Fuzzy RD estimates at different bandwidths – the CHARLS cognitive battery – All samples at age 65	10

Table S7. Fuzzy RD estimates at different bandwidths – ADL – All samples at age 65	11

Table S8. Fuzzy RD estimates at different bandwidths – IADL Scale – All samples at age 65	12

Table S9. Fuzzy RD estimates at different bandwidths – Total Recall Score – All samples at age 65	13

Table S10. Fuzzy RD estimates at different bandwidths – Self-reported health – By gender at age 65	14

Table S11. Fuzzy RD estimates at different bandwidths – Life satisfaction – By gender at age 65	15

Table S12. Fuzzy RD estimates at different bandwidths – CESD Scale – By gender at age 65	16

Table S13. Fuzzy RD estimates at different bandwidths – the CHARLS cognitive battery – By gender at age 65	17

Table S14. Fuzzy RD estimates at different bandwidths – ADL – By gender at age 65	18

Table S15. Fuzzy RD estimates at different bandwidths – IADL Scale – By gender at age 65	19

Table S16. Fuzzy RD estimates at different bandwidths – Total recall score – By gender at age 65	20

Table S17. Fuzzy RD estimates at different bandwidths – Self-reported health – By education at age 65	21

Table S18. Fuzzy RD estimates at different bandwidths – Life satisfaction – By education at age 65	22

Table S19. Fuzzy RD estimates at different bandwidths – CESD Scale – By education at age 65	23

Table S20. Fuzzy RD estimates at different bandwidths – the CHARLS cognitive battery – By education at age 65	24

Table S21. Fuzzy RD estimates at different bandwidths – ADL – By education at age 65	25

Table S22. Fuzzy RD estimates at different bandwidths – IADL Scale – By education at age 65	26

Table S23. Fuzzy RD estimates at different bandwidths – Total recall score – By education at age 65	27

Table S24. Nonparametric fuzzy regression discontinuity (RD) estimates—Gender heterogeneity at age 65	28

Table S25. Nonparametric fuzzy regression discontinuity (RD) estimates—Education heterogeneity at age 65	29

Table S26. Formal falsification test for the validity of the Continuity-Based Analysis for predetermined Covariates	30

Table S27. Manipulation test of running variable	31

Table S28. Placebo cutoff	32

Table S29. Fuzzy RD sensitivity test to observations near cutoff: The Donut-Hole Approach	33

Figure S1. Cutoff age for female employees	34

Figure S2. Self-reported Health RD Plot	35

Figure S3. Life Satisfaction RD Plot	36

Figure S4. CESD Scale RD Plot	37

Figure S6. ADL RD Plot	39

Figure S7. IADL RD Plot	40

Figure S8. Total Recall Score RD Plot	41

Figure S10. Nonparametric Regression discontinuity robustness of bandwidth choice for life satisfaction	43

Figure S11. Nonparametric Regression discontinuity robustness of bandwidth choice for CESD Scale	44

Figure S12. Nonparametric Regression discontinuity robustness of bandwidth choice for the CHARLS cognitive battery	45

Figure S13. Nonparametric Regression discontinuity robustness of bandwidth choice for ADL	46

Figure S14. Nonparametric Regression discontinuity robustness of bandwidth choice for IADL	47

Figure S15. Nonparametric Regression discontinuity robustness of bandwidth choice for total recall score	48

Figure S16. Self-reported Health RD Plot by Gender	49

Figure S17. Life Satisfaction RD Plot by Gender	50

Figure S18. CESD Scale RD Plot by Gender	51

Figure S19. The CHARLS Cognitive Battery RD Plot by Gender	52

Figure S20. ADL RD Plot by Gender	53

Fig. S21 IADL RD Plot by Gender	54

Figure S22. Total Recall Score RD Plot by Gender	55

Figure S23. Nonparametric Regression discontinuity robustness of bandwidth choice for self-reported health by gender	56

Figure S24. Nonparametric Regression discontinuity robustness of bandwidth choice for life satisfaction by gender	57

Fig. S25 Nonparametric Regression discontinuity robustness of bandwidth choice for CESD scale by gender	58

Fig. S26 Nonparametric Regression discontinuity robustness of bandwidth choice for the CHARLS cognitive battery by gender	59

Figure S27. Nonparametric Regression discontinuity robustness of bandwidth choice for ADL by gender	60

Figure S28. Nonparametric Regression discontinuity robustness of bandwidth choice for IADL by gender	61

Figure S29. Nonparametric Regression discontinuity robustness of bandwidth choice for Total recall score by gender	62

Figure S30. Self-reported Health RD Plot by Education	63

Figure S31. Life Satisfaction RD Plot by Education	64

Figure S32. CESD Scale RD Plot by Education	65

Figure S33. the CHARLS Cognitive Battery RD Plot by Education	66

Figure S34. ADL RD Plot by Education	67

Figure S35. IADL RD Plot by Education	68

Figure S36. Total Recall Score RD Plot by Education	69

Figure S37. Nonparametric Regression discontinuity robustness of bandwidth choice for self-reported health by education	70

Figure S38. Nonparametric Regression discontinuity robustness of bandwidth choice for life satisfaction by education	71

Figure S39. Nonparametric Regression discontinuity robustness of bandwidth choice for CESD scale by education	72

Figure S40. Nonparametric Regression discontinuity robustness of bandwidth choice for the CHARLS cognitive battery by education	73

Figure S41. Nonparametric Regression discontinuity robustness of bandwidth choice for ADL by education	74

Figure S42. Nonparametric Regression discontinuity robustness of bandwidth choice for IADL by education	75

Figure S43. Nonparametric Regression discontinuity robustness of bandwidth choice for Total recall score by education	76

Figure S44. The predetermined covariates by normalized age	77

Figure S45. Running variable density test	78

Table S1. Flowchart of sample selection

|  | Person-waves | Individuals |
| --- | --- | --- |
| Total sample size in four waves | 102,344 | 25,586 |
| Keep Participants working in government/ have basic pension plans with firms | 10,855 | 5,825 |
| Keep males aged 50 - 70, and females aged 45 – 65 | 6,965 | 4,039 |
| Drop participants never worked or employed | 6,888 | 4,010 |
| Drop participants retired due to health reasons | 6,678 | 3,913 |
| Drop missing values in the variable “retired or not” | 6,503 | 3,837 |
| Drop missing values in marital status | 6,502 | 3,837 |
| Drop missing values in the Hukou status | 6,122 | 3,564 |
| Drop missing values in the outcome variable |  |  |
| Self-reported health | 5,803 | 3,405 |
| Life satisfaction | 5,615 | 3,309 |
| CESD scale | 5,643 | 3,318 |
| ADL | 6,045 | 3,532 |
| IADL | 5,592 | 3,380 |
| Total recall score | 5,460 | 3,222 |
| the CHARLS cognitive battery | 5,274 | 3,316 |
| Physical activity | 3,673 | 2,715 |
| Drinking | 6,115 | 3,562 |
| Smoking | 5,698 | 3,422 |

Table S2. Cutoff age of female employees

|  | Cutoff at 55 | Cutoff at 50 |
| --- | --- | --- |
| Optimal bandwidth | 2.69 | 1.01 |
| Conventional | 0.1075 | 0.2612 |
| P-value | 0.1598 | 0.0200** |
| Conventional 95% CI | [-0.0424, 0.2573] | [0.0411, 0.4813] |
| Robust | 0.1089 | 0.2759 |
| P-value | 0.1887 | 0.0235** |
| Robust 95% CI | [-0.0535, 0.2713] | [0.0372, 0.5145] |
| Obs L│R | 1371│1252 | 670│1953 |
| Effective obs | 351│344 | 155│159 |

Note: (i) All estimates are computed using a triangular Kernel and local linear regression. (ii) Bandwidth h, for the RD estimator, and bandwidth b for the bias-corrected estimator is data-driven coverage-error optimal for inference. (iii) Effective number of observations depends on the size of the data-driven selected bandwidth.

** indicates a significance level at 5%.

Table S3. Fuzzy RD estimates at different bandwidths – Self-reported health – All samples at age 65

| Bandwidths | Conventional Estimate | 95% CI (Conventional) | 95% CI (Robust) | Obs L│R |
| --- | --- | --- | --- | --- |
| 3.3 | 0.0808 | [-0.2842, 0.4459] | [-0.3574, 0.7292] | 3001│2802 |
| 1 | -0.2455 | [-1.3996, 0.9085] | [-2.2229, 1.3516] | 3001│2802 |
| 1.5 | 0.0578 | [-0.5857, 0.7012] | [-1.2637, 0.7118] | 3001│2802 |
| 2 | 0.1739 | [-0.3599, 0.7077] | [-0.8590, 0.7319] | 3001│2802 |
| 2.5 | 0.1676 | [-0.2778, 0.6130] | [-0.5611, 0.7654] | 3001│2802 |
| 3 | 0.0936 | [-0.2965, 0.4837] | [-0.3717, 0.7922] | 3001│2802 |
| 3.5 | 0.0863 | [-0.2684, 0.4410] | [-0.3714, 0.6834] | 3001│2802 |
| 4 | 0.0916 | [-0.2400, 0.4233] | [-0.3719, 0.6085] | 3001│2802 |
| 4.5 | 0.1076 | [-0.2155, 0.4307] | [-0.3878, 0.5639] | 3001│2802 |
| 5 | 0.1080 | [-0.2064, 0.4225] | [-0.3665, 0.5558] | 3001│2802 |

Note: (i) All estimates are computed using a triangular Kernel and local linear regression. (ii) The bandwidth in the first line is the MSE optimal bandwidth. 

Table S4. Fuzzy RD estimates at different bandwidths – Life satisfaction – All samples at age 65

| Bandwidths | Estimate | 95% CI (Conventional) | 95% CI (Robust) | Obs L│R |
| --- | --- | --- | --- | --- |
| 3.8 | -0.0567 | [-0.303, 0.1897] | [-0.3504, 0.3503] | 2868│2747 |
| 1 | -0.1129 | [-0.8646, 0.6388] | [-1.5143, 1.0699] | 2868│2747 |
| 1.5 | 0.0368 | [-0.3941, 0.4677] | [-0.7826, 0.527] | 2868│2747 |
| 2 | -0.0063 | [-0.3507, 0.3381] | [-0.4838, 0.5161] | 2868│2747 |
| 2.5 | -0.0227 | [-0.3209, 0.2755] | [-0.4026, 0.4511] | 2868│2747 |
| 3 | -0.0422 | [-0.317, 0.2327] | [-0.3788, 0.402] | 2868│2747 |
| 3.5 | -0.0462 | [-0.3002, 0.2079] | [-0.3709, 0.3513] | 2868│2747 |
| 4 | -0.0709 | [-0.3119, 0.1701] | [-0.3289, 0.3551] | 2868│2747 |
| 4.5 | -0.1012 | [-0.339, 0.1366] | [-0.3116, 0.3634] | 2868│2747 |
| 5 | -0.1231 | [-0.3571, 0.1109] | [-0.3162, 0.349] | 2868│2747 |

Note: (i) All estimates are computed using a triangular Kernel and local linear regression. (ii) The bandwidth in the first line is the MSE optimal bandwidth. 

Table S5. Fuzzy RD estimates at different bandwidths – CESD Scale – All samples at age 65

| Bandwidths | Estimate | 95% CI (Conventional) | 95% CI (Robust) | Obs L│R |
| --- | --- | --- | --- | --- |
| 3.6 | 1.4883 | [-3.7619, 6.7386] | [-5.3991, 10.2902] | 2892│2751 |
| 1 | 7.2653 | [-14.5082, 29.0389] | [-26.6100, 50.2505] | 2892│2751 |
| 1.5 | 1.7965 | [-7.9961, 11.589] | [-7.7480, 23.7890] | 2892│2751 |
| 2 | 1.7833 | [-5.9205, 9.4871] | [-8.9118, 14.5444] | 2892│2751 |
| 2.5 | 1.8201 | [-4.6489, 8.2890] | [-7.4046, 12.0228] | 2892│2751 |
| 3 | 1.8801 | [-3.9705, 7.7307] | [-6.8255, 10.7155] | 2892│2751 |
| 3.5 | 1.5895 | [-3.7496, 6.9286] | [-5.6404, 10.3139] | 2892│2751 |
| 4 | 1.4474 | [-3.5288, 6.4235] | [-5.2520, 9.5722] | 2892│2751 |
| 4.5 | 1.9047 | [-2.9530, 6.7624] | [-5.9824, 8.4645] | 2892│2751 |
| 5 | 2.2452 | [-2.4975, 6.9879] | [-6.0964, 7.9783] | 2892│2751 |

Note: (i) All estimates are computed using a triangular Kernel and local linear regression. (ii) The bandwidth in the first line is the MSE optimal bandwidth. 

Table S6. Fuzzy RD estimates at different bandwidths – the CHARLS cognitive battery – All samples at age 65

| Bandwidths | Estimate | 95% CI (Conventional) | 95% CI (Robust) | Obs L│R |
| --- | --- | --- | --- | --- |
| 3.6 | -0.4173 | [-2.0094, 1.1748] | [-3.9535, 0.6785] | 2733│2541 |
| 1 | -4.1120* | [-10.6881, 2.4640] | [-21.5954, -0.6002] | 2733│2541 |
| 1.5 | -2.0347* | [-5.0309, 0.9615] | [-9.2730, -0.6252] | 2733│2541 |
| 2 | -1.5438 | [-3.9104, 0.8229] | [-6.4519, 0.1893] | 2733│2541 |
| 2.5 | -1.0257 | [-3.0065, 0.9551] | [-5.3085, 0.2965] | 2733│2541 |
| 3 | -0.6779 | [-2.4547, 1.0989] | [-4.6434, 0.4580] | 2733│2541 |
| 3.5 | -0.4491 | [-2.0639, 1.1657] | [-4.0503, 0.6380] | 2733│2541 |
| 4 | -0.4660 | [-2.0001, 1.0682] | [-3.3839, 1.0889] | 2733│2541 |
| 4.5 | -0.5823 | [-2.0929, 0.9283] | [-2.9314, 1.4754] | 2733│2541 |
| 5 | -0.6129 | [-2.0813, 0.8556] | [-2.7897, 1.4987] | 2733│2541 |

Note: (i) All estimates are computed using a triangular Kernel and local linear regression. (ii) The bandwidth in the first line is the MSE optimal bandwidth. 

(iii) * indicates a significance level at 10%.

Table S7. Fuzzy RD estimates at different bandwidths – ADL – All samples at age 65

| Bandwidths | Estimate | 95% CI (Conventional) | 95% CI (Robust) | Obs L│R |
| --- | --- | --- | --- | --- |
| 4 | 0.0394 | [-0.3672, 0.4461] | [-0.3964, 0.5935] | 3122│2923 |
| 1 | 1.4357* | [-0.7219, 3.5934] | [0.7599, 7.6802] | 3122│2923 |
| 1.5 | 0.3946*** | [-0.2491, 1.0382] | [0.7361, 2.7326] | 3122│2923 |
| 2 | 0.1951* | [-0.3162, 0.7064] | [0.2159, 1.6410] | 3122│2923 |
| 2.5 | 0.0667 | [-0.3806, 0.5140] | [-0.0357, 1.1917] | 3122│2923 |
| 3 | 0.0281 | [-0.4067, 0.4629] | [-0.2127, 0.9235] | 3122│2923 |
| 3.5 | 0.0231 | [-0.3960, 0.4423] | [-0.3191, 0.7270] | 3122│2923 |
| 4 | 0.0394 | [-0.3672, 0.4461] | [-0.3964, 0.5935] | 3122│2923 |
| 4.5 | 0.0628 | [-0.3410, 0.4666] | [-0.4476, 0.5340] | 3122│2923 |
| 5 | 0.0513 | [-0.3494, 0.4520] | [-0.4220, 0.5603] | 3122│2923 |

Note: (i) All estimates are computed using a triangular Kernel and local linear regression. (ii) The bandwidth in the first line is the MSE optimal bandwidth.  

(iii) *, **, *** indicate significance level at 10%, 5%, 1% respectively. 

Table S8. Fuzzy RD estimates at different bandwidths – IADL Scale – All samples at age 65

| Bandwidths | Estimate | 95% CI (Conventional) | 95% CI (Robust) | Obs L│R |
| --- | --- | --- | --- | --- |
| 2.8 | -0.1860 | [-0.6735, 0.3015] | [-0.9916, 0.2677] | 2720│2872 |
| 1 | 0.0395 | [-0.8706, 0.9550] | [-1.4380, 1.7860] | 2720│2872 |
| 1.5 | -0.3436 | [-1.0218, 0.3347] | [-0.8885, 1.0386] | 2720│2872 |
| 2 | -0.3054 | [-0.9081, 0.2973] | [-1.0164, 0.5247] | 2720│2872 |
| 2.5 | -0.2419 | [-0.7621, 0.2782] | [-1.0056, 0.3405] | 2720│2872 |
| 3 | -0.1630 | [-0.6356, 0.3096] | [-0.9504, 0.2587] | 2720│2872 |
| 3.5 | -0.1228 | [-0.5615, 0.3160] | [-0.8594, 0.2413] | 2720│2872 |
| 4 | -0.0837 | [-0.5008, 0.3335] | [-0.8072, 0.2322] | 2720│2872 |
| 4.5 | -0.0416 | [-0.4519, 0.3688] | [-0.7873, 0.2398] | 2720│2872 |
| 5 | -0.0413 | [-0.4457, 0.3630] | [-0.7034, 0.3215] | 2720│2872 |

Note: (i) All estimates are computed using a triangular Kernel and local linear regression. (ii) The bandwidth in the first line is the MSE optimal bandwidth.  

Table S9. Fuzzy RD estimates at different bandwidths – Total Recall Score – All samples at age 65

| Bandwidths | Estimate | 95% CI (Conventional) | 95% CI (Robust) | Obs L│R |
| --- | --- | --- | --- | --- |
| 3.9 | 2.3377 | [-1.1357, 5.8111] | [-2.0721, 8.1252] | 2803│2657 |
| 1 | -2.705 | [-12.9924, 7.5825] | [-31.4250, 2.1186] | 2803│2657 |
| 1.5 | 1.2297 | [-4.4555, 6.9148] | [-12.9818, 3.8696] | 2803│2657 |
| 2 | 3.0086 | [-1.9638, 7.9810] | [-8.0789, 6.2567] | 2803│2657 |
| 2.5 | 2.7617 | [-1.5548, 7.0781] | [-4.4101, 8.0971] | 2803│2657 |
| 3 | 2.7142 | [-1.2953, 6.7237] | [-3.3379, 8.3971] | 2803│2657 |
| 3.5 | 2.5899 | [-1.0952, 6.2751] | [-2.6714, 8.1526] | 2803│2657 |
| 4 | 2.2463 | [-1.1669, 5.6595] | [-1.8819, 8.1363] | 2803│2657 |
| 4.5 | 1.9584 | [-1.3152, 5.2320] | [-1.6264, 7.9840] | 2803│2657 |
| 5 | 1.6654 | [-1.4773, 4.8082] | [-1.3710, 7.8473] | 2803│2657 |

Note: (i) All estimates are computed using a triangular Kernel and local linear regression. (ii) The bandwidth in the first line is the MSE optimal bandwidth.  

Table S10. Fuzzy RD estimates at different bandwidths – Self-reported health – By gender at age 65

| Male |  |  |  |  | Female |  |  |  |  |
| --- | --- | --- | --- | --- | --- | --- | --- | --- | --- |
| Bandwidths | Estimate | 95% CI (Conventional) | 95% CI (Robust) | Obs L│R | Bandwidths | Estimate | 95% CI (Conventional) | 95% CI (Robust) | Obs L│R |
| 3.2 | 0.3479 | [-0.2031, 0.8988] | [-0.1049, 1.5100] | 1709│1610 | 3.6 | -0.3755 | [-1.4092, 0.6583] | [-2.5867, 0.436] | 1292│1192 |
| 1 | 0.3966 | [-0.4262, 1.2194] | [-0.4153, 2.3239] | 1709│1610 | 1 | 21.2581 | [-660.4485, 702.9648] | [-1381.8309, 881.7232] | 1292│1192 |
| 1.5 | 0.4693 | [-0.2518, 1.1904] | [-0.7451, 1.4637] | 1709│1610 | 1.5 | -2.638 | [-14.9302, 9.6541] | [-31.4602, 6.5034] | 1292│1192 |
| 2 | 0.5717 | [-0.1935, 1.3368] | [-0.7708, 1.4644] | 1709│1610 | 2 | -1.0597 | [-3.8539, 1.7344] | [-7.8592, 0.4744] | 1292│1192 |
| 2.5 | 0.5154 | [-0.1675, 1.1983] | [-0.4175, 1.5737] | 1709│1610 | 2.5 | -0.6367 | [-2.1538, 0.8803] | [-4.1944, 0.3138] | 1292│1192 |
| 3 | 0.3923 | [-0.2031, 0.9877] | [-0.1733, 1.5712] | 1709│1610 | 3 | -0.5361 | [-1.7411, 0.6689] | [-3.0544, 0.5056] | 1292│1192 |
| 3.5 | 0.3142 | [-0.1913, 0.8197] | [-0.0963, 1.3831] | 1709│1610 | 3.5 | -0.4060 | [-1.4666, 0.6546] | [-2.6450, 0.4595] | 1292│1192 |
| 4 | 0.2653 | [-0.1874, 0.7180] | [-0.0948, 1.2274] | 1709│1610 | 4 | -0.2652 | [-1.2000, 0.6696] | [-2.3326, 0.3780] | 1292│1192 |
| 4.5 | 0.2527 | [-0.1770, 0.6825] | [-0.1563, 1.1003] | 1709│1610 | 4.5 | -0.1750 | [-1.0703, 0.7203] | [-2.1369, 0.4393] | 1292│1192 |
| 5 | 0.2400 | [-0.1718, 0.6518] | [-0.1852, 1.0216] | 1709│1610 | 5 | -0.1443 | [-1.0015, 0.7129] | [-1.8772, 0.5529] | 1292│1192 |

Note: (i) All estimates are computed using a triangular Kernel and local linear regression. (ii) The bandwidth in the first line is the MSE optimal bandwidth.  

Table S11. Fuzzy RD estimates at different bandwidths – Life satisfaction – By gender at age 65

| Male |  |  |  |  | Female |  |  |  |  |
| --- | --- | --- | --- | --- | --- | --- | --- | --- | --- |
| Bandwidths | Estimate | 95% CI (Conventional) | 95% CI (Robust) | Obs L│R | Bandwidths | Estimate | 95% CI (Conventional) | 95% CI (Robust) | Obs L│R |
| 2.9 | 0.2108 | [-0.1418, 0.5633] | [-0.2712, 0.6997] | 1626│1584 | 3.8 | -0.4077 | [-1.2067, 0.3912] | [-1.8136, 0.6824] | 1242│1163 |
| 1 | 0.1250 | [-0.3358, 0.5857] | [-0.5524, 1.5331] | 1626│1584 | 1 | -1.5693 | [-11.1657, 8.0271] | [-21.8942, 8.1543] | 1242│1163 |
| 1.5 | 0.2158 | [-0.1629, 0.5945] | [-0.6304, 0.7355] | 1626│1584 | 1.5 | -0.5417 | [-2.6100, 1.5266] | [-4.6881, 1.2601] | 1242│1163 |
| 2 | 0.2168 | [-0.1505, 0.5842] | [-0.4618, 0.7075] | 1626│1584 | 2 | -0.5129 | [-1.8940, 0.8683] | [-2.8103, 1.2062] | 1242│1163 |
| 2.5 | 0.2333 | [-0.1299, 0.5965] | [-0.3596, 0.7018] | 1626│1584 | 2.5 | -0.4942 | [-1.5696, 0.5812] | [-2.1741, 1.0101] | 1242│1163 |
| 3 | 0.2034 | [-0.1458, 0.5526] | [-0.2516, 0.6984] | 1626│1584 | 3 | -0.4621 | [-1.3732, 0.4491] | [-1.9497, 0.8189] | 1242│1163 |
| 3.5 | 0.1569 | [-0.1541, 0.4679] | [-0.1262, 0.6729] | 1626│1584 | 3.5 | -0.4180 | [-1.2508, 0.4148] | [-1.8852, 0.7010] | 1242│1163 |
| 4 | 0.1032 | [-0.1835, 0.3900] | [-0.0518, 0.6611] | 1626│1584 | 4 | -0.4111 | [-1.1887, 0.3666] | [-1.7398, 0.6862] | 1242│1163 |
| 4.5 | 0.2527 | [-0.1770, 0.6825] | [-0.1563, 1.1003] | 1709│1610 | 4.5 | -0.4609 | [-1.2620, 0.3401] | [-1.6387, 0.8433] | 1242│1163 |
| 5 | 0.2400 | [-0.1718, 0.6518] | [-0.1852, 1.0216] | 1709│1610 | 5 | -0.5012 | [-1.3024, 0.3001] | [-1.5824, 0.8665] | 1242│1163 |

Note: (i) All estimates are computed using a triangular Kernel and local linear regression. (ii) The bandwidth in the first line is the MSE optimal bandwidth.  

Table S12. Fuzzy RD estimates at different bandwidths – CESD Scale – By gender at age 65

| Male |  |  |  |  | Female |  |  |  |  |
| --- | --- | --- | --- | --- | --- | --- | --- | --- | --- |
| Bandwidths | Estimate | 95% CI (Conventional) | 95% CI (Robust) | Obs L│R | Bandwidths | Estimate | 95% CI (Conventional) | 95% CI (Robust) | Obs L│R |
| 2.6 | -0.082 | [-7.3509, 7.187] | [-12.7522, 9.3024] | 1636│1583 | 3.9 | 0.5681 | [-13.562, 14.6982] | [-13.3049, 28.2534] | 1256│1168 |
| 1 | -0.0579 | [-10.8867, 10.7708] | [-20.6684, 15.7711] | 1636│1583 | 1 | 1989.868 | [-535144.3067, 539124.0423] | [-679437.188, 1089168.5474] | 1256│1168 |
| 1.5 | -1.4327 | [-9.942, 7.0766] | [-12.8373, 14.2046] | 1636│1583 | 1.5 | 19.4501 | [-74.1878, 113.0881] | [-53.4963, 242.2248] | 1256│1168 |
| 2 | -0.8207 | [-8.9096, 7.2683] | [-14.3986, 10.2217] | 1636│1583 | 2 | 8.1348 | [-23.8998, 40.1694] | [-18.9281, 78.0463] | 1256│1168 |
| 2.5 | -0.1113 | [-7.5306, 7.3081] | [-13.1124, 9.3676] | 1636│1583 | 2.5 | 4.5466 | [-15.7969, 24.89] | [-14.7806, 45.7037] | 1256│1168 |
| 3 | 0.3831 | [-6.6167, 7.3829] | [-12.3606, 8.8622] | 1636│1583 | 3 | 3.0557 | [-13.4324, 19.5437] | [-13.9327, 34.9419] | 1256│1168 |
| 3.5 | 0.6762 | [-5.6274, 6.9798] | [-10.6276, 8.4702] | 1636│1583 | 3.5 | 1.3013 | [-13.7275, 16.3302] | [-12.8618, 31.4481] | 1256│1168 |
| 4 | 0.7978 | [-5.0538, 6.6493] | [-9.4462, 8.2264] | 1636│1583 | 4 | 0.5048 | [-13.3833, 14.3929] | [-13.5265, 27.2934] | 1256│1168 |
| 4.5 | 1.4381 | [-4.2027, 7.0788] | [-9.4697, 7.4854] | 1636│1583 | 4.5 | 0.8086 | [-12.9747, 14.5919] | [-16.163, 24.3291] | 1256│1168 |
| 5 | 2.0571 | [-3.4293, 7.5434] | [-9.3071, 7.099] | 1636│1583 | 5 | 0.8445 | [-12.4187, 14.1077] | [-16.5065, 22.3724] | 1256│1168 |

Note: (i) All estimates are computed using a triangular Kernel and local linear regression. (ii) The bandwidth in the first line is the MSE optimal bandwidth.  

Table S13. Fuzzy RD estimates at different bandwidths – the CHARLS cognitive battery – By gender at age 65

| Male |  |  |  |  | Female |  |  |  |  |
| --- | --- | --- | --- | --- | --- | --- | --- | --- | --- |
| Bandwidths | Estimate | 95% CI (Conventional) | 95% CI (Robust) | Obs L│R | Bandwidths | Estimate | 95% CI (Conventional) | 95% CI (Robust) | Obs L│R |
| 3.1 | -1.9217 | [-4.4678, 0.6244] | [-6.4228, 0.6868] | 1557│1484 | 3.7 | 2.3324 | [-2.8797, 7.5444] | [-7.7233, 7.7472] | 1176│1057 |
| 1 | -2.5408 | [-6.4288, 1.3471] | [-10.8689, 1.6421] | 1557│1484 | 1 | -6.3453 | [-28.7836, 16.093] | [-54.5053, 18.1423] | 1176│1057 |
| 1.5 | -2.5718 | [-5.7876, 0.6439] | [-7.2529, 1.8389] | 1557│1484 | 1.5 | -0.7421 | [-7.8411, 6.3568] | [-19.338, 2.0446] | 1176│1057 |
| 2 | -2.5651 | [-5.711, 0.5808] | [-6.883, 1.6835] | 1557│1484 | 2 | 0.2890 | [-5.2399, 5.8179] | [-11.1598, 5.0983] | 1176│1057 |
| 2.5 | -2.2481 | [-5.1015, 0.6053] | [-6.7903, 1.0153] | 1557│1484 | 2.5 | 1.1956 | [-3.9189, 6.3102] | [-9.2713, 5.7418] | 1176│1057 |
| 3 | -1.9824 | [-4.6022, 0.6373] | [-6.5076, 0.7776] | 1557│1484 | 3 | 1.8348 | [-3.1756, 6.8452] | [-8.307, 6.5137] | 1176│1057 |
| 3.5 | -1.6606 | [-3.9349, 0.6136] | [-6.0573, 0.3729] | 1557│1484 | 3.5 | 2.2578 | [-2.9258, 7.4413] | [-7.9753, 7.4068] | 1176│1057 |
| 4 | -1.6141 | [-3.7403, 0.5121] | [-5.4225, 0.6445] | 1557│1484 | 4 | 2.2048 | [-2.8303, 7.2398] | [-6.6006, 8.2788] | 1176│1057 |
| 4.5 | -1.6708 | [-3.7322, 0.3907] | [-4.9893, 0.9382] | 1557│1484 | 4.5 | 1.9955 | [-2.9831, 6.9741] | [-5.6989, 8.9342] | 1176│1057 |
| 5 | -1.6813 | [-3.6595, 0.2968] | [-4.7754, 0.9631] | 1557│1484 | 5 | 1.9403 | [-2.8668, 6.7475] | [-5.2055, 8.8495] | 1176│1057 |

Note: (i) All estimates are computed using a triangular Kernel and local linear regression. (ii) The bandwidth in the first line is the MSE optimal bandwidth.  

Table S14. Fuzzy RD estimates at different bandwidths – ADL – By gender at age 65

| Male |  |  |  |  | Female |  |  |  |  |
| --- | --- | --- | --- | --- | --- | --- | --- | --- | --- |
| Bandwidths | Estimate | 95% CI (Conventional) | 95% CI (Robust) | Obs L│R | Bandwidths | Estimate | 95% CI (Conventional) | 95% CI (Robust) | Obs L│R |
| 2.9 | -0.0172 | [-0.6949, 0.6605] | [-0.6117, 1.0061] | 1820│1672 | 3.1 | 0.2526 | [-0.5537, 1.0589] | [-0.3121, 2.4776] | 1302│1251 |
| 1 | 0.8498 | [8e-04, 1.6988] | [-0.1631, 2.4864] | 1820│1672 | 1 | 6.8682 | [-91.4755, 105.2118] | [-98.6259, 226.6819] | 1302│1251 |
| 1.5 | 0.2184** | [-0.3892, 0.8261] | [0.3275, 2.3098] | 1820│1672 | 1.5 | 1.3199 | [-3.2060, 5.8458] | [-2.5456, 11.7096] | 1302│1251 |
| 2 | 0.0233 | [-0.6377, 0.6843] | [-0.0828, 1.7340] | 1820│1672 | 2 | 0.9155 | [-1.2241, 3.0550] | [-1.0583, 5.5888] | 1302│1251 |
| 2.5 | -0.0312 | [-0.7048, 0.6425] | [-0.4369, 1.2612] | 1820│1672 | 2.5 | 0.4704 | [-0.6153, 1.5561] | [-0.2998, 3.3916] | 1302│1251 |
| 3 | -0.0146 | [-0.6913, 0.6621] | [-0.6326, 0.9668] | 1820│1672 | 3 | 0.2702 | [-0.5457, 1.0861] | [-0.2786, 2.5797] | 1302│1251 |
| 3.5 | 0.0044 | [-0.6244, 0.6333] | [-0.6755, 0.7793] | 1820│1672 | 3.5 | 0.208 | [-0.5917, 1.0078] | [-0.4400, 2.1104] | 1302│1251 |
| 4 | 0.0584 | [-0.5351, 0.6518] | [-0.7399, 0.6431] | 1820│1672 | 4 | 0.1331 | [-0.6438, 0.9100] | [-0.4316, 1.7563] | 1302│1251 |
| 4.5 | 0.092 | [-0.4859, 0.6699] | [-0.7421, 0.6405] | 1820│1672 | 4.5 | 0.1339 | [-0.6551, 0.9228] | [-0.5793, 1.4579] | 1302│1251 |
| 5 | 0.0831 | [-0.4851, 0.6513] | [-0.6824, 0.7107] | 1820│1672 | 5 | 0.1016 | [-0.6642, 0.8674] | [-0.5672, 1.3152] | 1302│1251 |

Note: (i) All estimates are computed using a triangular Kernel and local linear regression. (ii) The bandwidth in the first line is the MSE optimal bandwidth.  

(iii)  ** indicate significance level at 5%.

Table S15. Fuzzy RD estimates at different bandwidths – IADL Scale – By gender at age 65

| Male |  |  |  |  | Female |  |  |  |  |
| --- | --- | --- | --- | --- | --- | --- | --- | --- | --- |
| Bandwidths | Estimate | 95% CI (Conventional) | 95% CI (Robust) | Obs L│R | Bandwidths | Estimate | 95% CI (Conventional) | 95% CI (Robust) | Obs L│R |
| 2.2 | -0.7394 | [-1.8003, 0.3215] | [-1.8990, 0.9691] | 1554│1651 | 3.1 | 0.3889 | [-0.5219, 1.2998] | [-0.6503, 2.0774] | 1166│1221 |
| 1 | -0.3256 | [-1.1992, 0.5481] | [-2.3972, 1.0077] | 1554│1651 | 1 | 1.2933 | [-4.0734, 6.6600] | [-4.6470, 13.6277] | 1166│1221 |
| 1.5 | -0.6629 | [-1.5841, 0.2584] | [-1.5594, 1.3068] | 1554│1651 | 1.5 | 0.5481 | [-1.1858, 2.2820] | [-0.9827, 4.1595] | 1166│1221 |
| 2 | -0.7432 | [-1.7706, 0.2842] | [-1.7746, 1.0596] | 1554│1651 | 2 | 0.5665 | [-0.8342, 1.9673] | [-1.0734, 2.8577] | 1166│1221 |
| 2.5 | -0.6683 | [-1.633, 0.2965] | [-1.9716, 0.5914] | 1554│1651 | 2.5 | 0.4947 | [-0.5896, 1.5790] | [-0.9009, 2.3279] | 1166│1221 |
| 3 | -0.5053 | [-1.3506, 0.3401] | [-1.9412, 0.2194] | 1554│1651 | 3 | 0.4137 | [-0.5137, 1.3410] | [-0.6954, 2.1028] | 1166│1221 |
| 3.5 | -0.3301* | [-1.0309, 0.3706] | [-1.7619, -0.0159] | 1554│1651 | 3.5 | 0.2610 | [-0.5788, 1.1008] | [-0.4297, 1.9650] | 1166│1221 |
| 4 | -0.1791* | [-0.8021, 0.4439] | [-1.6096, -0.0626] | 1554│1651 | 4 | 0.1011 | [-0.6704, 0.8725] | [-0.2503, 1.8032] | 1166│1221 |
| 4.5 | -0.0825 | [-0.6683, 0.5033] | [-1.4444, 0.0337] | 1554│1651 | 4.5 | 0.0567 | [-0.7244, 0.8377] | [-0.4086, 1.5801] | 1166│1221 |
| 5 | -0.059 | [-0.6234, 0.5054] | [-1.2519, 0.2028] | 1554│1651 | 5 | 0.0103 | [-0.7524, 0.7730] | [-0.4789, 1.4165] | 1166│1221 |

Note: (i) All estimates are computed using a triangular Kernel and local linear regression. (ii) The bandwidth in the first line is the MSE optimal bandwidth.  

(iii) * indicate significance level at 10%. 

Table S16. Fuzzy RD estimates at different bandwidths – Total recall score – By gender at age 65

| Male |  |  |  |  | Female |  |  |  |  |
| --- | --- | --- | --- | --- | --- | --- | --- | --- | --- |
| Bandwidths | Estimate | 95% CI (Conventional) | 95% CI (Robust) | Obs L│R | Bandwidths | Estimate | 95% CI (Conventional) | 95% CI (Robust) | Obs L│R |
| 3 | -2.0079 | [-7.3580, 3.3422] | [-7.2852, 7.9391] | 1587│1533 | 3 | 8.8388 | [-5.0985, 22.7760] | [-12.6402, 29.1719] | 1216│1124 |
| 1 | -0.8132 | [-7.2062, 5.5798] | [-14.4197, 6.7452] | 1587│1533 | 1 | -1.0791 | [-23.2813, 21.1231] | [-50.8273, 22.1633] | 1216│1124 |
| 1.5 | -0.9201 | [-6.3528, 4.5126] | [-9.2897, 6.3735] | 1587│1533 | 1.5 | 6.6848 | [-13.1517, 26.5213] | [-33.8746, 26.9177] | 1216│1124 |
| 2 | -0.6231 | [-6.0736, 4.8275] | [-8.3733, 6.6697] | 1587│1533 | 2 | 9.0889 | [-8.6784, 26.8562] | [-22.6625, 30.6305] | 1216│1124 |
| 2.5 | -1.6549 | [-7.0621, 3.7523] | [-6.8965, 8.2822] | 1587│1533 | 2.5 | 8.9127 | [-6.1361, 23.9614] | [-15.5443, 29.3264] | 1216│1124 |
| 3 | -2.0079 | [-7.3580, 3.3422] | [-7.2852, 7.9391] | 1587│1533 | 3 | 8.8388 | [-5.0985, 22.7760] | [-12.6402, 29.1719] | 1216│1124 |
| 3.5 | -2.0603 | [-6.9143, 2.7936] | [-7.5372, 6.3253] | 1587│1533 | 3.5 | 9.1657 | [-4.8879, 23.2193] | [-13.0641, 29.0291] | 1216│1124 |
| 4 | -2.2052 | [-6.7541, 2.3437] | [-7.5747, 5.4305] | 1587│1533 | 4 | 8.4478 | [-4.1705, 21.0661] | [-9.1042, 28.4361] | 1216│1124 |
| 4.5 | -2.2378 | [-6.6006, 2.1250] | [-7.7773, 4.7588] | 1587│1533 | 4.5 | 7.6278 | [-3.9689, 19.2245] | [-6.6528, 27.5932] | 1216│1124 |
| 5 | -2.2999 | [-6.5045, 1.9048] | [-7.7460, 4.3904] | 1587│1533 | 5 | 6.8328 | [-3.5750, 17.2405] | [-4.4282, 26.0819] | 1216│1124 |

Note: (i) All estimates are computed using a triangular Kernel and local linear regression. (ii) The bandwidth in the first line is the MSE optimal bandwidth.  

Table S17. Fuzzy RD estimates at different bandwidths – Self-reported health – By education at age 65

| Low Educated |  |  |  |  | High Educated |  |  |  |  |
| --- | --- | --- | --- | --- | --- | --- | --- | --- | --- |
| Bandwidths | Estimate | 95% CI (Conventional) | 95% CI (Robust) | Obs L│R | Bandwidths | Estimate | 95% CI (Conventional) | 95% CI (Robust) | Obs L│R |
| 3.1 | 0.1037 | [-0.4305, 0.6379] | [-0.5362, 0.9856] | 1075│2270 | 4.2 | 0.2610 | [-0.4065, 0.9286] | [-0.4855, 1.4509] | 1274│1184 |
| 1 | 0.2323 | [-0.3605, 0.8251] | [-0.2917, 1.5335] | 1075│2270 | 1 | 0.2480 | [-0.4073, 0.9033] | [-0.6388, 1.0363] | 1274│1184 |
| 1.5 | 0.1501 | [-0.3558, 0.6559] | [-0.4378, 0.9937] | 1075│2270 | 1.5 | 0.4703 | [-0.3518, 1.2923] | [-1.1477, 1.1277] | 1274│1184 |
| 2 | 0.1540 | [-0.3718, 0.6799] | [-0.5391, 0.9351] | 1075│2270 | 2 | 0.4798 | [-0.2974, 1.2571] | [-0.6928, 1.4444] | 1274│1184 |
| 2.5 | 0.1467 | [-0.3841, 0.6776] | [-0.5451, 0.9530] | 1075│2270 | 2.5 | 0.3916 | [-0.3080, 1.0913] | [-0.4478, 1.5134] | 1274│1184 |
| 3 | 0.1083 | [-0.4265, 0.6432] | [-0.5313, 0.9899] | 1075│2270 | 3 | 0.3398 | [-0.3586, 1.0381] | [-0.4353, 1.5570] | 1274│1184 |
| 3.5 | 0.1111 | [-0.4034, 0.6257] | [-0.5685, 0.9045] | 1075│2270 | 3.5 | 0.2961 | [-0.3723, 0.9645] | [-0.4221, 1.4986] | 1274│1184 |
| 4 | 0.1072 | [-0.3775, 0.5919] | [-0.5455, 0.8506] | 1075│2270 | 4 | 0.2694 | [-0.3976, 0.9364] | [-0.4680, 1.4607] | 1274│1184 |
| 4.5 | 0.1256 | [-0.3394, 0.5906] | [-0.5617, 0.7840] | 1075│2270 | 4.5 | 0.2455 | [-0.4083, 0.8992] | [-0.4838, 1.4162] | 1274│1184 |
| 5 | 0.1153 | [-0.3275, 0.5580] | [-0.5044, 0.7823] | 1075│2270 | 5 | 0.2248 | [-0.4142, 0.8637] | [-0.4988, 1.3608] | 1274│1184 |

Note: (i) All estimates are computed using a triangular Kernel and local linear regression. (ii) The bandwidth in the first line is the MSE optimal bandwidth.  

Table S18. Fuzzy RD estimates at different bandwidths – Life satisfaction – By education at age 65

| Low Educated |  |  |  |  | High Educated |  |  |  |  |
| --- | --- | --- | --- | --- | --- | --- | --- | --- | --- |
| Bandwidths | Estimate | 95% CI (Conventional) | 95% CI (Robust) | Obs L│R | Bandwidths | Estimate | 95% CI (Conventional) | 95% CI (Robust) | Obs L│R |
| 3 | 0.0183 | [-0.3591, 0.3956] | [-0.1869, 0.7948] | 1339│1905 | 2.7 | -0.1490 | [-0.5675, 0.2694] | [-0.9286, 0.3162] | 1529│842 |
| 1 | 0.3056 | [-0.7440, 1.3553] | [-1.2471, 2.5727] | 1339│1905 | 1 | -0.6085 | [-2.5244, 1.3073] | [-3.3045, 2.7108] | 1529│842 |
| 1.5 | 0.3073 | [-0.3342, 0.9488] | [-0.6475, 1.3159] | 1339│1905 | 1.5 | -0.2845 | [-1.0476, 0.4786] | [-1.6916, 0.5533] | 1529│842 |
| 2 | 0.2137 | [-0.3150, 0.7424] | [-0.3664, 1.1281] | 1339│1905 | 2 | -0.2233 | [-0.7472, 0.3006] | [-1.1220, 0.4049] | 1529│842 |
| 2.5 | 0.1105 | [-0.3112, 0.5322] | [-0.1944, 0.9418] | 1339│1905 | 2.5 | -0.1643 | [-0.6009, 0.2723] | [-0.9673, 0.3224] | 1529│842 |
| 3 | 0.0183 | [-0.3591, 0.3956] | [-0.1869, 0.7948] | 1339│1905 | 3 | -0.1194 | [-0.5183, 0.2795] | [-0.8935, 0.3026] | 1529│842 |
| 3.5 | -0.0263 | [-0.3746, 0.3220] | [-0.2397, 0.6604] | 1339│1905 | 3.5 | -0.0838 | [-0.4553, 0.2877] | [-0.8269, 0.3009] | 1529│842 |
| 4 | -0.0660 | [-0.3963, 0.2643] | [-0.2670, 0.5971] | 1339│1905 | 4 | -0.0885 | [-0.4466, 0.2696] | [-0.7254, 0.3564] | 1529│842 |
| 4.5 | -0.1045 | [-0.4309, 0.2220] | [-0.3011, 0.5706] | 1339│1905 | 4.5 | -0.1064 | [-0.4646, 0.2519] | [-0.6622, 0.4100] | 1529│842 |
| 5 | -0.1304 | [-0.4526, 0.1918] | [-0.3435, 0.5286] | 1339│1905 | 5 | -0.1246 | [-0.4803, 0.2311] | [-0.6222, 0.4386] | 1529│842 |

Note: (i) All estimates are computed using a triangular Kernel and local linear regression. (ii) The bandwidth in the first line is the MSE optimal bandwidth.  

Table S19. Fuzzy RD estimates at different bandwidths – CESD Scale – By education at age 65

| Low Educated |  |  |  |  | High Educated |  |  |  |  |
| --- | --- | --- | --- | --- | --- | --- | --- | --- | --- |
| Bandwidths | Estimate | 95% CI (Conventional) | 95% CI (Robust) | Obs L│R | Bandwidths | Estimate | 95% CI (Conventional) | 95% CI (Robust) | Obs L│R |
| 3.1 | 4.5933 | [-4.5766, 13.7633] | [-7.2359, 21.112] | 1355│1909 | 3.3 | -0.7378 | [-7.7782, 6.3026] | [-11.9882, 8.1417] | 1537│842 |
| 1 | 12.8922 | [-25.8925, 51.6769] | [-34.3597, 104.1168] | 1355│1909 | 1 | 1.0046 | [-23.2886, 25.2979] | [-37.1072, 35.1851] | 1537│842 |
| 1.5 | 6.3741 | [-10.2409, 22.9890] | [-11.1849, 43.3931] | 1355│1909 | 1.5 | -2.9237 | [-16.0004, 10.1531] | [-19.9791, 19.9183] | 1537│842 |
| 2 | 6.5653 | [-7.8863, 21.0169] | [-13.9418, 30.8501] | 1355│1909 | 2 | -2.0343 | [-11.1765, 7.1079] | [-16.2145, 10.2977] | 1537│842 |
| 2.5 | 5.4788 | [-5.6337, 16.5913] | [-8.9005, 25.4034] | 1355│1909 | 2.5 | -1.3118 | [-9.0919, 6.4682] | [-13.8347, 8.3703] | 1537│842 |
| 3 | 4.6340 | [-4.7347, 14.0026] | [-7.2364, 21.7181] | 1355│1909 | 3 | -0.7203 | [-7.9968, 6.5561] | [-12.8717, 7.8984] | 1537│842 |
| 3.5 | 3.7120 | [-4.4049, 11.8289] | [-5.2911, 19.6121] | 1355│1909 | 3.5 | -0.6621 | [-7.6097, 6.2856] | [-11.7662, 8.1211] | 1537│842 |
| 4 | 2.6811 | [-4.5606, 9.9229] | [-4.129, 17.9318] | 1355│1909 | 4 | -0.0847 | [-6.8103, 6.6410] | [-11.9001, 7.3165] | 1537│842 |
| 4.5 | 2.6002 | [-4.3178, 9.5182] | [-5.1024, 15.88] | 1355│1909 | 4.5 | 0.8457 | [-5.9077, 7.5991] | [-12.6144, 6.7079] | 1537│842 |
| 5 | 2.6665 | [-4.0105, 9.3435] | [-5.7707, 14.3222] | 1355│1909 | 5 | 1.4520 | [-5.2827, 8.1866] | [-12.4272, 6.9578] | 1537│842 |

Note: (i) All estimates are computed using a triangular Kernel and local linear regression. (ii) The bandwidth in the first line is the MSE optimal bandwidth.  

Table S20. Fuzzy RD estimates at different bandwidths – the CHARLS cognitive battery – By education at age 65

| Low Educated |  |  |  |  | High Educated |  |  |  |  |
| --- | --- | --- | --- | --- | --- | --- | --- | --- | --- |
| Bandwidths | Estimate | 95% CI (Conventional) | 95% CI (Robust) | Obs L│R | Bandwidths | Estimate | 95% CI (Conventional) | 95% CI (Robust) | Obs L│R |
| 2.9 | -3.2411* | [-6.9105, 0.4283] | [-10.835, -0.0711] | 1241│1720 | 2.6 | 1.3649 | [-0.9414, 3.6711] | [-3.4222, 3.2389] | 1492│821 |
| 1 | -5.7041 | [-18.0135, 6.6054] | [-35.2219, 3.9051] | 1241│1720 | 1 | -2.9704 | [-9.9197, 3.9789] | [-21.8843, 0.5281] | 1492│821 |
| 1.5 | -4.6958 | [-11.5427, 2.1512] | [-17.0152, 2.9751] | 1241│1720 | 1.5 | 0.2724 | [-2.6939, 3.2387] | [-7.8675, 0.6076] | 1492│821 |
| 2 | -4.7982 | [-11.0826, 1.4862] | [-14.6964, 3.3361] | 1241│1720 | 2 | 0.8730 | [-1.5763, 3.3223] | [-4.2167, 2.5491] | 1492│821 |
| 2.5 | -3.8137 | [-8.2871, 0.6598] | [-12.3314, 0.7000] | 1241│1720 | 2.5 | 1.2712 | [-1.0320, 3.5743] | [-3.4368, 3.1586] | 1492│821 |
| 3 | -3.1255* | [-6.6517, 0.4007] | [-10.5419, -0.1733] | 1241│1720 | 3 | 1.6374 | [-0.6708, 3.9456] | [-3.1943, 3.6122] | 1492│821 |
| 3.5 | -2.5106* | [-5.3989, 0.3778] | [-9.1558, -0.6713] | 1241│1720 | 3.5 | 1.707 | [-0.4992, 3.9132] | [-2.5599, 4.0355] | 1492│821 |
| 4 | -2.3161* | [-4.9664, 0.3342] | [-7.9861, -0.2415] | 1241│1720 | 4 | 1.6204 | [-0.4793, 3.7200] | [-1.8970, 4.3963] | 1492│821 |
| 4.5 | -2.2872 | [-4.8512, 0.2768] | [-7.2322, 0.2221] | 1241│1720 | 4.5 | 1.4863 | [-0.5643, 3.5368] | [-1.4694, 4.6766] | 1492│821 |
| 5 | -2.0955 | [-4.5032, 0.3121] | [-6.8579, 0.1068] | 1241│1720 | 5 | 1.3075 | [-0.6699, 3.2849] | [-1.1008, 4.8371] | 1492│821 |

Note: (i) All estimates are computed using a triangular Kernel and local linear regression. (ii) The bandwidth in the first line is the MSE optimal bandwidth.  

(iii) * indicate significance level at 10%. 

Table S21. Fuzzy RD estimates at different bandwidths – ADL – By education at age 65

| Low Educated |  |  |  |  | High Educated |  |  |  |  |
| --- | --- | --- | --- | --- | --- | --- | --- | --- | --- |
| Bandwidths | Estimate | 95% CI (Conventional) | 95% CI (Robust) | Obs L│R | Bandwidths | Estimate | 95% CI (Conventional) | 95% CI (Robust) | Obs L│R |
| 3.7 | 0.0854 | [-0.3663, 0.5371] | [-0.2039, 0.9728] | 1454│2012 | 4.1 | 0.0179 | [-0.6804, 0.7161] | [-0.9862, 0.7475] | 1668│911 |
| 1 | 1.6791* | [-1.8872, 5.2454] | [0.5349, 12.1784] | 1454│2012 | 1 | 1.3315 | [-1.6804, 4.3433] | [-1.8291, 7.7936] | 1668│911 |
| 1.5 | 0.6873* | [-0.4134, 1.7880] | [0.4428, 3.8974] | 1454│2012 | 1.5 | 0.1013* | [-0.7889, 0.9916] | [0.0199, 2.9597] | 1668│911 |
| 2 | 0.5218* | [-0.3230, 1.3666] | [0.0825, 2.6875] | 1454│2012 | 2 | -0.0647 | [-0.8285, 0.6991] | [-0.4563, 1.4944] | 1668│911 |
| 2.5 | 0.2221* | [-0.3131, 0.7574] | [0.1621, 1.9872] | 1454│2012 | 2.5 | -0.0600 | [-0.7875, 0.6674] | [-0.7269, 0.9763] | 1668│911 |
| 3 | 0.1170 | [-0.3558, 0.5899] | [-0.0411, 1.4454] | 1454│2012 | 3 | -0.0490 | [-0.7869, 0.6889] | [-0.8529, 0.8797] | 1668│911 |
| 3.5 | 0.0990 | [-0.3537, 0.5516] | [-0.1843, 1.0557] | 1454│2012 | 3.5 | -0.0400 | [-0.7668, 0.6867] | [-0.9031, 0.8376] | 1668│911 |
| 4 | 0.0909 | [-0.3630, 0.5448] | [-0.2640, 0.8320] | 1454│2012 | 4 | 0.0090 | [-0.6921, 0.7102] | [-0.9762, 0.7520] | 1668│911 |
| 4.5 | 0.1088 | [-0.3588, 0.5764] | [-0.3387, 0.7137] | 1454│2012 | 4.5 | 0.0500 | [-0.6371, 0.7371] | [-1.0103, 0.7512] | 1668│911 |
| 5 | 0.1029 | [-0.3726, 0.5784] | [-0.3473, 0.6905] | 1454│2012 | 5 | 0.0405 | [-0.6309, 0.7120] | [-0.9418, 0.8432] | 1668│911 |

Note: (i) All estimates are computed using a triangular Kernel and local linear regression. (ii) The bandwidth in the first line is the MSE optimal bandwidth.  

(iii) * indicate significance level at 10%. 

Table S22. Fuzzy RD estimates at different bandwidths – IADL Scale – By education at age 65

| Low Educated |  |  |  |  | High Educated |  |  |  |  |
| --- | --- | --- | --- | --- | --- | --- | --- | --- | --- |
| Bandwidths | Estimate | 95% CI (Conventional) | 95% CI (Robust) | Obs L│R | Bandwidths | Estimate | 95% CI (Conventional) | 95% CI (Robust) | Obs L│R |
| 2.6 | -0.0380 | [-0.7084, 0.6323] | [-1.1225, 0.7310] | 1251│1968 | 3.7 | -0.2889 | [-0.9966, 0.4189] | [-1.3405, 0.4968] | 1469│904 |
| 1 | 0.0685 | [-1.5592, 1.6961] | [-2.3346, 3.6564] | 1251│1968 | 1 | 0.0453 | [-1.0647, 1.1553] | [-1.9032, 1.9639] | 1469│904 |
| 1.5 | -0.2811 | [-1.2231, 0.6609] | [-1.4205, 1.600] | 1251│1968 | 1.5 | -0.3975 | [-1.3981, 0.6032] | [-1.2051, 1.4696] | 1469│904 |
| 2 | -0.1227 | [-0.9755, 0.7300] | [-1.452, 0.9249] | 1251│1968 | 2 | -0.4279 | [-1.3060, 0.4503] | [-1.3003, 0.8383] | 1469│904 |
| 2.5 | -0.0584 | [-0.7555, 0.6387] | [-1.1584, 0.7759] | 1251│1968 | 2.5 | -0.3697 | [-1.1469, 0.4076] | [-1.3858, 0.5412] | 1469│904 |
| 3 | 0.0207 | [-0.5876, 0.6291] | [-0.9583, 0.6423] | 1251│1968 | 3 | -0.3143 | [-1.0568, 0.4282] | [-1.3948, 0.4957] | 1469│904 |
| 3.5 | 0.0595 | [-0.4928, 0.6117] | [-0.7877, 0.5906] | 1251│1968 | 3.5 | -0.2992 | [-1.0156, 0.4173] | [-1.3523, 0.5027] | 1469│904 |
| 4 | 0.0611 | [-0.4673, 0.5896] | [-0.6866, 0.6065] | 1251│1968 | 4 | -0.2363 | [-0.9139, 0.4413] | [-1.3551, 0.4039] | 1469│904 |
| 4.5 | 0.0730 | [-0.4581, 0.6041] | [-0.6711, 0.6257] | 1251│1968 | 4.5 | -0.1658 | [-0.8184, 0.4868] | [-1.3517, 0.3587] | 1469│904 |
| 5 | 0.0620 | [-0.4659, 0.5899] | [-0.6128, 0.6763] | 1251│1968 | 5 | -0.1538 | [-0.7917, 0.4840] | [-1.2552, 0.4553] | 1469│904 |

Note: (i) All estimates are computed using a triangular Kernel and local linear regression. (ii) The bandwidth in the first line is the MSE optimal bandwidth.  

Table S23. Fuzzy RD estimates at different bandwidths – Total recall score – By education at age 65

| Low Educated |  |  |  |  | High Educated |  |  |  |  |
| --- | --- | --- | --- | --- | --- | --- | --- | --- | --- |
| Bandwidths | Estimate | 95% CI (Conventional) | 95% CI (Robust) | Obs L│R | Bandwidths | Estimate | 95% CI (Conventional) | 95% CI (Robust) | Obs L│R |
| 2.7 | 2.1123 | [-4.0938, 8.3185] | [-6.7440, 10.7301] | 1294│1823 | 2.6 | 2.5517 | [-2.0053, 7.1088] | [-5.3186, 7.6225] | 1509│834 |
| 1 | 0.9066 | [-10.1851, 11.9984] | [-13.3454, 19.8756] | 1294│1823 | 1 | -8.1809* | [-29.0404, 12.6787] | [-73.1642, -5.0546] | 1509│834 |
| 1.5 | 0.9129 | [-6.9476, 8.7733] | [-9.5736, 12.2866] | 1294│1823 | 1.5 | 0.8105* | [-5.7406, 7.3615] | [-20.8791, -2.1004] | 1509│834 |
| 2 | 2.3885 | [-5.3057, 10.0826] | [-10.4985, 10.5675] | 1294│1823 | 2 | 2.6453 | [-2.6004, 7.8910] | [-9.7701, 5.0061] | 1509│834 |
| 2.5 | 2.1451 | [-4.4684, 8.7587] | [-7.4148, 11.0517] | 1294│1823 | 2.5 | 2.4855 | [-2.0986, 7.0696] | [-5.4566, 7.5087] | 1509│834 |
| 3 | 2.1496 | [-3.7939, 8.0931] | [-6.2970, 10.5918] | 1294│1823 | 3 | 2.6682 | [-1.8045, 7.1410] | [-4.6663, 8.0908] | 1509│834 |
| 3.5 | 1.9742 | [-3.3313, 7.2796] | [-5.2143, 9.9944] | 1294│1823 | 3.5 | 2.8003 | [-1.5400, 7.1406] | [-4.3576, 8.0955] | 1509│834 |
| 4 | 1.8216 | [-3.0169, 6.6601] | [-4.6029, 9.2828] | 1294│1823 | 4 | 2.5137 | [-1.6700, 6.6975] | [-3.3203, 8.6774] | 1509│834 |
| 4.5 | 1.7611 | [-2.8101, 6.3322] | [-4.4059, 8.7394] | 1294│1823 | 4.5 | 2.1898 | [-1.9680, 6.3475] | [-2.8023, 9.1130] | 1509│834 |
| 5 | 1.5275 | [-2.8065, 5.8616] | [-3.8052, 8.6422] | 1294│1823 | 5 | 2.0076 | [-2.1176, 6.1328] | [-2.7400, 9.1110] | 1509│834 |

Note: (i) All estimates are computed using a triangular Kernel and local linear regression. (ii) The bandwidth in the first line is the MSE optimal bandwidth.  

Table S24. Nonparametric fuzzy regression discontinuity (RD) estimates—Gender heterogeneity at age 65

|  | Smoking |  | Drinking |  | Physical Activity |  |
| --- | --- | --- | --- | --- | --- | --- |
|  | Male | Female | Male | Female | Male | Female |
| MSE Optimal |  |  |  |  |  |  |
| Bandwidth | 2.7 | 3.3 | 2.6 | 3.4 | 3.1 | 3.4 |
| Point Estimate | 0.3905 | 0.0198 | 0.3367 | -0.4098 | -0.1775 | 0.0838 |
| Conventional 95% CI | [-0.3478, 1.1289] | [-0.4446, 0.4841] | [-0.3981, 1.0714] | [-1.6618, 0.8423] | [-0.487, 0.1321] | [-0.2604, 0.4281] |
| Robust 95% CI | 0.3983 | 0.0228 | 0.3159 | -0.3593 | -0.1783 | 0.0661 |
| Effective obs L│R | [-0.4624, 1.2591] | [-0.5419, 0.5875] | [-0.5524, 1.1842] | [-1.8583, 1.1398] | [-0.5349, 0.1782] | [-0.3378, 0.47] |
| CER Optimal | 455│401 | 453│439 | 495│454 | 452│441 | 311│341 | 315│293 |
| Bandwidth |  |  |  |  |  |  |
| Point Estimate | 1.8 | 2.2 | 1.8 | 2.3 | 2.1 | 2.4 |
| Conventional 95% CI | 0.0633 | 0.0205 | 0.0207 | -0.2929 | -0.0846 | -0.0186 |
| Robust 95% CI | [-0.7083, 0.8349] | [-0.558, 0.599] | [-0.7799, 0.8213] | [-1.7301, 1.1444] | [-0.417, 0.2479] | [-0.3319, 0.2946] |
| Effective obs L│R | 0.0714 | 0.0218 | 0.0107 | -0.2691 | -0.0856 | -0.0222 |
| Obs L│R | [-0.7487, 0.8915] | [-0.6005, 0.6441] | [-0.8456, 0.867] | [-1.8202, 1.2819] | [-0.44, 0.2687] | [-0.3608, 0.3165] |
| Note: (i) All estimates are computed using a triangular Kernel and nearest neighbor heteroskedasticity-robust variance estimator as suggested by CCT framework. (ii) Effective number of observations depend on the size of the data-driven selected bandwidth. All of the p-values > 0.05. |  |  |  |  |  |  |

Table S25. Nonparametric fuzzy regression discontinuity (RD) estimates—Education heterogeneity at age 65

|  | Smoking |  | Drinking |  |  | Physical Activity |  |  |  |
| --- | --- | --- | --- | --- | --- | --- | --- | --- | --- |
|  | Low educated | High educated | Low educated | High educated |  | Low educated |  | High educated |  |
| MSE Optimal |  |  |  |  |  |  |  |  |  |
| Bandwidth | 3.3 | 3.8 | 2.6 |  | 3.9 | 2.9 | 3.6 |  |  |
| Point Estimate | 0.2023 | -0.2615 | -0.0300 |  | -0.4835 | -0.0367 | -0.0543 |  |  |
| Conventional 95% CI | [-0.4505, 0.855] | [-0.8219, 0.2989] | [-0.9143, 0.8544] |  | [-1.1796, 0.2126] | [-0.328, 0.2546] | [-0.2399, 0.1313] |  |  |
| Robust 95% CI | 0.2286 | -0.2918 | -0.1897 |  | -0.4268 | -0.0142 | -0.0936 |  |  |
| Effective obs L│R | [-0.5527, 1.01] | [-0.9539, 0.3703] | [-1.2407, 0.8613] |  | [-1.2607, 0.407] | [-0.3515, 0.3231] | [-0.3154, 0.1283] |  |  |
| CER Optimal | 478│572 | 559│407 | 429│474 |  | 625│453 | 258│317 | 373│284 |  |  |
| Bandwidth |  |  |  |  |  |  |  |  |  |
| Point Estimate | 2.2 | 2.6 | 1.7 |  | 2.6 | 2 | 2.5 |  |  |
| Conventional 95% CI | 0.0043 | -0.2025 | -0.3487 |  | -0.2791 | -0.0764 | -0.0212 |  |  |
| Robust 95% CI | [-0.916, 0.9246] | [-0.8393, 0.4343] | [-1.6528, 0.9555] |  | [-1.0221, 0.464] | [-0.4574, 0.3047] | [-0.1939, 0.1515] |  |  |
| Effective obs L│R | 0.0262 | -0.2174 | -0.44 |  | -0.2567 | -0.0638 | -0.0398 |  |  |
| Obs L│R | [-0.9651, 1.0175] | [-0.9039, 0.4692] | [-1.8367, 0.9567] |  | [-1.0603, 0.5469] | [-0.4725, 0.3449] | [-0.2308, 0.1512] |  |  |
| Note: (i) All estimates are computed using a triangular Kernel and nearest neighbor heteroskedasticity-robust variance estimator as suggested by CCT framework. (ii) Effective number of observations depend on the size of the data-driven selected bandwidth. All of the p-values > 0.05. |  |  |  |  |  |  |  |  |  |

Table S26. Formal falsification test for the validity of the Continuity-Based Analysis for predetermined Covariates

|  |  |  | Robust Inference |  |  |  |  |
| --- | --- | --- | --- | --- | --- | --- | --- |
| variables | MSE Optimal Bandwidth | RD Estimator | P-value | [Confidence Interval] | Effective L│R | L│R | Observations |
| Males | 1.843 | -0.07305 | 0.2808 | [-0.20642, 0.05989] | 606│590 | 3196│2926 | 6122 |
| Rural Hukou | 1.447 | 0.01287 | 0.8694 | [-0.12558, 0.14858] | 481│471 | 3196│2926 | 6122 |
| Education | 1.642 | 0.05858 | 0.3472 | [-0.07202, 0.20478] | 536│517 | 3196│2926 | 6122 |
| Divorced | 2.022 | -0.00073 | 0.9628 | [-0.03624, 0.03456] | 666│625 | 3196│2926 | 6122 |
| Married | 2.543 | 0.00209 | 0.9425 | [-0.07451, 0.08021] | 819│766 | 3196│2926 | 6122 |
| Never Married | 1.904 | -0.01302 | 0.1677 | [-0.03351, 0.00582] | 628│601 | 3196│2926 | 6122 |
| Partnered | 2.576 | -0.01338 | 0.6344 | [-0.07024, 0.04281] | 819│766 | 3196│2926 | 6122 |
| Separated | 1.797 | 0.00701 | 0.3065 | [-0.00701, 0.02231] | 558│539 | 3196│2926 | 6122 |
| Widowed | 2.535 | 0.01591 | 0.4839 | [-0.02894, 0.0611] | 819│766 | 3196│2926 | 6122 |
| Notes: RD estimated using local polynomial estimator with triangular kernel weights and the MSE-optimal bandwidth selector. Since each covariate may have a different conditional mean function and different curvature, the optimal bandwidth for local polynomial estimation and inference will also be different for every variable; i.e., the statistical analysis is conducted separately for each covariate using the local polynomial estimators. (ii) the number of effective observations used in the analysis varies for each covariate, which occurs because the MSE-optimal bandwidth is different for every covariate tested for continuity. |  |  |  |  |  |  |  |

Table S27. Manipulation test of running variable

| Method |  | Test statistic | P-value |
| --- | --- | --- | --- |
| Unrestricted |  |  |  |
|  | Conventional | -0.5373 | 0.5911 |
|  | Robust | -0.6405 | 0.5219 |
| Restricted |  |  |  |
|  | Conventional | -0.2731 | 0.7848 |
|  | Robust | -0.2549 | 0.7988 |

*Note*: (a) The manipulation test was constructed using a 2nd-order local-polynomial density estimator, an MSE-optimal bandwidth choice coupled with robust bias-correction, and the corresponding jackknife SE estimator. (b) The RD estimates are based on the conventional RD estimator and the Robust biased-corrected RD estimator with and without restriction. (c) The test is constructed by using “rddensity” statistical package.

Table S28. Placebo cutoff

|  | Females Placebo cutoff at age 50 |  | Placebo cutoff at age 59 for males and 54 for females |  | Placebo cutoff at age 61 for males and 56 for females |  |
| --- | --- | --- | --- | --- | --- | --- |
| Outcomes | estimate | p-value | estimate | p-value | estimate | p-value |
| Self-reported health | 0.2627 | 0.1996 | 0.807 | 0.4304 | 0.2627 | 0.1996 |
| Life Satisfaction | 0.0601 | 0.6502 | 0.1652 | 0.6186 | 0.0599 | 0.6534 |
| CESD Scale | -1.223 | 0.6686 | -11.7595 | 0.1435 | -1.2227 | 0.6682 |
| The CHARLS Cognitive Battery | -1.3207 | 0.1826 | 0.886 | 0.6832 | -1.3138 | 0.1847 |
| ADL | 0.0386 | 0.9208 | -0.4631 | 0.6063 | 0.0388 | 0.9188 |
| IADL | -0.3019 | 0.1478 | -0.7253 | 0.3883 | -0.2978 | 0.1535 |
| Total Recall Score | -1.2272 | 0.5864 | 5.4550 | 0.3121 | -1.2205 | 0.5912 |

Table S29. Fuzzy RD sensitivity test to observations near cutoff: The Donut-Hole Approach

| Donut-hole radius | MSE-optimal bandwidth | Estimate | Robust 95% CI | Excluded observations L│R |
| --- | --- | --- | --- | --- |
| Self-reported Health |  |  |  |  |
| 0 | 3.9 | 0.1374 | [-0.2580, 0.6651] | 0│0 |
| 0.1 | 4.4 | 0.1010 | [-0.3399, 0.6815] | 16│39 |
| 0.2 | 5.1 | 0.1014 | [-0.2955, 0.7022] | 27│48 |
| 0.3 | 3.5 | -0.2648 | [-1.5994, 1.0837] | 118│115 |
|  |  | **Life Satisfaction** |  |  |
| 0 | 3.8 | -0.0567 | [-0.3100, 0.2671] | 0│0 |
| 0.1 | 2.9 | -0.0732 | [-0.3523, 0.2905] | 0│28 |
| 0.2 | 2.9 | -0.0784 | [-0.3322, 0.2522] | 26│41 |
| 0.3 | 2.5 | 0.1074 | [-0.2387, 0.5882] | 109│112 |
| CESD Scale |  |  |  |  |
| 0 | 3.6 | 1.4545 | [-4.8459, 7.3168] | 0│0 |
| 0.1 | 2.9 | 1.0680 | [-5.4796, 6.7111] | 0│29 |
| 0.2 | 2.7 | 1.2889 | [-4.8091, 6.7180] | 26│43 |
| 0.3 | 2.4 | -3.2049 | [-12.0116, 4.1531] | 110│115 |
| The CHARLS Cognitive Battery |  |  |  |  |
| 0 | 3.6 | -0.4198 | [-2.3391, 1.3704] | 0│0 |
| 0.1 | 3.4 | -0.4724 | [-2.4416, 1.3340] | 0│26 |
| 0.2 | 3.2 | -0.4466 | [-2.3756, 1.3820] | 25│38 |
| 0.3 | 2.4 | 1.8145 | [-0.7288, 5.0330] | 102│103 |
| ADL |  |  |  |  |
| 0 | 4 | 0.0404 | [-0.4302, 0.5152] | 0│0 |
| 0.1 | 3.6 | -0.0986 | [-0.5897, 0.3324] | 0│29 |
| 0.2 | 2.9 | -0.2246 | [-0.7626, 0.1943] | 28│44 |
| 0.3 | 2.0 | -0.5259 | [-1.7930, 0.5058] | 120│122 |
| IADL |  |  |  |  |
| 0 | 2.8 | -0.1869 | [-0.8337, 0.2795] | 0│0 |
| 0.1 | 3.9 | -0.0739 | [-0.5574, 0.3672] | 0│27 |
| 0.2 | 3.2 | -0.0997 | [-0.6271, 0.3768] | 28│41 |
| 0.3 | 1.9 | -0.5925 | [-2.0847, 0.6805] | 107│116 |
| Total Recall Score |  |  |  |  |
| 0 | 3.9 | 2.2990 | [-1.0749, 7.1681] | 0│0 |
| 0.1 | 3.0 | 2.2326 | [-2.0861, 7.2232] | 0│26 |
| 0.2 | 2.7 | 3.5337 | [-0.5950, 8.8061] | 26│39 |
| 0.3 | 2.4 | 8.9991 | [1.3692, 20.6469] | 106│108 |

Figure S1. Cutoff age for female employees

*Note*: We used the mimicking variance quantile-spaced method to estimate the sample means.

Figure S2. Self-reported Health RD Plot 

Integrated Mean Square Error (IMSE) approach vs. Mimicking Variance (MV) approach

Figure S3. Life Satisfaction RD Plot 

Integrated Mean Square Error (IMSE) approach vs. Mimicking Variance (MV) approach

Figure S4. CESD Scale RD Plot 

Integrated Mean Square Error (IMSE) approach vs. Mimicking Variance (MV) approach

**Figure S5. The CHARLS Cognitive Battery RD Plot** 

Integrated Mean Square Error (IMSE) approach vs. Mimicking Variance (MV) approach

Figure S6. ADL RD Plot 

Integrated Mean Square Error (IMSE) approach vs. Mimicking Variance (MV) approach

Figure S7. IADL RD Plot 

Integrated Mean Square Error (IMSE) approach vs. Mimicking Variance (MV) approach

Figure S8. Total Recall Score RD Plot 

Integrated Mean Square Error (IMSE) approach vs. Mimicking Variance (MV) approach

**Figure S9. Nonparametric Regression discontinuity robustness of bandwidth choice for self-reported health** 

Figure S10. Nonparametric Regression discontinuity robustness of bandwidth choice for life satisfaction

Figure S11. Nonparametric Regression discontinuity robustness of bandwidth choice for CESD Scale

Figure S12. Nonparametric Regression discontinuity robustness of bandwidth choice for the CHARLS cognitive battery 

Figure S13. Nonparametric Regression discontinuity robustness of bandwidth choice for ADL 

Figure S14. Nonparametric Regression discontinuity robustness of bandwidth choice for IADL 

Figure S15. Nonparametric Regression discontinuity robustness of bandwidth choice for total recall score

Figure S16. Self-reported Health RD Plot by Gender

Figure S17. Life Satisfaction RD Plot by Gender

Figure S18. CESD Scale RD Plot by Gender

Figure S19. The CHARLS Cognitive Battery RD Plot by Gender

Figure S20. ADL RD Plot by Gender

Fig. S21 IADL RD Plot by Gender 

Figure S22. Total Recall Score RD Plot by Gender

Figure S23. Nonparametric Regression discontinuity robustness of bandwidth choice for self-reported health by gender

Figure S24. Nonparametric Regression discontinuity robustness of bandwidth choice for life satisfaction by gender

Fig. S25 Nonparametric Regression discontinuity robustness of bandwidth choice for CESD scale by gender 

Fig. S26 Nonparametric Regression discontinuity robustness of bandwidth choice for the CHARLS cognitive battery by gender

Figure S27. Nonparametric Regression discontinuity robustness of bandwidth choice for ADL by gender

Figure S28. Nonparametric Regression discontinuity robustness of bandwidth choice for IADL by gender

Figure S29. Nonparametric Regression discontinuity robustness of bandwidth choice for Total recall score by gender

Figure S30. Self-reported Health RD Plot by Education

Figure S31. Life Satisfaction RD Plot by Education

Figure S32. CESD Scale RD Plot by Education

Figure S33. the CHARLS Cognitive Battery RD Plot by Education

Figure S34. ADL RD Plot by Education

Figure S35. IADL RD Plot by Education

Figure S36. Total Recall Score RD Plot by Education

Figure S37. Nonparametric Regression discontinuity robustness of bandwidth choice for self-reported health by education

Figure S38. Nonparametric Regression discontinuity robustness of bandwidth choice for life satisfaction by education

Figure S39. Nonparametric Regression discontinuity robustness of bandwidth choice for CESD scale by education

Figure S40. Nonparametric Regression discontinuity robustness of bandwidth choice for the CHARLS cognitive battery by education

Figure S41. Nonparametric Regression discontinuity robustness of bandwidth choice for ADL by education

Figure S42. Nonparametric Regression discontinuity robustness of bandwidth choice for IADL by education

Figure S43. Nonparametric Regression discontinuity robustness of bandwidth choice for Total recall score by education

Figure S44. The predetermined covariates by normalized age

*Note*: We used mimicking variance quantile-spaced method to estimate the sample means.

Figure S45. Running variable density test

*Note:* (a) The manipulation test was constructed using a 2nd-order local-polynomial density estimator, an MSE-optimal bandwidth choice coupled with robust bias-correction, and the corresponding jackknife SE estimator. (b) The solid line represents point estimate, while the shaded area represents 95% CI.
